# Bioactive Bromotyrosine Derivatives from the Pacific Marine Sponge *Suberea clavata* (Pulitzer-Finali, 1982)

**DOI:** 10.3390/md19030143

**Published:** 2021-03-06

**Authors:** Céline Moriou, Damien Lacroix, Sylvain Petek, Amr El-Demerdash, Rozenn Trepos, Tinihauarii Mareva Leu, Cristina Florean, Marc Diederich, Claire Hellio, Cécile Debitus, Ali Al-Mourabit

**Affiliations:** 1 CNRS, Institut de Chimie des Substances Naturelles, Université Paris-Saclay, F-91190 Gif-sur-Yvette, France; celine.moriou@cnrs.fr (C.M.); crespipharma@gmail.com (D.L.); eldemerdash555@gmail.com (A.E.-D.); 2 IRD, CNRS, Ifremer, LEMAR, Univ Brest, F-29280 Plouzane, France; rozenn.trepos@gmail.com (R.T.); claire.hellio@univ-brest.fr (C.H.); cecile.debitus@ird.fr (C.D.); 3 IRD, Ifremer, ILM, EIO, Univ de la Polynésie française, F-98713 Papeete, French Polynesia; mareva.leu@gmail.com; 4 Laboratoire de Biologie Moléculaire et Cellulaire du Cancer, Hôpital Kirchberg, 9, rue Edward Steichen, L-2540 Luxembourg, Luxembourg; cristina.florean@lbmcc.lu; 5 Department of Pharmacy, Research Institute of Pharmaceutical Sciences, College of Pharmacy, Seoul National University, 1 Gwanak-ro, Gwanak-gu, Seoul 08826, Korea; marcdiederich@snu.ac.kr

**Keywords:** sponge, Verongiida, *Suberea clavata*, bromotyrosine, fistularin-3, acetylcholinesterase inhibition, antifouling

## Abstract

Chemical investigation of the South-Pacific marine sponge *Suberea clavata* led to the isolation of eight new bromotyrosine metabolites named subereins 1–8 (**2**–**9**) along with twelve known co-isolated congeners. The detailed configuration determination of the first representative major compound of this family 11-*epi*-fistularin-3 (11*R*,17*S*) (**1**) is described. Their chemical characterization was achieved by HRMS and integrated 1D and 2D NMR (nuclear magnetic resonance) spectroscopic studies and extensive comparison with literature data. For the first time, a complete assignment of the absolute configurations for stereogenic centers C-11/17 of the known members (11*R*,17*S*) 11-*epi*-fistularin-3 (**1**) and 17-deoxyfistularin-3 (**10**) was determined by a combination of chemical modifications, Mosher’s technology, and ECD spectroscopy. Consequently, the absolute configurations of all our new isolated compounds **2**–**9** were determined by the combination of NMR, Mosher’s method, ECD comparison, and chemical modifications. Interestingly, compounds **2**–**7** were obtained by chemical transformation of the major compound 11-*epi*-fistularin-3 (**1).** Evaluation for acetylcholinesterase inhibition (AChE), DNA methyltransferase 1 (DNMT1) modulating activity and antifouling activities using marine bacterial strains are also presented.

## 1. Introduction

The Verongiida order is well known for producing highly diversified brominated metabolites, which are biogenetically linked to tyrosine. A large number of bromotyrosine derivatives, including “dimeric” spiro-isoxazoline alkaloids, phenolic oximino amides, and tetrameric macrocyclic bastadins have been reported [1]. Many of these metabolites possess diverse biological activities and also ecological significance. These specialized metabolites play a crucial role in the interactions between organisms and with their environment in their ecosystem. Bromotyrosines compounds are chemical markers of the Verongiida order [2]. They are of great interest for chemical exploration: although bromotyrosine metabolites were mainly isolated from sponge species belonging to Verongiida order, it is worth to note that fistularin-3 was very recently identified by Nicacion et al. in cultures of the marine bacterium *Pseudovibrio denitrificans* Ab134 isolated from the sponge *Arenosclera brasiliensis* [3]. The very special interest of this result lies in the sustainable supply potential of fistularin for further use. Nevertheless, the association of the bacterium *Pseudovibrio denitrificans* with a sponge from the order Haplosclerida deflects the presence of bromotyrosine specifically in the sponges of the order Verongiida. The identification of the bromotyrosine-derived alkaloids agelorins A-B and 11-*epi*-fistularin-3 from an Australian *Agelas oroides* (order Agelasida) by König et al. [4] is another exception to the idea that these compounds are specifically associated with Verongiida sponges.

From a chemical ecology point of view, some of these polybrominated compounds have been suggested to undergo enzyme-mediated transformation into aeroplysinin-1 and dibromocyclohexadienone which could play an important role in the sponge’s defense mechanism as antifeedant, antifouling, and also in response to tissue damage [5,6]. Regarding pharmacology, bromotyrosine derivatives have been shown to possess a myriad of biological potentialities including antimalarial [7], biocidal antifouling [8,9], antimicrobial [10], antimycobacterial [11], antiviral [12,13], and antifungal activities [14], cytotoxicity [15], specific histamine H-3 receptor antagonist [16], or enzyme inhibitors [17]. Several bromotyrosine derivatives analogs have been chemically synthesized and were able to inhibit human prostate cancer proliferation, invasion, and migration [18].

We previously discovered several brominated tyrosine congeners from the pacific marine sponge *Suberea ianthelliformis* with both chemical and ecological aspects [19]. As a part of our running program on the chemical exploration of pharmacologically active bromotyrosine products from *Suberea* marine sponges genus, we describe herein the isolation and structural assignment of eight new brominated tyrosine-containing metabolites (**2**–**9**), the absolute configuration determination of the known 11-*epi*-fistularin-3 (**1)** and 17-deoxyfistularin-3 (**10**) along with 11 other known derivatives from the sponge *Suberea clavata*. Biological evaluations for antibacterial and acetylcholinesterase (AChE) inhibitory activities and DNA methyltransferase 1 (DNMT1) modulating activity are described as well.

## 2. Results and Discussion

### Isolation and Structure Elucidation

**Figure 1:**
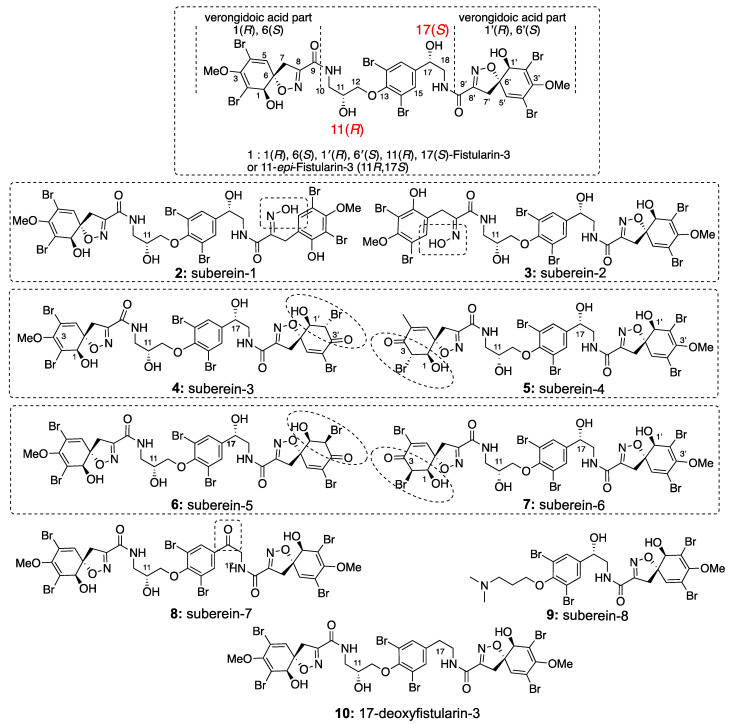
Structures and absolute configurations of the isolated compounds **1**–**10**. The dotted rectangle groups the regioisomers two by two. The absolute configurations of all compounds were confirmed by the combination of NMR, Mosher’s method, and ECD comparison.

The sponge *Suberea clavata* [20,21] was collected in the Solomon Islands, extracted with a mixture of CH_2_Cl_2_/MeOH (1/1), and partitioned between n-BuOH and H_2_O. A part of the butanolic extract (5 g) was submitted to reversed-phase C_18_ SPE and eluted with successive solvents (H_2_O; MeOH; CH_2_Cl_2_) to afford six fractions. Step-wise repetitive purifications using HPLC (high performance liquid chromatography) and SFC (supercritical fluid chromatography) separation methods gave eight new bromotyrosine compounds **2**–**9** (Figure 1) named subereins **1**–**8**, as well as eleven known metabolites (Figure S2, supporting information) such as the major compound of the extract (3 g, 14.3%) 11-*epi*-fistularin-3 (**1**) [4], 17-deoxy-*epi*-fistularin-3 (**10**) [22], agelorins A and B (**11**–**12**) [4], 11-deoxyfistularin-3 (**13**) [23], 11,17-dideoxyfistularin-3 (**14**), 11-hydroxy-aerothionin (**15**) [14], aerophobine 2 (**16**), aplysinamisin-1 (**17**) [24], 7*R*,11*S* [3,5-dibromo-4-[(2-oxo-5-oxazolidinyl)]methoxyphenyl]-2-oxazolidinone (**18**) [25], subereaphenol K (**19**) [26], and pseudoceralidinone A (**20**) [27], whose structures were confirmed by comparison of their spectroscopic data with those reported in the literature. We have recently reported the in vitro inhibition of DNMT1 by our 11-*epi*-fistularin-3 (**1**) and described docking studies of the determined configuration [28].

**Figure 2:**
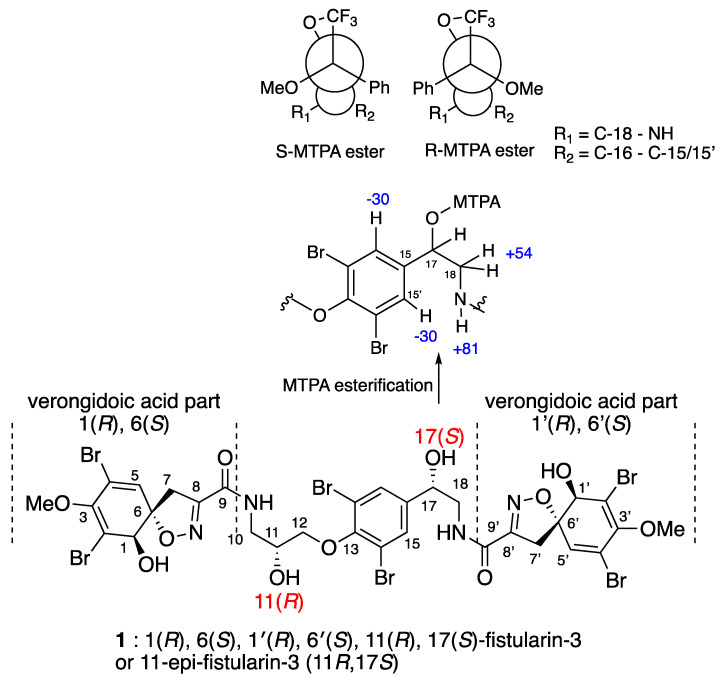
*(+)-1(R), 6(S), 1′(R), 6′(S), 11(R), 17(S)*-fistularin-3 and Mosher AC (representation of the conformation of each MTPA esters and the resulting ∆*^SR^* values (in Hz) for each of the protons).

The structure of the known *epi*-fistularin-3 (**1**) was confirmed by ^1^H and ^13^C NMR (nuclear magnetic resonance) data comparison with the literature [4,29]. In fact, similarities of ^1^H and ^13^C NMR spectra of fistularin-3 stereoisomers allow the room for confusion that exists regarding the absolute configurations of C-11 an C-17 carbons. The latter problem was pointed by Molinsky by studying samples from various sponges [29]. In the latter study, the configuration of C-11 was determined for different epimers. Finally, the C-17 configuration of all fistularin-3 isomers remains still undefined and the term isofistularin-3 is mainly used as generic name for undetermined fistularin-3 isomers so far. While the configuration of the verongidoic acid part (Figure 2) was established as 1(*R*), 6(*S*) for C-1 and C-6 by NMR spectroscopy and CD analysis [4,29,30], the absolute configurations at the two secondary carbinols C-11 and C-17 require precise determination. In the literature, this task was hampered by the presence of four secondary alcohols that significantly complicated the co-determination of their absolute configuration by chemical modification and NMR studies on the native molecule without degradation. Suitable crystals for X-ray studies have never been obtained for any one of the stereoisomers of fistularin-3. Moreover, the carbon configuration also seems to vary depending on sponge species. The name 11-*epi*-fistularin-3 has been used to identify a molecule isolated from *Agelas oroides*, which was recognized as a 11(*R)* [29]. Fistularin-3 extracted from *Aplysina cauliformis* has an 11*S* configuration, leaving the configuration C-17 undetermined. Our contribution starts with the determination of the absolute configuration of both C-11 and C-17. The question that arose for us is which of the two epimers have we isolated by determining the configuration of C-11 on the one hand, and on the other hand if we can determine the configuration of C-17 for the first time.

The isomer that we have isolated **1** (Figure 2) showed similar NMR spectra and an optical rotation of +148.0° (c 1.0, MeOH) or +169.0 (0.2, acetone) compared to +147° (c 0.275, acetone) for fistularin-3. The first point was to make sure that our product is not a mixture of two C-11 epimers, as Molinsky demonstrated with *epi*-fistularin-3 [29]. Indeed, the zoom on C-11 and C-17 (see SI, Figure S3) of our C-13 NMR spectrum shows that our sample is indeed diastereomerically pure. As already noted in the literature, these bromotyrosine metabolites crystallize poorly and do not allow X-ray analysis, and indeed we tried the derivatization of the OH functionalities of **1** with crystallogenic groups such 3,5 dinitrobenzoic acid and camphanic acid without success. For both 1, 1′, 11, 17-tetradinitrobenzoic ester and 1, 1′, 11, 17-tetracamphanic derivatives, we were not able to obtain suitable crystals. We then decided to analyze the stereochemistry by alternative methods.

The known absolute configurations of the asymmetric centers (*R*)C-1, (*R*)C-1′, (*S*)C-6 and (*S*)C-6′ on the spiroisoxazole moiety was confirmed by comparison with NMR data and the ECD spectrum that showed characteristic positive Cotton effects at 253 nm (Δє + 7.5) and 285 nm (Δє + 8.0) [31,32].

Following the method described by Molinsky [29], we hydrolyzed the *epi*-fistularin-3 (**1**) with aqueous trifluoracetic acid at 120 °C overnight to obtain the 1,2-aminopropanediol bearing the C-2 carbinol corresponding to the C-11 of compound **1**. After derivatization with L-FDAA (2,4-dinitrophenyl-1-fluoro-Lalaninamide) and comparison with the C-2 configurations of the derivatized commercial standard aminodiol enantiomers, our sample was found to have the same retention time as the (+)-*R*-aminodiol standard, indicating that the C-11 of compound **1** is *R*.

At this stage, the configuration of the C-17 is still undetermined. To obtain the diastereomeric esters, 20 mg of **1** was reacted with (*R*) and (*S*)-methoxytrifluoromethylphenylacetyl chloride (MTPA-Cl) [33]. The reactions were performed in dichloromethane (DCM) in the presence of triethylamine or pyridine (10 equiv.) and a catalytic amount of 4-dimethylaminopyridine (DMAP). The monoester at position 17 was formed in minute quantity together with other polyesters at positions 1, 11, 17, and 1′. Reversed-phase HPLC (Sunfire C18, 10 × 150 mm, 5 µm; eluting with H_2_O/Acetonitrile (98/2 to 0/100 in 35 min) led to the pure desired monoesters. It should be noted that upon ester formation, the stereochemical descriptor of the MTPA-Cl stereocenter changes, because the COCl group has a Cahn–Ingold–Prelog (CIP) priority different from COOR. The Mosher esters obtained by reacting **1** with (*R*)- and (*S*)-MTPA chloride to yield the (*S*)- and (*R*)-MTPA esters respectively were analyzed using 1D and 2D NMR spectroscopy [33]. The difference in the chemical shift (*δ^SR^*) was calculated for each of the analogous pairs of protons for both the (*S*)- and (*R*)-MTPA esters (Table 1). The positive values were assigned to the left side of the model (R_1_) and the negative values were placed on the right side (R_2_). By applying the Cahn–Ingold–Prelog system, the groups’ priorities were assigned as 1 (OH), 2 (C-18), 3 (C-15/15′), and 4 (H), and the absolute configuration at C-17 was determined as (*S*) for the first time.

**Figure 3:**
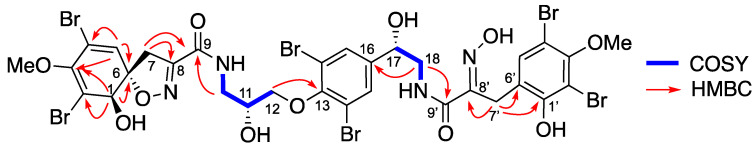
Selected COSY and HMBC correlations for suberein-1 (**2**).

In a slightly less polar fraction, we identified a mixture of two compounds **2** and **3** with the same HRESIMS observed for compound **1**. Pure compounds **2** and **3** were obtained by SFC, using a Cyano column. Compound **2** (Figure 2) was isolated as an amorphous white solid, [α]_D_^25^ + 61.0° (*c* 1.0, MeOH). Its HRESIMS spectrum showed a predominant peak at *m/z* 1136.6924 [M+Na]^+^ corresponding to the molecular formula C_31_H_30_^79^Br_3_^81^Br_3_N_4_O_11_ (calc. for C_31_H_30_Br_6_N_4_O_11_Na: 1136.6849). This isomer of fistularin-3 presented some differences on the ^1^H NMR spectrum. The singlet H-5 expected at δ_H_ 6.55 ppm was not observed, but another one appeared at δ_H_ 7.56 ppm, a chemical shift in accordance with an aromatic proton. An interesting change was observed for the CH_2_-7′ observed at δ_H_ 3.81 ppm as a singlet associated by HSQC with a carbon observed at the low chemical shift δ_C_ 25.5 ppm. The COSY spectrum (Figure 2) displayed cross-peak correlations between the signals at δ_H_ 7.56 and 3.81 corresponding to a ^4^*J* coupling suggesting an aromatization together with one of the spiroisoxazoline opening. The second spiroisoxazoline with its diastereotopic protons at δ_H_ 3.87–3.84/3.22–3.18 was observed. The ^13^C NMR spectrum showed more signals similar to those observed for compound **1**, suggesting its partial modification. The HMBC spectrum showed a key correlation between the protons of the methylene group at δ_H_ 3.81 and the carbons at δ_C_ 155.1(C1′) and 122.1(C6′). Furthermore, the proton H-5′ at δ_H_ 7.56 was also correlated to C-1′, C-6′, and to those at δ_C_ 106.3 (C-4′) and 108.9 (C-2′). The COSY spectrum (recorded in acetone-*d*_6_) revealed a spin system including a methylene group CH_2_-10 at δ_H_ 3.78/3.52, followed by an -NH proton at δ_H_ 7.62 and oxymethine CH-11 at δ_H_ 4.24, and terminated with two diastereotopic protons CH_2_-12 at δ_H_ 4.02/4.05. The HMBC spectrum displayed key correlations between H-10 and H-7 with the amidic carbonyl C-9 at δ_C_ 160.4 (Figure 3), which confirmed that the left-hand side of the molecule was retained is an oxazolinic motif like in compound **1**. Further significant HMBC correlations were observed between CH_2_-12 δ_H_ 4.02/4.05 and C-13 at δ_C_ 152.7. Additionally, the COSY spectrum allowed us to identify a second partial structure including the oxymethine H-17 at δ_H_ 4.92, two diastereotopic protons at δ_H_ 3.64/3.49, and -NH at δ_H_ 8.05 ppm. The proton CH-17 at δ_H_ 4.92 displayed key HMBC corrections with C-15/15′ and C-16 at δ_C_ 131.5 and 143.1, respectively. Furthermore, the methylene groups CH_2_-18 at δ_H_ 3.64/3.49 and CH_2_-7′ at δ_H_ 3.85/3.19 exhibited additional HMBC correlations with the quaternary carbon C-9′ at δ_C_ 166.7, which confirmed the planar structure of the compound **2**. The stereochemistry of the spiroisoxazoline moiety was determined as 1*R*, 6*S* by its ECD spectrum, which showed characteristic positive Cotton effects at 256 nm (Δє + 2.55) and 290 nm (Δє + 2.17). Compound **2** was named suberein-1.

**Figure 4:**
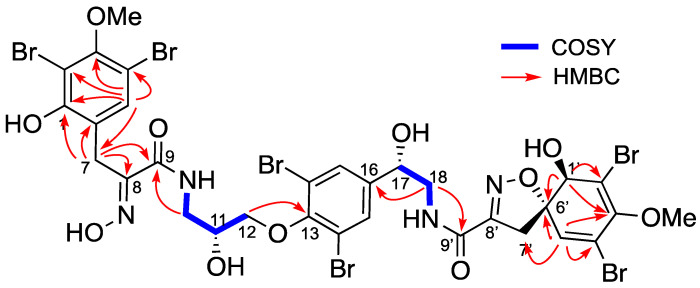
Selected COSY and HMBC correlations for suberein-2 (**3**).

Compound **3** (Figure 4) was a regioisomer of compound **2** ([α]_D_^25^ + 70.7° (*c* 1.0, MeOH)). The HRESIMS spectrum showed the predominant peak at *m/z* 1136.6864 [M+Na]^+^, corresponding to a molecular formula C_31_H_30_Br_6_N_4_O_11_ (calc. for C_31_H_30_Br_6_N_4_O_11_Na, 1136.6849). The ^1^H and ^13^C NMR spectral data (Table 2) were similar to those of compound **2** and suggested the opening of the other spiroisoxazoline moiety. This was confirmed by the key HMBC correlations between the methylene groups CH_2_-7 at δ_H_ 3.84 and CH_2_-10 at δ_H_ 3.78/3.58 with the amidic carbonyl C-9 at δ_C_ 166.6 (Figure 4). The absolute configuration of the spiroisoxazoline moiety was determined as 1*R*, 6*S* based on positive Cotton effects at 255 nm (Δє + 2.33) and 286 nm (Δє + 2.19), in its ECD spectrum. Compound **3** was named suberein-2.

**Figure 5:**
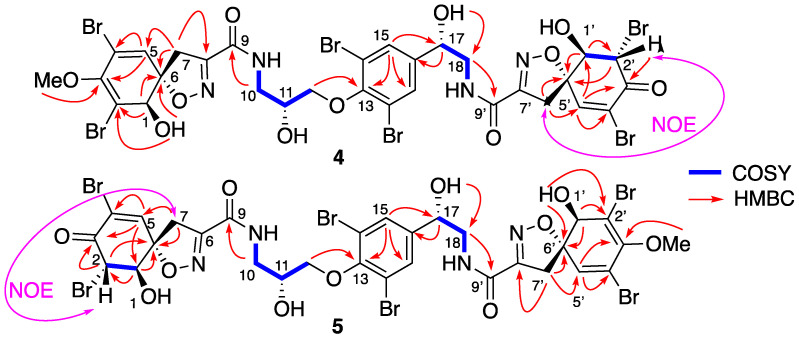
Key COSY, HMBC, and NOE correlations differentiating suberein-3 (**4**) and suberein-4 (**5**).

Compounds **4** + **5** were isolated together as an inseparable 1/1 mixture. Their HRESIMS spectrum exhibited a predominant peak at *m/z* 1100.6935 [M+H]^+^ corresponding to a molecular formula C_30_H_28_Br_6_N_4_O_11_ (calc. for C_30_H_29_Br_6_N_4_O_11_, 1100.6872), which indicated 14 a.m.u less than 11-*epi*-fistularin-3 (**1**). The ^1^H NMR spectrum (Table 3) showed only one methyl group as a singlet at δ_H_ 3.73 instead of two in 11-*epi*-fistularin-3 (**1**). Indeed, the ^13^C NMR spectrum disclosed two characteristic resonances at δ_C_ 183.6 (C-3′) assigned to a carbonyl group, and δ_C_ 75.3 attributed to C-1′, which were not recorded for 11-*epi*-fistularin-3 (**1**). The COSY spectrum showed key correlations between the proton at δ_H_ 5.08 (H-2′) and the oxymethine at δ_H_ 4.41 (H-1′), with a coupling constant *J* = 11.4 Hz. Significant HMBC correlations were observed between the proton H-5′ and the carbons at δ_C_ 75.3 (C-1′), 57.4 (C-2′), 183.6 (C-3′), 122.6 (C-4′), and 91.8 (C-6′). Additionally, the oxymethine H-1′ was correlated with C-2′ and meanwhile, H-2′ was correlated with C-3′. Thus, the structure of **4** was determined as described in Figure 5. The same demethoxylated substructure was observed on its isomer **5** in the left side of the molecule (C-1 to C-6 instead of C-1′ to C-6′). The observed NOE correlation between H-2′ and one of the H-5′ together with the absence of NOE correlations between H-1′ and H2′ suggested a *trans* configuration for **4** + **5**. Compounds **4** and **5** are named suberein-3 and suberein-4, respectively.

**Figure 6:**
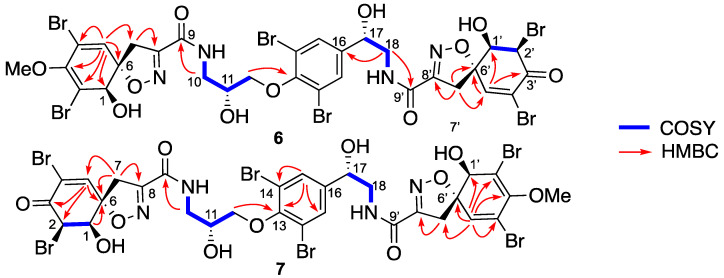
Key COSY and HMBC correlations of suberein-5 (**6**) and suberein-6 (**7**).

As for **4** + **5**, the following compounds **6** + **7** were also isolated together as an inseparable in 1/1 mixture. Their molecular formulas were established as C_30_H_28_Br_6_N_4_O_11_ from their positive HRESIMS spectrum with a predominant peak at *m/z* 1100.6935 [M+H]^+^, (calc. for C_30_H_29_^79^Br_3_^81^Br_6_N_4_O_11_, 1100.6872). Their ^1^H NMR spectrum (Table 4) showed strong similarities with their stereoisomers **4** + **5**, but the main difference appeared in the chemical shift of H-5/H-5′, H-1/H-1′ and H-2/H-2′. The coupling constant between H-1/H-1′ and H-2/H-2′ was close very weak (br s) suggesting a *cis* configuration, which was further confirmed by the NOE correlations. Therefore, the structure of **6** and **7** were assigned as described in Figure 6 and named suberein-6 and suberein-7, respectively.

It should be noted that due to the keto-enolic equilibrium, carbons C-2 and C-2′ in both mixtures **4** + **5** and **6** + **7** epimerize within two days in solution into a mixture of **4**/**5**/**6**/**7**: 1/1/1/1. Their purification just allowed their characterisation as mixtures of the structures of the *cis* stereoisomers on the one hand and of the *trans* stereoisomers on the other hand.

**Figure 7:**
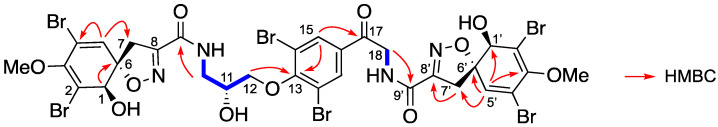
Key HMBC correlations for suberein-7 (**8**).

Compound **8** was isolated as an amorphous powder ([α]_D_^25^ + 149.5° (*c* 0.5, MeOH)). Its HRESIMS showed an isotopic cluster indicating six bromines with a principal peak at *m/z* 1110.6661 [M−H]^−^ corresponding to a molecular formula C_31_H_28_Br_6_N_4_O_11_ (calc. for C_31_H_27_Br_6_N_4_O_11_, 1110.6715). Its ^1^H NMR spectrum (Table 2) showed the characteristic signals of the fistularin-3 with some modifications, including the presence of a doublet at δ_H_ 4.87 (H-18) together with the absence of the H-17 signal observed for fistularin-3 at δ_H_ 4.90. The chemical shift of the aromatic protons (H-15/15′) was observed at δ_H_ 8.28. The ^13^C NMR spectrum (Table 2) showed a quaternary carbon at δ_C_ 192.2, characteristic for a carbonyl group. The HMBC spectrum displayed significant correlations between H_2_-18 (δ_H_ 4.87), H-15/15′ (δ_H_ 8.28), and the non-protonated carbon at δ_C_ 192.2, thus assigning the carbonyl group to C-17. Further key HMBC correlations were consistent with the backbone of fistularin-3. Thus, the structure was depicted as 17-oxo-11-*epi*-fistularin-3 for compound **8** and named suberein-7 (Figure 7), a new member of the bromotyrosine-derived compounds. 

**Figure 8:**
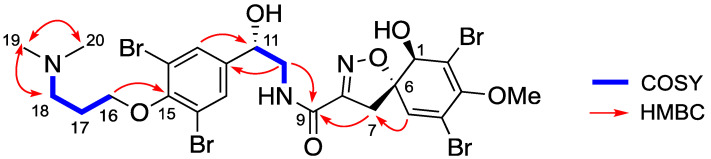
Key COSY and HMBC correlations for suberein-8 (**9**).

Compound **9** ([α]_D_^25^ + 84.0° (*c* 0.2, MeOH)) showed in its HRESIMS an isotopic cluster of peaks indicating a tetrabrominated compound with a principal peak at *m/z* 761.8695 [M+H]^+^ corresponding to the molecular formula C_23_H_26_Br_4_N_3_O_6_ (calc. for C_23_H_28_Br_4_N_3_O_6_, 761.8671). The ^1^H NMR spectrum (Table 5) disclosed characteristic signals with similar chemical shifts to those reported for the right-hand side of *epi*-fistularin-3 (**1**). The COSY spectrum allowed the identification of additional spin system including an oxymethylene group (CH_2_-16) at δ_H_ 4.07 based on its chemical shift and CH_2_ group at δ_H_ 2.11 (CH_2_-17), and terminated with an azomethylene group (CH_2_-18) at δ_H_ 2.87. A singlet at δ_H_ 2.47 integrated for six protons was assigned to the *N,N*-dimethyl terminus (CH_3_-19/20). The HMBC spectrum displayed key correlations between the oxymethylene group at δ_H_ 4.07 (CH_2_-16) and the quaternary carbon C-15 at δ_C_ 152.1, linking this partial structure to the tetrasubstituted phenyl ring. Furthermore, the signal corresponding to the *N,N*-dimethyl functionality (δ_H_ 2.47, CH_3_-19/20) displayed HMBC correlation with the carbon C-18 at δ_C_ 56.0. The other HMBC correlations were consistent with the proposed structure for **9** and named suberein-8 (Figure 8).

**Figure 9:**
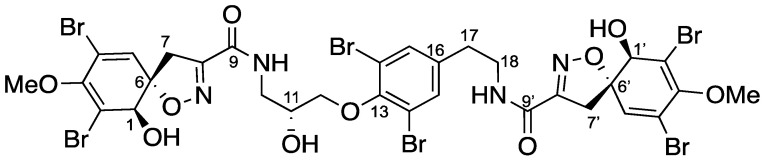
Key HMBC correlations of 17-deoxy-11(*R*)-*epi*-fistularin-3 (**10**).

Compound **10** ([α]_D_^25^ + 71.0° (*c* 0.3, MeOH)) showed in its negative HRESIMS a characteristic isotopic cluster of a hexabrominated compound with a predominant peak at *m/z* 1096.6923 [M−H]^−^ corresponding to the molecular formula C_31_H_30_Br_6_N_4_O_10_ (calc. for C_31_H_29_Br_6_N_4_O_10_, 1096.6923). Its ^1^H NMR spectral pattern was identical to the one of 17-deoxyfistularin-3 [21]. However, the absolute configuration of C-11 was not determined. As for compound *epi*-fistularin (**1**) described above, placing 17-deoxyfistularin-3 (**10)** after hydrolysis in aqueous trifluoroacetic acid to obtain the 1,2-aminopropanediol and further Marfey’s derivatization and comparison with *R* and *S* 1,2-aminopropanediol standards, we found that the absolute configuration of C-11 was *R*. Thus, compound **10** was assigned as a 17-deoxy-11(*R*)-*epi*-fistularin-3 (Figure 9).

The presence of agelorins and compounds **2**–**8** with 11-*epi*-fistularin (**1**) in our sponge brings us to the question of which one the precursor is, and if degradation processes can take place during purification. This is interesting because all the reactions involved are simple and can be imagined without any particular catalysis. Hydrolysis and simple oxidation of the benzylic alcohol into ketone can take place spontaneously. The presence of water, oxygen, and the variation of the acid–base conditions are sufficient. As noted above, such transformations were previously observed for agelorins A-B (**11**–**12**) which were isolated separately with a smaller retention time [4,29]. Rogers et al. identified a mixture of these two products in a sample of *epi*-fistularin-3 (**1**) after storing for several years at −20 °C [29]. When *epi*-fistularin-3 is heated in acidic conditions, several degradation compounds appeared. We then decided to manage the degradation process of *epi*-fistularin-3 (**1**) in a less drastic conditions by dissolving it in a mixture of acetonitrile/water (+0.1% HCOOH) 1/1 and stirring at room temperature for two weeks. The reaction mixture was then dried under vacuum and chromatographed. In addition to the formation of agelorin A and agelorin B, we identified compounds **2**–**7** as well. This result raises the interesting question as to whether these compounds are natural or simply degradation derivatives of 11-*epi*-fistularin-3 (**1**).

## 3. Biological Activities

### 3.1. Antibacterial Activity Inhibition

Minimum inhibitory concentration (MIC values) results are presented in Table 6. Compounds were tested against a panel of bacterial strains commonly used for assessment of their anti-biofilm properties [34]. None of the compounds tested displayed antibacterial activity (at the concentrations tested) against all the panel of bacteria tested (four marine bacteria): *Vibrio natriegens*, *Shewanella putrefaciens*, *Pseudoalteromonas elyakovii, Polaribacter irgensii;* and two terrestrial bacteria: *Staphylococcus aureus* and *Pseudomonas aeruginosa*. Moreover, among all the tested compounds, no activity was observed (at the tested concentrations) for 11-*epi*-fistularin-3 (**1**), compounds **4** and **5**, 11-hydroxyaerothionin (**15**), and aplysinamin-1 (**17**).

The most active molecule was compound **3**, which displayed an MIC value of 0.01 µg/mL (the lowest concentration tested) against *V. aesturianus* and *E. coli*. These results are of great importance as new inhibitors against these strains are sought-after actively by scientists, and indeed not only for antifouling purposes. *E. coli* is a common inhabitant of the human gut microbiota and commonly causes nosocomial infection, urinary tract infections, neonate meningitis, and bacteria-related diarrhea. Resistance to antimicrobial agents (e.g., broad-spectrum penicillin and trimethoprim or third generation cephalosporin and nitrofurantoin) in *E. coli* has been reported worldwide and has an increasing impact on available therapeutic options [35]. There is an urgent need for new compounds inhibiting the growth of *E. coli*. The inhibition of *V. aesturianus* is of high importance as well as this bacterial strain is involved in surface colonization, but it is as well a known pathogen of the commercial oyster *Crassostrea gigas* [36,37,38]. Compounds **2**–**4** could possibly be used in aquaculture for the prevention of diseases. The oyster industry has grown to be very important in many regions of the world contributing substantially to social and economic activity in coastal zones. Abnormal mortality events due to outbreaks of *V. aesturianus* have been reported increasingly since 2008, leading to very high levels of mortality which were sudden and severe (up to 100%), and affected essentially spats (oysters less than one year old) and juveniles (12- to 18-month-old oysters). Massive mortality outbreaks resulted in a shortage in supplies of the shellfish over the next years [39].

It is worth mentioning that the mono- or di-deoxyfistularins (11-deoxyfistularin-3 (**13**), 11,17-dideoxyfistularin-3 (**15**)) displayed MIC values of 0.01 µg/mL (the lowest concentration tested) specifically toward *Halomonas aquamarina*. The discovery of *H. aquamarina* inhibitors is also of great interest since this strain is involved in both marine biofilm formation [40,41] and pathogenicity towards lobster *Homarus americanus,* an important species in aquaculture [42]. *Halomonas aquamarina* also poses a threat as a potential invasive species because it can lead to potential invasion when transported on biofilms inside ballast water tanks [43].

Molecules with targeted activities are of major interest because they make it possible to fight specifically against targeted bacteria without altering non-targeted organisms. This significantly slows down the development of resistance.

### 3.2. Acetylcholinesterase Activity Inhibition

The activity of these compounds was also evaluated on acetylcholinesterase, which is responsible in terrestrial invertebrates for various primordial biosynthetic pathways, the inhibition of which can be lethal for insect species and thus represents a great phytosanitary interest [44,45], and also might be a toxicity indicator in the marine environment, especially in bivalves [46].

The involvement of neurotransmission inhibition through acetylcholinesterase inhibition has been shown in a settlement of cyprid larvae of barnacles [47,48]. Agelorin A (**12**) displayed a better activity than the reference compound Galantamine used as a control for the bioassay (IC_50_ = 0.7 ± 0.1 µM). The acetylcholinesterase inhibition of bromotyrosine derivatives enhances their interest as efficient antifouling natural products especially in their natural environment as a mode of chemical defense.

### 3.3. DNMT1 Activity Inhibition

**Figure 10:**
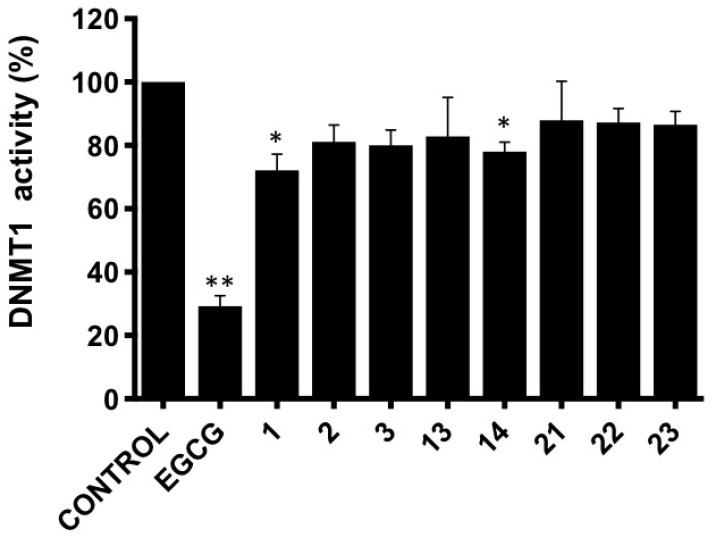
In vitro DNMT1 activity inhibition assays with 11-*epi*-fistularin (**1**), compounds (**2**, **3**, **13**, and **14**), and psammaplysene D (**21**), F (**22**), and G (**23**) (all compounds are used at 50µM). The graph corresponds to the mean ± SD of three independent experiments. * *p* < 0.05; ** *p* < 0.01 (paired repeated measure one-way Anova with Dunnet’s multiple comparison test).

Aberrant DNA methylation is a hallmark of cancer cells, and the enzyme DNA methyltransferase 1 (DNMT1) represents a recognized target in cancer treatment. As a continuation of our previously demonstrated potential of 11-*epi*-fistularin (**1**) to bind DNMT1 and reduce its activity [28], and that of isofistularin-3 extracted from the sponge *Aplysina aerophoba*, and its ability to inhibit DNMT1 leading to DNA demethylation in leukemia cells [49], we also investigated the capacity of our compounds **2**, **3**, **13**, and **14** to modulate the activity of DNMT1 (Figure 10). Results were compared to bromotyrosine compounds psammaplysene D (**21**), F (**22**), and G (**23**) (SI Figure S0), previously isolated from another sponge species in our group, *Suberea ianthelliformis* [19].

In line with our previous results, compound **1** reduced DNMT1 activity to around 70% of control values and compound **14** also provided a statistically significant decrease.

In summary of this manuscript, the chemical examination of the Pacific marine sponge *Suberea clavata* led to the isolation and identification of twenty structurally diverse brominated tryrosine metabolites, of which eight are new congeners, subereins-1–8 (**2**–**9**), along with two major co-isolated known products 11-*epi*-fistularin-3 (**1**) and 17-deoxyfistularin-3 (**10**). A combination of chemical modifications, Mosher’s technology, and ECD spectroscopy allowed the complete assignment of the absolute configurations of the new compounds on one hand, and the assignment of the stereogenic centers C-11/17 of the known congeners (11*R*,17*S*) 11-*epi*-fistularin-3 (**1**) and 17-deoxyfistularin-3 (**10**) for the first time on the other hand.

This solves the problem of the absolute configuration of the C-17 of 11-*epi*-fistularin-3 (**1**), which has remained undetermined since 1993 [4]. Whereas this metabolite is predominant in several sponges and presents interesting biological activities, the easily observed transformation of the major compound 11-*epi*-fistularin-3 (**1**) into subereins 1–6 (**2**–**7**) rise the question of artifacts of extraction. We believe that all compounds are also present in the sponge since they are present in the crud extract of the sponge. This does not exclude their partial formation during chromatographic purifications. The isolated compounds **3**, **13,** and **15** displayed antimicrobial inhibition; **12** displayed acetylcholinesterase inhibition; and **1** and **14** inhibited DNMT1.

## 4. Materials and Methods

### 4.1. General Experimental Procedure

Optical rotations [α]_D_ were measured at 25 °C on a Jasco P-1010 polarimeter (JASCO, Lisses, France). Electronic circular dichroism (ECD) experiments were performed at 25 °C on a JASCO J-810 spectropolarimeter (JASCO, Lisses, France). IR spectra were recorded on a Perkin Elmer BX FT-IR spectrometer (Perkin Elmer, Villebon-sur-Yvette, France). The NMR spectra were recorded on a Bruker 300 MHz instrument (Avance 300), a Bruker 500 MHz instrument (Avance 500,) and a Bruker DRX 600 MHz with a 5 mm or 1.7 mm triple resonance (HCN) probe (Bruker, Wissembourg, France). The chemical shifts are reported in ppm relative to the residual signal solvent (MeOH-*d*_4_: *δ*_H_ 3.31; δ_C_ 49.15; DMF-*d*_7_: *δ*_H_ 8.03, 2.92, 2.75; δ_C_ 163.15, 34.89, 29.76; acetone-*d*_6_: δ_H_ 2.05; δ_C_ 206.68, 29.92). High-resolution mass spectra were obtained with a hybrid linear trap/orbitrap mass spectrometer (LTQ-orbitrap, Thermofisher, Illkirch, France) in electrospray ionization mode by direct infusion of the purified compounds. MPLC was performed using a Combiflash-Companion apparatus (Serlabo Technologies, Entraigues-sur-la-Sorgue, France) and a prepacked C_18_ Versapak cartridge. Preparative HPLC purifications were performed on an autoprep system (Waters 600 controller and Waters 600 pump with a Waters 996 photodiode array detector (Waters France, Guyancourt, France)), equipped with a Waters Atlantis T3 (19 × 150 mm, 5 μm) column, and a Waters Sunfire C_18_ (19 × 150 mm, 5 μm) column or a X-bridge RP-18 (19 × 150 mm, 5 μm) column. SFC purifications were performed on a Thar Waters SFC Investigator II with a Waters 2998 photodiode array detector, equipped with a SFC 2-Ethyl-pyridine (10 × 250 mm, 6 μm) column or a Thar Cyano (10 × 250, 6 μm) column. Analytical UHPLC was performed on UPLC Waters Acquity with PDA, DEDL, and TQD, using a HSS C_18_ (2.1 × 50 mm, 1.8 μm) column. All other chemicals and solvents were purchased from SDS (France).

### 4.2. Biological Material

The sponge was collected by hand using SCUBA in the Solomon Islands, Russell Group, during the sampling cruise BSMS-1 [50] aboard the R/V Alis, off the coast of Lologhan Island (30 June 2004, 9°06.658′ S; 159°21.664′ E) between 6 and 12 m deep. A voucher sample is deposited at the Queensland Museum (Brisbane, Australia) under the access number G322641 and was identified by Dr. J. N. A. Hooper as *Suberea clavata*. The sponge was deep frozen on board until work up. It was then grounded, freeze-dried, and extracted.

### 4.3. Isolation and Spectroscopic Data

#### 4.3.1. Isolation

The lyophilized sponge *Suberea clavata* (200 g) was extracted at room temperature with MeOH/DCM (1/1) to give 57.8 g of dried extract. The crude extract was submitted to *n*-butanol/H_2_O partition to obtain a desalted butanol extract (30 g). A portion (25 g) of the later extract was submitted to reversed-phase C_18_ SPE (50 μm, 65 A, Phenomenex Sepra) and eluted with a gradient H_2_O/MeOH (100/0 to 0/100) and MeOH/DCM (100/0 to 0/100) to give six fractions f1 to f6. The fraction f4 (10 g) was submitted to reversed-phase HPLC (Waters Sunfire C_18_ column, H_2_O + 0.1% HCOOH/CH_3_CN + 0.1% HCOOH: 85/15 to 0/100 in 40 min) yielding 13 subfractions, F1 to F13. The sub-fraction F1 afforded pure aerophobin-2 (**16**) (140 mg, R_T_ = 10 min).

Fraction F6 only contained 11-*epi*-fistularin-3 (**1)** (2.7 g, R_T_ = 21.8 min) and F9 pure 11,17-dideoxyfistularin-3 (**14**) (220 mg, R_T_ = 25.6 min).

Fraction F7 was purified by SFC (ethylpyridine column, CO_2_/MeOH (75/25), isocratic condition) and yielded 17-deoxy-*epi*-fistularin-3 (**10**) (8 mg, R_T_ = 12.0 min), 11-deoxyfistularin-3 (**14**) (36 mg, R_T_ = 11.5 min), and 17-oxo-11-*epi*-fistularin-3 (**8**) (9 mg, R_T_ = 14.1 min).

Fraction F8 was purified by SFC (cyano column, CO2/iPrOH (75/25), isocratic condition) to yield 11-*epi*-fistularin-3 (**1**), suberein-1 (**2**) (10 mg, R_T_ = 11.8 min), and suberein-1 (**3**) (10 mg, R_T_ = 13.0 min).

Fraction F5 was submitted to reversed-phase HPLC (Waters Sunfire C_18_, H_2_O + 0.1% HCOOH/CH_3_CN + 0.1% HCOOH, 85/15 to 25/75 in 25 min). Agelorin A (**11**) (3 mg, R_T_ = 19.6) and B (**12**) (3 mg, R_T_ = 20.1), 11-hydroxyaerothionin (**15**) (2 mg, R_T =_ 22.2 min), as well as a mixture of **4** + **5** (5 mg, R_T_ = 20.9 min) and a mixture of **6** + **7** (5 mg, R_T =_ 21.2 min), were obtained.

Fraction F1 (1.5 g) was purified by MPLC (Versapak C_18_ silica-gel cartridge (23 × 110 mm); H_2_O + 0.1% HCOOH/MeOH+0.1% HCOOH, 100/0 to 0/100, in 35 min). It yielded eight subfractions F’1 to F’8. Pseudoceralidinone A (**20**) (5 mg, R_T_ = 3.5 min) was obtained from F’1. Fraction F’7 was submitted to reversed-phase HPLC (Atlantis T3 C_18_, H_2_O + 0.1% HCOOH/CH_3_CN + 0.1% HCOOH, 100/0 to 75/25 in 7 min, 75/25 to 50/50 in 18 min) to afford aplysinamisin-1 (**17**) (15 mg, R_T_ = 10.5 min), subereaphenol K (**19**) (4 mg, R_T_ = 11.1 min), and 7*R*, 11*S* [3,5-dibromo-4-[(2-oxo-5-oxozolidinyl)]methoxyphenyl]-2-oxazolidinone (**18**) (17 mg, R_T_ = 12.5 min). Fraction F’8 afforded suberein-8 (**9**) (3 mg, R_T_ = 24.0 min) by reversed-phase HPLC purification using an Xbridge C_18_ column (pH 10.5, H_2_O + 0.15%NH_4_OH)/ CH_3_CN + 0.15% NH_4_OH, 90/10 to 0/100 in 35 min).

#### 4.3.2. Spectroscopic Data and Absolute Configurations Determination 

17*S*-*epi*-fistularin-3 (**1**): yellowish amorphous solid (3 g); [α]_D_^25^ + 148.0° (c 1.0, MeOH); [α]_D_^25^ + 169.0 (*c* 0.2, acetone); ECD (0.15 mM, MeOH) λmax (Δє) 253 (+7.5), 285 (+8.0); 1H NMR (500 MHz, acetone-*d*_6_): δ_H_ ppm 7.67 (m, 1H, NH’), 7.66 (s, 2H, H-15, H-15′), 7.62 (m, 1H, NH), 6.53 (d, 2H, H-5, H-5′), 5.41 (d, *J* = 8.0 Hz, 2H, OH-1, OH-1′), 5.00 (d, *J* = 4.3 Hz, 1H, OH-17), 4.90 (dd, *J* = 7.7, 5.5, 4.3 Hz, 1H, H-17), 4.44 (d, *J* = 5.3 Hz, 1H, OH-11), 4.25 (m, 1H, H-11), 4.18 (dd, *J* = 8.0 Hz, 2H, H-1, H-1′), 4.04 (m, 2H, H-12), 3.85 (d, *J* = 18.0 Hz, 1H, H-7a), 3.82 (d, *J* = 18.0 Hz, 1H, H-7a’), 3.80 (m, 1H, H-10a), 3.73 (s, 6H, OCH3, OCH3′), 3.63 (m, 1H, H-18a), 3.54 (m, 1H, H-10b), 3.49 (m, 1H, H-18b), 3.19 (d, *J* = 18.0 Hz, 1H, H-7b), 3.16 (d, *J* = 18.0 Hz, 1H, H-7b’); 13C NMR (125 MHz, acetone-*d*_6_): δ_C_ ppm 160.5 (C-9, C-9′), 155.2 (C-8′), 155.1 (C-8), 152.7 (C-13), 148.8 (C-3, C-3′), 143.3 (C-16), 132.4 (C-5′), 132.3 (C-5), 131.5 (C-15, C-15′), 122.1 (C-4, C-4′), 118.4 (C-14, C14′), 113.9 (C-2′), 113.8 (C-2), 91.8 (C-6, C-6′), 75.9 (C-12), 75.2 (C-1, C-1′), 71.0 (C-17), 69.9 (C-11), 60.2 (OCH3, OCH3′), 47.7 (C-18), 43.6 (C-10), 40.0 (C-7, C-7′); HRESIMS *m/z* 1136.6934 [M+Na]^+^ (calc. for C_31_H_29_Br_6_N_4_O_11_Na, 1136.6847).

##### Determination of the C-17 Configuration for 11-*epi*-fistularin-3 (**1**)

To a stirred solution of compound **1** (20 mg, 0.018 mmol) and anhydrous Pyridine (15 μL, 0.18 mmol, 10 equiv.) in anhydrous DCM (270 μL, [1] = 0.066 M) at room temperature, R-(-)-MTPA-Cl (34 μL, 0.18 mmol, 10 equiv.) and DMAP (0.5 mg) were added. The reaction progress was monitored by thin-layer chromatography (TLC) on silica gel (95:5 DCM/MeOH). After 3 h, compound **1** was totally consumed. The reaction mixture was quenched by the addition of water and EtOAc. The aqueous layer was extracted with two additional portions of EtOAc, and the combined organic layers were dried on MgSO_4_, filtered, and concentrated in vacuo. The crude product mixture was purified by reversed-phase HPLC (Sunfire C_18_, 10 × 150 mm, 5 μm; eluting with H_2_O/acetonitrile (98/2 to 0/100 in 25 min, R_T_ = 22.3 min.)) to give the *S*-MTPA-*epi*-fistularin-3 ester (2.6 mg, 11% yield) as a white solid: ^1^H NMR (300 MHz, acetone-*d*_6_): δ_H_ 7.95 (m, 1H, NH’), 7.63 (m, 3H, NH’, H-15, H-15′), 7.46 (m, 4H, MTPA), 7.40 (m, 1H, MTPA), 6.52 (dd, *J* = 9, 0.7 Hz, 2H, H-5, H5′), 6.13 (dd, *J* = 8.5, 4.0 Hz, 1H, H-17), 5.42 (m, 2H, OH-1, OH-1′), 4.48 (m, 1H, OH-11), 4.25 (m, 1H, H-11), 4.18 (m, 2H, H-1, H-1′), 4.08 (m, 2H, H-12), 3.98 (m, 2H, H-18), 3.88–3.82 (d, *J* = 18 Hz, 1H, H-7), 3.86–3.80 (d, *J* = 18 Hz, 1H, H-7′), 3.82 (m, 1H, H-10a), 3.73 (d, 6H, 3-OCH_3_, 3′-OCH_3_), 3.62 (m, 3H, MTPA-OCH_3_), 3.55 (m, 1H, H-10b), 3.23–3.17 (d, *J* = 18 Hz, 1H, H-7), 3.19–3.13 (d, *J* = 18 Hz, 1H, H-7′); HRESIMS *m/z* 1352.7357 [M+Na]^+^, (calc. for C_41_H_37_^79^Br_3_^81^Br_3_F_3_N_4_O_13_Na, 1352.7246).

To a stirred solution of compound **1** (20 mg, 0,018 mmol) and anhydrous Pyridine (15 mL, 0.18 mmol, 10 equiv.) in anhydrous DCM (270 μL, (1) = 0.066 M) at room temperature, S-(+)-MTPA-Cl (34 μL, 0.18 mmol, 10 equiv.) and DMAP (0.5 mg) were added. The reaction progress was monitored by thin-layer chromatography (TLC) on silica gel (95:5 DCM/MeOH). After 4 h, compound **1** was totally consumed. The reaction mixture was quenched by the addition of water and EtOAc. The aqueous layer was extracted with two additional portions of EtOAc, and the combined organic layers were dried on MgSO_4_, filtered, and concentrated in vacuo. The crude product mixture was purified by reversed-phase HPLC (Sunfire C_18_, 10 × 150 mm, 5 μm; eluting with H_2_O/acetonitrile (98/2 to 0/100 in 25 min, R_T_ = 21.9 min.)) to give the *R*-MTPA-*epi*-fistularin-3 ester (3.2 mg, 13% yield) as a white solid: ^1^H NMR (300 MHz, acetone-*d*_6_): δ_H_ 7.73 (s, 2H, H-15, H15′), 7.68 (m, 2H, NH, NH’), 7.48 (m, 4H, MTPA,), 7.30 (m, 1H, MTPA), 6.52 (dd, *J* = 11, 0.9 Hz, 2H, H-5, H5′), 6.21 (dd, *J* = 8, 5 Hz, 1H, H-17), 5.43 (dd, *J* = 8.5, 3.0 Hz, 2H, OH-1, OH-1′), 4.49 (m, 1H, OH-11), 4.26 (m, 1H, H-11), 4.17 (m, 2H, H-1, H-1′), 4.09 (m, 2H, H-12), 3.88–3.83 (d, *J* = 18.3 Hz, 1H, H-7), 3.82–3.77 (d, *J* = 18.3 Hz, 1H, H-7′), 3.80 (m, 1H, H-18), 3.78 (m, 1H, H-10a), 3.73 (d, 6H, 3-OCH_3_, 3′-OCH_3_), 3.60 (m, 1H, H-10b), 3.50 (s, 3H, MTPA-OCH_3_), 3.23–3.16 (d, *J* = 18.5 Hz, 1H, H-7), 3.12–3.06 (d, *J* = 18.5 Hz, 1H, H-7′); HRESIMS *m/z* 1352.7282 [M+Na]^+^ (calc. for C_41_H_37_^79^Br_3_^81^Br_3_F_3_N_4_O_13_Na, 1352.7246).

Suberein-1 (**2**): colourless amorphous solid (10 mg); [α]_D_^25^ + 61.0 (*c* 1.0, MeOH); [α]_D_^25^ + 32.5 (*c* 0.2, acetone); UV (MeOH) λ_max_ (є) 208 (38200), 286 (10100) nm; ECD (**0**.15 mM, MeOH) λ_max_ (Δє) 214 (−2.79), 256 (2.55), 290 (2.17) nm; IR ν_max_ 3380, 2990, 2900, 1655, 1630, 1545, 1460, 1400, 1255 cm^-1^; ^1^H and ^13^C NMR data, Table 2; HRESIMS *m/z* 1136.6924 [M+Na]^+^ (calc. for C_31_H_30_Br_6_N_4_O_11_Na, 1136.6849).

Suberein-2 (**3**): colourless amorphous solid (10 mg); [α]_D_^25^ +70.7 (*c* 1.0, MeOH); [α]_D_^25^ + 32.0 (*c* 0.2, acetone); UV (MeOH) λ_max_ (є) 208 (38200), 286 (10100) nm; ECD (0.15 mM, MeOH) λ_max_ (Δє) 215 (–1.73), 255 (2.33), 286 (2.19) nm; IR ν_max_ 3360, 2985, 2900, 1655, 1545, 1460, 1415, 1260 cm^-1^; ^1^H and ^13^C NMR data, Table 2; HRESIMS *m/z* 1136.6864 [M+Na]^+^ (calc. for C_31_H_30_Br_6_N_4_O_11_Na, 1136.6849).

Suberein-3 and suberein-4 (**4** and **5**): amorphous off-white powder (5 mg); UV (MeOH) λ_max_ (є) 210 (22500), 232 (7800), 260 (5100) nm; ^1^H and ^13^C NMR data, Table 3; HRESIMS *m/z* 1100.6935 [M+H]^+^ (calc. for C_30_H_29_Br_6_N_4_O_11_, 1100.6872)

Suberein-5 and Suberein-6 (**6** and **7**): amorphous off-white powder (5 mg); UV (MeOH) λ_max_ (є) 210 (22500), 232 (7800), 260 (5000) nm; ^1^H and ^13^C NMR data, Table 4; HRESIMS *m/z* 1100.6935 [M+H]^+^ (calc. for C_30_H_29_Br_6_N_4_O_11_, 1100.6872).

Suberein-7 (**8**): yellow amorphous solid (9 mg); [α]_D_^25^ +149.5 (*c* 0.5, MeOH); [α]_D_^25^ + 14.5 (*c* 0.2, acetone); UV (MeOH) λ_max_ (є) 210 (29500), 235 (15800), 283 (8800) nm; ECD (0.15 mM, MeOH) λ_max_ (Δє) 253 (7.5), 285 (8.0) nm; IR ν_max_ 3380, 2930, 1670, 1580, 1540, 1270 cm^-1^; ^1^H and ^13^C NMR data, Table 2; HRESIMS *m/z* 1110.6661 [M−H]^−^ (calc. for C_31_H_27_Br_6_N_4_O_11_, 1110.6715).

Suberein-8 (**9**): yellow amorphous solid (3 mg); [α]_D_^25^ +84.0 (*c* 0.2, MeOH); UV (MeOH) λ_max_ (є) 208 (27800), 283 (6700) nm; ECD (0.15 mM, MeOH) λ_max_ (Δє) 245 (7.2), 285 (7.8); IR ν_max_ 3350, 2930, 2855, 1665, 1600, 1540, 1460, 1400, 1380, 1250 cm^−1^; ^1^H and ^13^C NMR data, Table 5; ^1^H NMR (CD_3_OD, 500 MHz, 298 K): *δ* 4.09 (s, H-1), 6.41 (s, H-5), 3.75 (d, *J* = 18.0, H-7), 3.06 (d, *J* = 18.0, H-7), 3.74 (m, H-10), 3.40 (m, H-10), 4.76 (dd, *J* = 4.7; 7.2, H-11), 7.62 (s, H-13, H-13′), 4.07 (t, *J* = 6.5; 6.5, H-16), 2.11 (m, H-17), 2.87 (m, H-18), 2.47 (s, H-19), 2.47 (s, H-20), 3.73 (s, OMe); ^13^C NMR (CD_3_OD, 125 MHz, 298 K): *δ* 74.2 (C-1), 112.6 (C-2), 147.9 (C-3), 121.5 (C-4), 130.7 (C-5), 91.1 (C-6), 38.3 (C-7), 153.0 (C-8), 160.8 (C-9), 47.3 (C-10), 70.1 (C-11), 142.5 (C-12), 130.2 (C-13, C-13′), 117.6 (C-14, C14′), 152.1 (C-15), 70.8 (C-16), 26.5 (C-17), 56.0 (C-18), 43.8 (C-19), 43.8 (C-20), 59.0 (OMe); HRESIMS *m/z* 761.8695 [M+H]^+^ (calc. for C_23_H_28_Br_4_N_3_O_6_, 761.8671).

17-deoxy-11-*epi*-fistularin-3 (**10**): yellow amorphous solid (8 mg); [α]_D_^25^ +71.0 (*c* 0.3, MeOH); [α]_D_^25^ +20.0 (*c* 0.2, acetone); ECD (0,15 mM, MeOH)) λ_max_ (Δє) 251 (7.7), 286 (7.6); ^1^H NMR (acetone-*d_6_*, 500 MHz, 298 K): *δ* 7.74 (s, 1H, NH), 7.63 (s, 1H, NH’), 7.52 (s, 2H, H-15, H-15′), 6.52 (d, *J* = 11.0 Hz, 2H, H-5, H-5′), 5.45 (br s, 2H, OH-1, OH-1′), 4.47 (br s, 1H, OH-11), 4.24 (m, 1H, H-11), 4.18 (d, *J* = 13 Hz, 2H, H-1, H-1′), 4.03 (m, 2H, H-12), 3.86 (d, *J* = 18.6 Hz, 1H, H-7), 3.82 (d, *J* = 18.6 Hz, 1H, H-7′), 3.78 (m, 1H, H-10a), 3.73 (s, 6H, OCH3), 3.57 (dd, *J* = 7.0, 13 Hz, 2H, H-18), 3.52 (m, 1H, H-10b), 3.20 (d, *J* = 18.6 Hz, 1H, H-7), 3.15 (d, *J* = 18.6 Hz, 1H, H-7′), 2.88 (t, dd, *J* = 7.0, 13 Hz, H-17); HRESIMS *m/z* 1096.6923 [M−H]^−^ (calc. for C_31_H_29_Br_6_N_4_O_10_, 1096.6923).

General acid hydrolysis of compounds **1**, **2**, **3**, **8**, and **10** for the preparation of the 1-aminodiol for Marfey derivatization: the compounds (2 mg of each) were dissolved in a mixture of TFA/H_2_O (2/1, 2 mL) and heated in a closed tube at 120 °C for 36 h. Each solution was evaporated to dryness to obtain the corresponding crude hydrolysate.

##### Determination of C-11 Configuration for epi-fistularin-3 (**1**):

(a) Marfey derivatization: the hydrolysate was dissolved in water (100 μL) and combined with 1-fluoro-2,4-dinitrophenyl-5-L-alaninamide (L-FDAA) (1% w/v in acetone, 100 μL) and aqueous NaHCO_3_ (1.0 M, 40 μL). The solution was heated at 60 °C for 5 min, then cooled. The mixture was filtered on 0.2 μm Millex-LG. Standards *(R)* and *(S)*-3-amino-1,2-propanediol were converted to their L-FDAA derivatives following the same procedure. 

(b) LC analysis: the solutions of DAA derivatives were analyzed directly by UPLC/MS using HSS C18 column and eluted with H_2_O + 0.1% HCOOH/CH_3_CN+ 0.1% HCOOH (98/2 to 70/30 in 10 min). The *(R)-* and *(S)*-DAA derivatives of 3-amino-1,2-propanediol were eluted at R_T_ = 6.59 min and R_T_ = 6.68 min, respectively. Analysis of **1**-L-DAA derivative gave a retention time of R_T_ = 6.58 min, corresponding to an 11*R* configuration for epi-fistularin-3 (**1**).

##### Determination of C-11 Configuration for Compounds **2** and **3**.

(a) Marfey derivatization: the hydrolysate was dissolved in water (100 μL) and combined with 1-fluoro-2,4-dinitrophenyl-5-L-alaninamide (L-FDAA) (1% w/v in acetone, 100 μL) and aqueous NaHCO_3_ (1.0 M, 40 μL). The solution was heated at 60 °C for 5 min, then cooled. The mixture was filtered on 0.2 μm Millex-LG. Standards (*R*) and (*S*)-3-amino-1,2-propanediol were converted to their L-FDAA derivatives following the same procedure. 

(b) LC analysis: the solutions of DAA derivatives were analyzed directly by UPLC/MS using HSS C18 column and eluted with H_2_O + 0.1% HCOOH/CH_3_CN + 0.1% HCOOH (98/2 to 70/30 in 10 min). The (*R*)- and (*S*)-DAA derivatives of 3-amino-1,2-propanediol were eluted at R_T_ = 7.01 min and R_T_ = 7.10 min, respectively. Analysis of the compound L-DAA derivative gave a retention time of R_T_ = 7.01 min, corresponding to an 11*R* configuration for compounds **2** and **3**.

##### Determination of C-11 Configuration for Compound **8**

(a) Marfey derivatization: the hydrolysate was dissolved in water (100 μL) and combined with 1-fluoro-2,4-dinitrophenyl-5-L-alaninamide (L-FDAA) (1% w/v in acetone, 100 μL) and aqueous NaHCO3 (1.0 M, 40 μL). The solution was heated at 60 °C for 5 min, then cooled. The mixture was filtered on 0.2 μm Millex-LG. Standards (R) and (S)-3-amino-1,2-propanediol were converted to their L-FDAA derivatives following the same procedure. 

(b) LC analysis: the solutions of DAA derivatives were analyzed directly by UPLC/MS using HSS C18 column and eluted with H_2_O + 0.1% HCOOH/CH_3_CN+ 0.1% HCOOH (98/2 to 70/30 in 10 min). The (*R*)- and (*S*)-DAA derivatives of 3-amino-1,2-propanediol were eluted at R_T_ = 10.14 min and R_T_ = 10.30 min, respectively. Analysis of **8**-L-DAA derivative gave a retention time of R_T_ = 10.13 min, corresponding to an 11*R* configuration for compound **8**.

##### Determination of C-11 Configuration for 17-deoxy-epi-fistularin-3 (**10**)

(a) Marfey derivatization: the hydrolysate was dissolved in water (100 μL) and combined with 1-fluoro-2,4-dinitrophenyl-5-L-alaninamide (L-FDAA) (1% w/v in acetone, 100 μL) and aqueous NaHCO3 (1.0 M, 40 μL). The solution was heated at 60 °C for 5 min, then cooled. The mixture was filtered on 0.2 μm Millex-LG. Standards (R) and (S)-3-amino-1,2-propanediol were converted to their L-FDAA derivatives following the same procedure.

(b) LC analysis: the solutions of DAA derivatives were analyzed directly by UPLC/MS using HSS C18 column and eluted with H_2_O + 0.1% HCOOH/CH_3_CN + 0.1% HCOOH (98/2 to 70/30 in 10 min). The (*R*)- and (*S*)-DAA derivatives of 3-amino-1,2-propanediol were eluted at R_T_ = 6.78 min and R_T_ = 6.88 min, respectively. Analysis of **10**-L-DAA derivative gave a retention time of R_T_ = 6.80 min, corresponding to an 11*R* configuration for 17-deoxy-*epi*-fistularin-3 (**10**).

Conversion of 11-*epi*-fistularin-3 (**1**) to compounds **2**–**7** and agelorins A/B: compound **1** was dissolved in a mixture 1/1 of acetonitrile (+0.1% HCOOH)/water (+0.1% HCOOH) and stored at room temperature for two weeks. The reaction mixture was then dried under vacuum and submitted for HPLC analysis. Compound **1** gave agelorin A, agelorin B, and the compounds **2–7** by comparison with the retention time of each product.

### 4.4. Antibacterial Bioassay

Preparation of the compounds: antifouling efficiency of the compounds was assessed towards the inhibition of growth of key bacteria involved in marine and terrestrial surface colonisation. All bioassays were run in six replicates and using two batches of organisms. Compounds were tested at 0.01, 0.1, and 1 µg/mL. 96 well-plates were used and were coated with the compounds. Methanol was used as a carrier solvent and after its evaporation plates were sterilized under UV exposure for 30 min [41].

#### 4.4.1. Biological Material

Seven strains of marine bacteria i.e., *Halomonas aquamarina* (ATCC 14400), *Polaribacter irgensii* (ATCC 700398), *Shewanella putrefaciens* (ATCC 8071), *Roseobacter littoralis* (ATCC 495666), *Pseudoalteromonas elyakovii* (ATCC 700519), *Vibrio aestuarianus* (ATCC 35048), *Vibrio natriegens* (ATCC 14058), and three strains of terrestrial bacteria, i.e., *Escherichia coli* (ATCC 11775), *Staphylococcus aureus* (ATCC 126000), and *Pseudomonas aeruginosa* (ATCC 16145) were used to assess the antibacterial potency of the compounds.

For maintenance, bacterial strains were kept on solid media: autoclaved seawater, 0.5% peptone (oxoid), and 1% agar for marine strains [51].

#### 4.4.2. Bioassay

Inhibition of bacterial growth: experiments were run as previously described by Maréchal et al. [52]. 96-well plates containing the compounds were inoculated with the bacteria (2 × 10^8^ cells/mL) in LB medium (Luria Hinton Broth, Sigma, Andover, UK), (supplemented with NaCl (35 g/L) for marine strains), at 30 °C for 48 h. After incubation, the intensity of growth in the presence of the tested compounds and control (media only) was assessed by measurement of the OD 620 nm using a spectrophotometer (Apollo LB912, Berthold Technologies). Results are expressed as minimum inhibitory concentrations (MIC values).

### 4.5. Acetylcholinesterase Inhibition Bioassay

This bioassay was set up after Ellman [53]. This method is the most commonly used to screen compounds against this target. All reagents, buffers, and salts were purchased from Sigma-Aldrich (Saint-Louis, MO, USA). Two buffer solutions were prepared extemporaneously: buffer A (50 mM Tris-HCl pH 8 + 0.1% BSA); and buffer B (50 mM Tris-HCl pH 8 + 0.1 M NaCl + 0.02 M MgCl_2_.6H_2_O). Acetylcholinesterase from *Electrophorus electricus* (EC 3.1.1.7, AChE−0.2 U/mL) was dissolved in buffer A, 5,5′-dithiobis-2-nitrobenzoic acid (DTNB-Ellman’s reagent−3 mM) in buffer B, and acetylthiocholine iodide (ACTI−6 mM) in H_2_O. Tested compounds were dissolved in DMSO at 10 mg/mL, stored at −20 °C, and subsequently 100 times diluted in H_2_O before the assay. 10 μL AChE was preincubated for 10 min with 70 μL DTNB and 10 μL compounds solution in 96-well microplates. The enzymatic reaction was initiated with the addition of 10 μL ACTI, a synthetic substrate of AChE. After 30 min at 25 °C, absorbance of the plate was measured at 405 nm using a microplate reader (Tecan Infinite M200). Percentage of inhibition was calculated using the following equation: 100 × (A_control_ − A_sample_)/(A_control_ − A_0_), where A_control_ is the average absorbance of the wells in which tested compounds were replaced by water, A_sample_ is the absorbance of the well with tested compound, and A_0_ the average absorbance of blank wells where AChE was omitted. Products leading to 50% inhibition of AChE were then submitted to dose-response experiments. Serial dilutions of the products were prepared (100, 50, 25, 12.5, 6.25, 3.125, 1.5625, and 0.78125 µg/mL). Enzymatic assay was performed according to the same procedure with 10 µL of each concentration added. IC_50_ values (concentration leading to 50% inhibition of enzymatic activity) were graphically determined by plotting the percentage of inhibition against the compound concentrations.

Each assay was performed in triplicate and results are expressed as mean values ± SD.

### 4.6. DNMT1 Activity Inhibition Bioassay

DNMT1 activity was measured using in vitro DNMT activity/inhibition assay (Active Motif, Rixensart, Belgium) according to manufacturer’s instructions. All compounds were used at a final 50 µM concentration. The methylation reaction was performed by incubating 25 ng of purified DNMT1 with compounds for 2 h at 37 °C in the presence of 0.01% Triton X-100. The methylated DNA was then recognized by the His-tagged methyl-CpG binding domain protein 2b. The addition of a poly-histidine antibody conjugated to horseradish peroxidase provided a colorimetric readout quantified with a spectrophotometer (SpectraCount, Packard) at the wavelength of 450 nm. Data of DNMT1 activity are reported as percentage of control.

## Figures and Tables

**Table 1:** Δ*δ* (= *δ*_S_−*δ*_R_) (at 300 MHz) data for the *S*- and *R*-MTPA-*epi*-fistularin-3 esters.

Position	*δS*-Ester (ppm)	*δR*-Ester (ppm)	Δ*δ^SR^* (ppm)	Δ*δ^SR^* (Hz)
15, 15′	7.63	7.73	−0.10	−30
17	6.13	6.21	−0.08	−24
18	3.98	3.80	+0.18	+54
NH’	7.95	7.68	+0.27	+81

**Table 2:** NMR spectroscopic data of suberein-1 (**2**) and suberein-2 (**3**) in acetone-*d_6_.*

	Suberein-1 (2) (500 MHz)		Suberein-2 (3) (600 MHz)	
Position	δ_C_, Type	δ_H_ Mult*,* (*J* in Hz)	δ_C_, Type	δ_H_ Mult*,* (*J* in Hz)
1	75.2, CH	4.20, s	155.1, C	-
2	113.9, C	-	108.9, C	-
3	148.8, C	-	154.7, C	-
4	122.1, C	-	106.1, C	-
5	132.1, CH	6.53, s	134.7, CH	7.57, s
6	91.9, C	-	122.2, C	-
7	40.1, CH_2_	3.85, d (18.4) 3.19, d (18.4)	25.6, CH_2_	3.84, s
8	155.2, C	-	151.1, C	-
9	160.4, C	-	166.6, C	-
10	43.5, CH_2_	3.78, m 3.52, m	43.6, CH_2_	3.78, m 3.58, m
11	69.8, CH	4.24, m	69.2, CH	4.25, m
12	75.9, CH_2_	4.05, dd (9.0, 5.2) 4.02, dd (9.0, 5.2)	75.6, CH_2_	4.04, dd (9.1, 5.4) 3.99, dd (9.1, 5.4)
13	152.7, C	-	152.5, C	-
14, 14′	118.4, C	-	118.3, C	-
15, 15′	131.5, CH	7.65, s	131.4, CH	7.63, s
16	143.1, C	-	143.2, C	-
17	71.0, CH	4.92, dd (7.7, 4.5)	71.3, CH	4.89, dd (7.7, 4.5)
18	47.7, CH_2_	3.64, m 3.49, m	47.4, CH_2_	3.61, m 3.45, m
1′	155.1, C *	-	75.1, CH	4.18, s
2′	108.9, C	-	113.9, C	-
3′	154.8, C	-	148.7, C	-
4′	106.3, C	-	122.1, C	-
5′	134.9, CH	7.56, s	132.1, CH	6.52, s
6′	122.1, C	-	91.8, C	-
7′	25.7, CH_2_	3.81, s	39.8, CH_2_	3.83, d (18.4) 3.17, d (18.4)
8′	151.2, C	-	155.3, C	-
9′	166.7, C	-	160.4, C	-
OMe	60.2, CH_3_	3.73, s	60.5, CH_3_	3.80, s
OMe’	60.6, CH_3_	3.81, s	60.5, CH_3_	3.72, s
NH	-	7.62, bt (5.7)	-	8.10, bt (6.8)
NH’	-	8.05, bt (6.8)	-	7.74, bt (5.7)
OH-1, OH-1′	-	-	-	-
OH-11	-	-	-	-
OH-17	-	-	-	-

**Table 3:** NMR spectroscopic data of suberein-3 (**4**) and suberein-4 (**5**) (500, 125 MHz, acetone-*d_6_*).

	Suberein-3 (4)		Suberein-4 (5)	
Position	δ_C_, Type	δ_H_ Mult*,* (*J* in Hz)	δ_C_, Type	δ_H_ Mult*,* (*J* in Hz)
1	75.0, CH	4.18, d (6.0)	75.2, CH	4.40, dd (11.4, 6.0)
2	113.9, C	-	57.4, CH	5.09, d (11.4)
3	148.8, C	-	183.6, C	-
4	122.2, C	-	122.6, C	-
5	132.3, CH	6.53, s	149.3, CH	7.62, s
6	91.8, C	-	91.8, C	-
7	40.0, CH_2_	3.82, d (18.4) 3.16, d (18.4)	38.4, CH_2_	3.86, d (18.4) 3.25, d (18.4)
8	155.2, C	-	155.1, C	-
9	160.3, C	-	160.4, C	-
10	43.7, CH_2_	3.78, m 3.52, m	43.7, CH_2_	3.78, m 3.52, m
11	69.9, CH	4.25, m	69.9, CH	4.25, m
12	76.0, CH_2_	4.06, m 4.04, m	76.0, CH_2_	4.06, m 4.04, m
13	152.7, C	-	152.2, C	-
14, 14′	118.4, C	-	118.3, C	-
15, 15′	131.5, CH	7.66, s	131.5, CH	7.67, s
16	143.3, C	-	143.3, C	-
17	71.4, CH	4.91, m	71.4, CH	4.91, m
18	47.6, CH_2_	3.62, m 3.48, m	47.6, CH_2_	3.62, m 3.48, m
1′	75.3 CH	4.41, dd (11.4, 6.0)	75.1, CH	4.20, d (6.0)
2′	57.4, CH	5.08, d (11.4)	113.9, C	-
3′	183.6, C	-	148.7, C	-
4′	122.6, C	-	122.2, C	-
5′	149.4, CH	7.64, s	132.1, CH	6.52, s
6′	91.8, C	-	91.8, C	-
7′	38.5, CH_2_	3.88, d (18.4) 3.29, d (18.4)	40.1, CH_2_	3.86, d (18.4) 3.20, d (18.4)
8′	155.1, C	-	155.2, C	-
9′	160.4, C	-	160.3, C	-
OMe	60.2, CH_3_	3.73, s	-	-
OMe’	-	-	60.2, CH_3_	3.73, s
NH	-	7.63, m	-	7.63, m
NH’	-	7.67, m	-	7.67, m
OH-1	-	5.41, d (6.0)	-	5.96, d (6.0)
OH-1′	-	5.97, d (6.0)	-	5.41, d (6.0)
OH-11	-	4.44, m	-	4.44, m
OH-17	-	5.01, m	-	5.01, m

**Table 4:** NMR spectroscopic data of suberein-5 (**6**) and suberein-6 (**7**) (500, 125 MHz, acetone-*d*_6_).

	Suberein-5 (6)		Suberein-6 (7)	
Position	δ_C_, Type	δ_H_ Mult*,* (*J* in Hz)	δ_C_, Type	δ_H_ Mult*,* (*J* in Hz)
1	75.2, CH	4.18, br s	73.1, CH	4.57, br s
2	113.9, C	-	54.8, CH	5.27, br s
3	148.8, C	-	183.6, C	-
4	122.2, C	-	122.1, C	-
5	132.4, CH	6.53, s	146.4, CH	7.47, s
6	91.8, C	-	90.8, C	-
7	40.1, CH_2_	3.82, d (18.4) 3.16, d (18.4)	41.5, CH_2_	3.88, d (18.4) 3.35, d (18.4)
8	155.2, C	-	155.1, C	-
9	160.4, C	-	160.5, C	-
10	43.6, CH_2_	3.78, m 3.52, m	43.6, CH_2_	3.78, m 3.52, m
11	69.9, CH	4.25, m	69.9, CH	4.25, m
12	76.0, CH_2_	4.06, m 4.04, m	76.0, CH_2_	4.06, m 4.04, m
13	152.7, C	-	152.7, C	-
14, 14′	118.4, C	-	118.4, C	-
15, 15′	131.4, CH	7.67, s	131.3, CH	7.66, s
16	143.3, C	-	143.3, C	-
17	71.4, CH	4.91, m	71.4, CH	4.91, m
18	47.6, CH_2_	3.62, m 3.48, m	47.6, CH_2_	3.62, m 3.48, m
1′	73.2 CH	4.57, br s	75.3, CH	4.19, br s
2′	54.8, CH	5.27, br s	113.9, C	-
3′	183.6, C	-	148.8, C	-
4′	122.1, C	-	122.2, C	-
5′	146.4, CH	7.49, s	132.3, CH	6.52, s
6′	90.8, C	-	91.8, C	-
7′	41.5, CH_2_	3.91, d (18.4) 3.39, d (18.4)	40.1, CH_2_	3.86, d (18.4) 3.20, d (18.4)
8′	155.1, C	-	155.2, C	-
9′	160.5, C	-	160.4, C	-
OMe	60.2, CH_3_	3.73, s	-	-
OMe’	-	-	60.2, CH_3_	3.73, s
NH	-	7.63, br t (5.0)	-	7.66, br t (5.0)
NH’	-	7.66, br t (5.0)	-	7.73, br t (5.0)
OH-1	-	5.45, br s	-	5.95, br s
OH-1′	-	5.95, br s	-	5.45, br s
OH-11	-	4.47, br s	-	4.47, br s
OH-17	-	5.04, br s	-	5.04, br s

**Table 5:** Spectroscopic data of compounds **8** (600 MHz, acetone-*d_6_*) and **9** (500, MeOH-*d*_4_).

Suberein-7 (8) (600 MHz, Acetone-*d_6_*)			Suberein-8 (9) (500, MHz, MeOH-*d*_4_)		
Position	δ_C_, Type	δ_H_ Mult*,* (*J* in Hz)	Position	δ_C_, Type	δ_H_ Mult*,* (*J* in Hz)
1	75.2, CH	4.19, s	1	74.2, CH	4.09, s
2	113.9, C	-	2	112.6, C	-
3	148.7, C	-	3	147.9, C	-
4	122.1, C	-	4	121.5, C	-
5	132.3, CH	6.53, s	5	130.7, CH	6.41, s
6	91.8, C	-	6	91.1, C	-
7	40.1, CH_2_	3.86, d (18.4) 3.22, d (18.4)	7	38.3, CH_2_	3.75, d (18.0) 3.06, d (18.0)
8	154.8, C	-	8	153.0, C	-
9	160.6, C	-	9	160.8, C	-
10	43.5, CH_2_	3.78, m 3.53, m	10	47.3, CH_2_	3.74, m 3.40, m
11	69.9, CH	4.29, m	11	70.1, CH_2_	4.76, dd (7.2, 4.7)
12	76.3, CH_2_	4.16, m	12	142.5, C	-
13	158.0, C	-	13, 13′	130.2, CH	7.62, s
14, 14′	119.2, C	-	14, 14′	117.6, C	-
15, 15′	133.6, CH	8.28, s	15	152.1, C	-
16	134.3, C	-	16	71.8, CH_2_	4.07, t (6.5)
17	192.2, C	-	17	26.5, CH_2_	2.11, m
18	45.9, CH_2_	4.87, d (5.2)	18	56.0, CH_2_	2.87, m
1′	75.2, CH	4.23, s	19	43.8 CH_3_	2.47, s
2′	113.9, C	-	20	43.8, CH	2.47, s
3′	148.7, C	-	OMe	59.0, CH_3_	3.73, s
4′	122.1, C	-			
5′	132.3, CH	6.57, s			
6′	92.0, C	-			
7′	39.9, CH_2_	3.86, d (18.4) 3.19, d (18.4)			
8′	155.2, C	-			
9′	160.2, C	-			
OMe	60.2, CH_3_	3.73, s			
OMe’	60.2, CH_3_	3.73, s			
NH	-	7.67, bt (6.2)			
NH’	-	7.91, bt (5.2)			
OH-1, OH-1′	-	5.47, br s			
OH-11	-	4.58, br s			
OH-17	-	-			

**Table 6:** Biological activity of *epi*-fistularin-3 (**1**) and related compounds.

	*Vibrio* *aesturianus*	*Roseobacter littoralis*	*Halomonas* *aquamarina*	*Escherichia coli*	Acetylcholinesterase
Compounds	MIC µM	MIC µM	MIC µM	MIC µM	IC_50_ µM
*epi*-fistularin-3 (**1**)	>1	>1	>1	>1	>10
suberein-1 (**2**)	0.01	1	>1	>1	>10
suberein-2 (**3**)	0.01	>1	>1	0.01	>10
17-oxo-11-*epi*-fistularin-3 (**8**)	0.01	>1	>1	>1	>10
17-deoxy-11-*epi*-fistularin-3 (**10**)	>1	>1	0,01	>1	>10
agelorin A (**11**)	0.1	0.1	0.1	nt	0.19 ± 0.2
agelorin B (**12**)	>1	>1	>1	>1	nt
11-deoxyfistularin-3 (**13**)	>1	>1	0,01	>1	>10
11,17-dideoxyfistularin (**14**)	>1	>1	0,01	>1	10 ± 0.3
11-hydroxyaerothionin (**15**)	>1	>1	0,01	>1	10 ± 0.3

## Supplementary Materials

The following are available online at https://www.mdpi.com/1660-3397/19/3/143/s1: Spectroscopic data (HRMS, ^1^H NMR, ^13^C NMR, COSY, HSQC, ECD, and HMBC spectra) for compounds **1**–**10**.
