# Supplementary material for: Bioactive Bromotyrosine Derivatives from the Pacific Marine Sponge Suberea clavata (Pulitzer-Finali, 1982)
Source: Mar Drugs. 2021 Mar 6;19(3):143. doi: 10.3390/md19030143 (PMC7999702; doi:10.3390/md19030143)

Supporting information

Bioactive Bromotyrosine Derivatives from the Pacific Marine Sponge *Suberea clavata* (Pulitzer-Finali, 1982)

Céline Moriou [1], Damien Lacroix [1], Sylvain Petek [2,*], Amr El-Demerdash [1], Rozenn Trepos [2], Tinihauarii Mareva Leu [3], Cristina Florean [4], Marc Diederich [5], Claire Hellio [2], Cécile Debitus [2], and Ali Al-Mourabit [1,*]

[1] Université Paris-Saclay, CNRS, Institut de Chimie des Substances Naturelles, Gif-sur-Yvette, 91190, France; crespipharma@gmail.com (D.L.) ; celine.moriou@cnrs.fr (C.M.) ; eldemerdash555@gmail.com (A.E.-D.)

[2] IRD, Univ Brest, CNRS, Ifremer, LEMAR, F-29280 Plouzane, France; cecile.debitus@ird.fr (C.D.) ; claire.hellio@univ-brest.fr (C.H.) ; rozenn.trepos@gmail.com (R.T.);

[3] Univ de la Polynésie française, IRD, Ifremer, ILM, EIO, F-98713 Papeete, French Polynesia; mareva.leu@gmail.com (T.M.L.)

[4] Laboratoire de Biologie Moléculaire et Cellulaire du Cancer, Hôpital Kirchberg, 9, rue Edward Steichen, L-2540 Luxembourg, Luxembourg; cristina.florean@lbmcc.lu (C.F.),

[5] Department of Pharmacy, Research Institute of Pharmaceutical Sciences, College of Pharmacy, Seoul National University, 1 Gwanak-ro, Gwanak-gu, Seoul 08826, Korea; marcdiederich@snu.ac.kr (M.D.)

* Correspondence: sylvain.petek@ird.fr (S.P.), Tel.: +33-298-498-651; ali.almourabit@cnrs.fr (A. A.-M.), Tel.: +33-169-824-585

Table of Contents

Figure S0: Known isolated natural products **1** and **11**-**23**. 4
Identification of the known compounds **1** and **11**-**20**. 4
Figure S1: ^{1}H NMR spectrum of 17*S*-*epi*-fistularin-3 (**1**) in acetone-d_6 (500 MHz) 7
Figure S2: ^{13}C NMR spectrum of 17*S*-*epi*-fistularin-3 (**1**) in acetone-d_6 (500 MHz) 8
Figure S3: ^{13}C NMR spectrum of 17*S*-*epi*-fistularin-3 (**1**) in acetone-d_6 (500 MHz), Zoom on C-11 and C-17. 9
Figure S4: ECD spectrum of 17*S*-*epi*-fistularin-3 (**1**) in MeOH (c 0.15 mM) 10
Figure S5: ^{1}H NMR spectrum of the (*S*)-MPTA ester of **1** in acetone-d_6 (500 MHz) 11
Figure S6: ^{1}H NMR spectrum of the (*R*)-MPTA ester of **1** in acetone-d_6 (500 MHz). 12
Figure S7: HPLC Chromatogram (UV) of Conversion of 11-epi-fistularin-3 (**1**) to compounds **2** – **7** and agelorins A/B 13
Figure S8: ^{1}H NMR spectrum of suberein-1 (**2**) in acetone-d_6 (500 MHz). 14
Figure S9: ^{13}C NMR spectrum of suberein-1 (**2**) in acetone-d_6 (125 MHz). 15
Figure S10: ^{1}H-^{1}H COSY NMR spectrum of suberein-1 (**2**) in acetone-d_6 (500 MHz). 16
Figure S11: ^{1}H-^{13}C HSQC NMR spectrum of suberein-1 (**2**) in acetone-d_6 (500 MHz). 17
Figure S12: ^{1}H-^{13}C HMBC NMR spectrum of suberein-1 (**2**) in acetone-d_6 (500 MHz). 18
Figure S13: HR-ESI mass spectrum of suberein-1 (**2**). 19
Figure S14: ECD spectrum of suberein-1 (**2**) in MeOH (*c* 0.15 mM) 19
Figure S15: ^{1}H NMR spectrum of suberein-2 (**3**) in acetone-d_6 (600 MHz) 20
Figure S16: ^{13}C NMR spectrum of suberein-2 (**3**) in acetone-d_6 (150 MHz). 21
Figure S17: ^{1}H-^{1}H COSY NMR spectrum of suberein-2 (**3**) in acetone-d_6 (600 MHz). 22
Figure S18: ^{1}H-^{13}C HSQC NMR spectrum of suberein-2 (**3**) in acetone-d_6 (600 MHz). 23
Figure S19: ^{1}H-^{13}C HMBC NMR spectrum of suberein-2 (**3**) in acetone-d_6 (600 MHz). 24
Figure S20: HRESMS-MS fragmentation spectra of suberein-2 (**3**) ($[M+Na]^+$ and $[M+H]^+$ respectively) 25
Figure S21: ECD spectrum of suberein-2 (**3**) in MeOH (c 0.15 mM) 26
Figure S22: ^{1}H NMR spectrum of the mixture of suberein-3 (**4**) and suberein-4 (**5**) in acetone-d_6 (500 MHz). 27
Figure S23: ^{13}C NMR spectrum of the mixture of suberein-3 (**4**) and suberein-4 (**5**) in acetone-d_6 (125 MHz). 28
Figure S24: ^{1}H-^{1}H COSY NMR spectrum of the mixture of suberein-3 (**4**) and suberein-4 (**5**) in acetone-d_6 (500 MHz). 29
Figure S25: ^{1}H-^{13}C HSQC NMR spectrum of the mixture of suberein-3 (**4**) and suberein-4 (**5**) in acetone-d_6 (500 MHz). 30

Figure S26: ^{1}H-^{13}C HMBC NMR spectrum of the mixture of suberein-3 (**4**) and suberein-4 (**5**) in acetone-d_6 (500 MHz). 31
Figure S27: ^{1}H-^{1}H NOESY NMR spectrum of the mixture of suberein-3 (**4**) and suberein-4 (**5**) in acetone-d_6 (500 MHz). 32
Figure S28: HR-ESI mass spectrum of the mixture of suberein-3 (**4**) and suberein-4 (**5**). 33
Figure S29: ^{1}H NMR spectrum of the mixture of suberein-5 (**6**) and suberein-6 (**7**) in acetone-d_6 (500 MHz). 34
Figure S30: ^{13}C NMR spectrum of the mixture of suberein-5 (**6**) and suberein-6 (**7**) in acetone-d_6 (125 MHz). 35
Figure S31: ^{1}H-^{1}H COSY NMR spectrum of the mixture of suberein-5 (**6**) and suberein-6 (**7**) in acetone-d_6 (500 MHz). 36
Figure S32: ^{1}H-^{13}C HSQC NMR spectrum of the mixture of suberein-5 (**6**) and suberein-6 (**7**) in acetone-d_6 (500 MHz). 37
Figure S33: ^{1}H-^{13}C HMBC NMR spectrum of the mixture of suberein-5 (**6**) and suberein-6 (**7**) in acetone-d_6 (500 MHz). 38
Figure S34: ^{1}H-^{1}H NOESY NMR spectrum of the mixture of suberein-5 (**6**) and suberein-6 (**7**) in acetone-d_6 (500 MHz). 39
Figure S35: HR-ESI mass spectrum of the mixture of suberein-5 (**6**) and suberein-6 (**7**). 40
Figure S36: ^{1}H NMR spectrum of suberein-7 (**8**) in acetone-d_6 (500 MHz) 41
Figure S37: ^{13}C NMR spectrum of suberein-7 (**8**) in acetone-d_6 (125 MHz). 42
Figure S38: ^{1}H-^{1}H COSY NMR spectrum of suberein-7 (**8**) in acetone-d_6 (500 MHz). 43
Figure S39: ^{1}H-^{13}C HSQC NMR spectrum of suberein-7 (**8**) in acetone-d_6 (500 MHz). 44
Figure S40: ^{1}H-^{13}C HMBC NMR spectrum of suberein-7 (**8**) in acetone-d_6 (500 MHz). 45
Figure S41: HR-ESI mass spectrum of suberein-7 (**8**). 46
Figure S42: ECD spectrum of suberein-7 (**8**) in MeOH (c 0.15 mM) 46
Figure S43: ^{1}H NMR spectrum of suberein-8 (**9**) in acetone-d_6 (500 MHz) 47
Figure S44: ^{13}C NMR spectrum of suberein-8 (**9**) in acetone-d_6 (125 MHz). 48
Figure S45: ^{1}H-^{1}H COSY NMR spectrum of suberein-8 (**9**) in acetone-d_6 (500 MHz). 49
Figure S46: ^{1}H-^{13}C HSQC NMR spectrum of suberein-8 (**9**) in acetone-d_6 (500 MHz). 50
Figure S47: ^{1}H-^{13}C HMBC NMR spectrum of suberein-8 (**9**) in acetone-d_6 (500 MHz). 51
Figure S48: HR-ESI mass spectrum of suberein-8 (**9**). 52
Figure S49: ^{1}H NMR spectrum of 17-deoxy-*epi*-fistularin-3 (**10**) in acetone-d_6 (500 MHz). 53
Figure S50: HR-ESI mass spectrum of 17-deoxy-*epi*-fistularin-3 (**10**). 54
Figure S51: ECD spectrum of 17-deoxy-*epi*-fistularin-3 (**10**) in MeOH (c 0.15 mM). 54

Figure S0: Known isolated natural products **1** and **11-23**.

Known bromotyrosins from the Sponge *Suberea clavata* studied in this paper

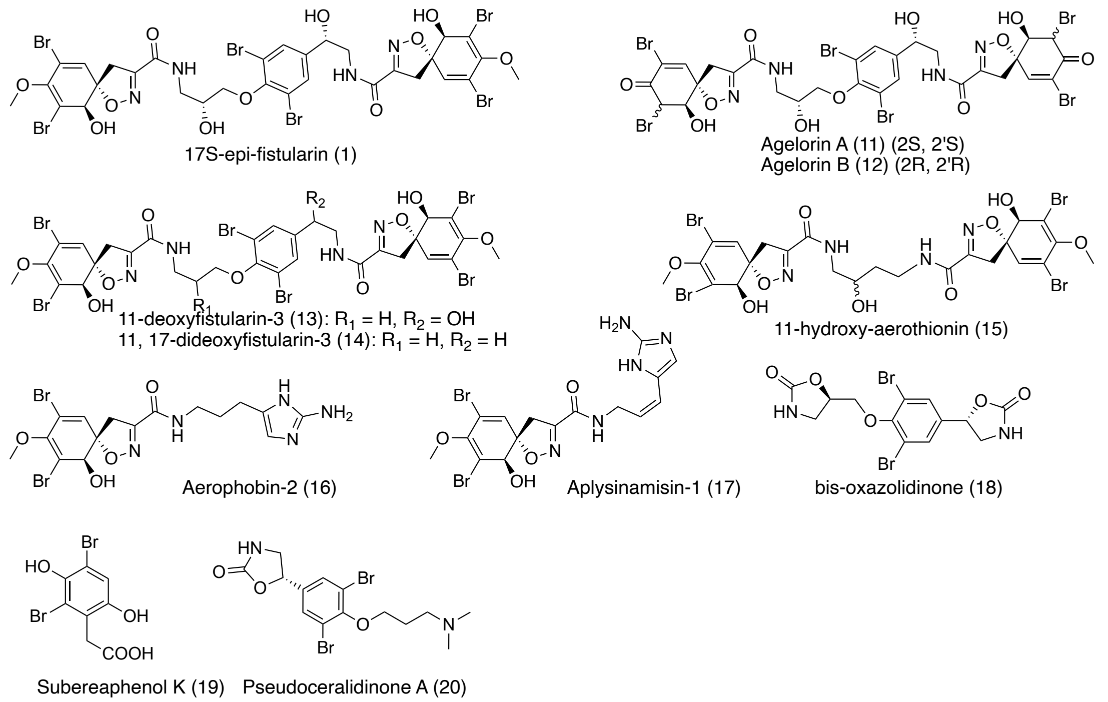

Known bromotyrosins previously isolated from another sponge species, *Suberea ianthelliformis*

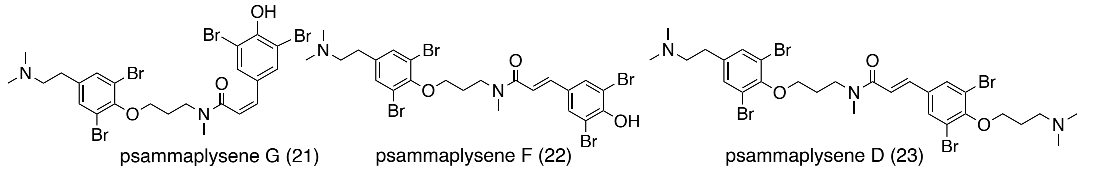

Identification of the known compounds 1 and 11-20.

17S-epi-fistularin-3 (**1**): yellowish amorphous solid (3 g); $[\alpha]_D^{25}$ +148.0 (*c* 1.0, MeOH); $[\alpha]_D^{25}$ +169.0 (*c* 0.2, Acetone); ECD (0.15 mM, MeOH) λ_{max} ($\Delta\epsilon$) 253 (+7.5), 285 (+8.0); ^{1}H NMR (500 MHz, acetone-d_6): δ ppm 7.67 (m, 1H, NH′), 7.66 (s, 2H, H-15, H-15′), 7.62 (m, 1H, NH), 6.53 (d, 2H, H-5, H-5'), 5.41 (d, *J* = 8.0 Hz, 2H, OH-1, OH-1′), 5.00 (d, *J* = 4.3 Hz, 1H, OH-17), 4.90 (ddd, *J* = 7.7, 5.5, 4.3 Hz, 1H, H-17), 4.44 (d, *J* = 5.3 Hz, 1H, OH-11), 4.25 (m, 1H, H-11), 4.18 (dd, *J* = 8.0 Hz, 2H, H-1, H-1′), 4.04 (m, 2H, H-12), 3.85 (d, *J* = 18.0 Hz, 1H, H-7a), 3.82 (d, *J* = 18.0 Hz, 1H, H-7a′), 3.80 (m, 1H, H-10a), 3.73 (s, 6H, OCH_3, OCH_3′), 3.63 (m, 1H, H-18a), 3.54 (m, 1H, H-10b), 3.49 (m, 1H, H-18b), 3.19 (d, *J* = 18.0 Hz, 1H, H-7b), 3.16 (d, *J* = 18.0 Hz, 1H, H-7b′); ^{13}C NMR (125 MHz, acetone-d_6): δ ppm 160.5 (C-9, C-9′), 155.2 (C-8′), 155.1 (C-8), 152.7 (C-13), 148.8 (C-3, C-3′), 143.3 (C-16), 132.4 (C-5′), 132.3 (C-5), 131.5 (C-15, C-15′), 122.1 (C-4, C-4′), 118.4 (C-

14, C14′), 113.9 (C-2′), 113.8 (C-2), 91.8 (C-6, C-6′), 75.9 (C-12), 75.2 (C-1, C-1′), 71.0 (C-7), 69.9 (C-11), 60.2 (OCH_3, OCH_3′), 47.7 (C-18), 43.6 (C-10), 40.0 (C-7, C-7′); HRESIMS *m/z* 1136.6934 $[M+Na]^+$ (calc. for $C_{31}H_{29}{}^{79}Br_3{}^{81}Br_3N_4O_{11}Na$, 1136.6847).

Agelorin A (**11**): amorphous solid (3 mg); $[\alpha]_D^{25}$ -10.5 (*c* 0.2, Acetone); ^{1}H NMR (500 MHz, acetone-*d*$_6$): δ ppm 7.68 (s, 4H, H-15, H-15′, NH, NH′), 7.64 (s, 1H, H-5), 7.61 (, 1H, H-5′), 5.99 (br s, 2H, OH-1, OH-1′), 5.08 (d, *J* = 11 Hz, 1H, H-2), 5.07 (d, *J* = 11 Hz, 1H, H-2′), 4.91 (br s, 1H, H-17), 4.40 (2H, H-1, H-1′), 4.26 (m, 2H, H-11), 4.06 (m, 2H, H-12), 3.88 (d, *J* = 18 Hz, 1H, H-7a), 3.86 (d, *J* = 18 Hz, 1H, H-7′a), 3.80 (m, 1H, H-10a), 3.63 (m, 1H, H-18a), 3.50 (m, 2H, H-10b, H-18b), 3.30 (d, *J* = 18 Hz, 1H, H-7b), 3.26 (d, *J* = 18 Hz, 1H, H-7′b); HRESIMS *m/z* 1108.6653 $[M+H]^+$ (calc. for $C_{31}H_{27}{}^{79}Br_3{}^{81}Br_3N_4O_{11}$, 1108.6534).

Agelorin B (**13**): amorphous solid (3 g); ^{1}H NMR (500 MHz, acetone-*d*$_6$): δ ppm 7.71 (br s, 4H, H-15, H-15′, NH, NH′), 7.61 (d, *J* = 6 Hz, 1H, H-5), 7.41 (d, *J* = 6 Hz, 1H, H-5′), 5.29 (br s, 2H, H-2, H-2′), 4.89 (m, 1H, H-17), 4.49 (2H, H-1, H-1′), 4.26 (m, 2H, H-11), 4.12 (m, 2H, H-12), 3.81 (d, *J* = 18 Hz, 1H, H-7a), 3.78 (d, *J* = 18 Hz, 1H, H-7′a), 3.81 (m, 1H, H-10a), 3.61 (m, 1H, H-18a), 3.53 (m, 2H, H-10b, H-18b), 3.26 (d, *J* = 18 Hz, 1H, H-7b), 3.22 (d, *J* = 18 Hz, 1H, H-7′b); HRESIMS *m/z* 1108.6653 $[M+H]^+$ (calc. for $C_{31}H_{27}{}^{79}Br_3{}^{81}Br_3N_4O_{11}$, 1108.6534).

11-deoxyfistularin-3 (**13**): brown amorphous solid (36 mg); $[\alpha]_D^{25}$ +86.0 (*c* 1.0, MeOH); $[\alpha]_D^{25}$ +115.5 (*c* 0.2, Acetone); ^{1}H NMR (500 MHz, acetone-*d*$_6$): δ ppm 7.70 (m, 2H, NH, NH′), 7.67 (s, 2H, H-15, H-15′), 6.52 (d, *J* = 2.5 Hz, 2H, H-5, H-5′), 5.44 (dd, *J* = 8.5, 17.0 Hz, 2H, OH-1, OH-1′), 5.03 (s, 1H, OH-17), 4.90 (m, 1H, H-17), 4.17 (d, *J* = 8 Hz, 2H, H-1, H-1′), 4.10 (dd, *J* = 6.0 Hz, 2H, H-12), 3.85 (d, *J* = 10 Hz, 1H, H-7a), 3.81 (d, *J* = 10 Hz, 1H, H-7′a), 3.72 (s, 6H, O-CH_3), 3.62 (m, 3H, H-10a, H-18a), 3.47 (m, 1H, H-18b), 3.30 (m, 1H, H-10b), 3.20 (d, *J* = 11 Hz, 1H, H-7b), 3.16 (d, *J* = 11 Hz, 1H, H-7′b), 2.14 (m, 2H, H-11); HRESIMS *m/z* 1120.6935 $[M+Na]^+$ (calc. for $C_{31}H_{30}{}^{79}Br_3{}^{81}Br_3N_4O_{10}Na$, 1120.6898).

11,17-dideoxyfistularin-3 (**14**): yellowish amorphous solid (200 mg); $[\alpha]_D^{25}$ +152.5 (*c* 0.2, Acetone); ECD (0.15 mM, MeOH) λ_{max} ($\Delta\epsilon$) 256 (+7.5), 283 (+8.1); ^{1}H NMR (500 MHz, CD_3OD): δ ppm 7.47 (s, 2H, H-15), 6.42 (d, *J* = 1 Hz, 1H, H-5), 6.41 (d, *J* = 1 Hz, 1H, H-5′), 4.09 (s, 2H, H-1, H-1′), 4.06 (t, *J* = 6, 12 Hz, 2H, H-12), 3.78 (d, *J* = 18 Hz, 1H, H-7a), 3.75 (d, *J* = 18 Hz, 1H, H-7′a), 3.73 (s, 6H, OCH_3, OCH_3′), 3.59 (t, *J* = 7, 14 Hz, 2H, H-10), 3.47 (t, *J* = 7, 14 Hz, 2H, H-18), 3.11 (d, *J* = 18 Hz, 1H, H-7b), 3.05 (d, *J* = 18 Hz, 1H, H-7′b), 2.81 (t *J* = 18 Hz, 2H, H-17), 2.13 (m, 2H, H-11); ^{13}C NMR (125 MHz, CD_3OD): δ ppm 161.7 (C-9, C-9′), 155.5–155.3 (C-8, C-8′), 153.0 (C-13), 149.5 (C-3, C-3′), 139.8 (C-16), 134.6 (C-15, C-15′), 132.3 (C-5, C-5′), 122.9 (C-4, C-4′), 119.1 (C-14, C14′), 114.3 (C-2, C-2′), 92.6 (C-6, C-6′), 75.6 (C-1, C-1′), 72.3 (C-12), 60.5 (OCH_3, OCH_3′), 41.6 (C-18), 40.3 (C-7, C-7′), 38.1 (C-10), 35.2 (C-17), 30.8 (C-11); HRESIMS *m/z* 1080.7076 $[M-H]^-$ (calc. for $C_{31}H_{29}{}^{79}Br_3{}^{81}Br_3N_4O_9$, 1080.6973).

11-hydroxy-aerothionin (**15**): yellowish amorphous solid (2 mg); ^{1}H NMR (500 MHz, acetone-*d*$_6$): δ ppm 7.77 (br t, *J* = 6 Hz, 1H, NH), 7.63 (br t, *J* = 6 Hz, 1H, NH′), 6.51 (s, 2H, H-5, H-5′), 5.42 (br s, 1H, OH-1, OH-1′), 4.18 (br s, 2H, H-1, H-1′), 4.05 (m, 1H, H-11), 3.83 (d, *J* = 18 Hz, 1H, H-7a), 3.82 (d, *J* = 18 Hz, 1H, H-7′a), 3.72 (s, 6H, OCH_3), 3.56 (m, 2H, H-10), 3.44 (m, 2H, H-13), 3.18 (d, *J* = 18 Hz, 1H, H-7b), 3.16 (d, *J* = 18 Hz, 1H, H-7′b), 1.78 (m, 1H, H-12a), 1.62 (m, 1H, H-12b); ESIMS *m/z* 834.8 $[M+H]^+$ (for $C_{24}H_{26}{}^{79}Br_2{}^{81}Br_2N_4O_5$).

Aerophobin-2 (**16**): yellowish amorphous solid (140 mg); ^{1}H NMR (500 MHz, CD_3OD): δ ppm 6.53 (s, 1H, H-14), 6.42 (s, 1H, H-5), 6.20 (d, *J* = 11.6 Hz, 1H, H-12), 4.08 (s, 1H, H-1), 3.79 (d, *J* = 18 Hz, 1H, H-7a), 3.73 (s, 3H, OCH_3), 3.34 (m, 2H, H-10), 3.10 (d, *J* = 18 Hz, 1H, H-7b), 2.54 (m, 2H, H-12), 1.85 (m, 2H, H-11); ^{13}C NMR (125 MHz, CD_3OD): δ ppm 162.1 (C-9), 155.4 (C-8), 149.4 (C-3), 132.4 (C-5), 128.5 (C-13), 122.9 (C-2), 114.5 (C-4), 110.2 (C-14), 92.6 (C-6), 75.7 (C-1), 60.5 (OCH_3), 40.3 (C-7), 39.6 (C-10), 29.2 (C-11), 23.0 (C-12); HRESIMS *m/z* 506.0012 $[M+H]^+$ (calc. for $C_{16}H_{20}{}^{79}Br^{81}BrN_5O_4$, 505.9861).

Aplysinamisin-1 (**17**): yellowish amorphous solid (15 mg); ^{1}H NMR (500 MHz, CD_3OD): δ ppm 8.37 (s, 2H, NH-15′), 6.85 (s, 1H, H-14), 6.42 (s, 1H, H-5), 6.20 (d, *J* = 11.6 Hz, 1H, H-12), 5.75 (m, 1H, H-11), 4.10 (m, 3H, H-1, H-10), 3.80 (d, *J* = 18 Hz, 1H, H-7a), 3.73 (s, 3H, OCH_3), 3.11 (d, *J* = 18 Hz, 1H, H-7b); HRESIMS *m/z* 504.0015 $[M+H]^+$ (calc. for $C_{16}H_{18}{}^{79}Br^{81}BrN_5O_4$, 503.9704).

7R,11S [3,5-dibromo-4-[(2-oxo-5-oxazolidinyl)]methoxyphenyl]-2-oxazolidinone (**18**): amorphous solid (3 mg); $[\alpha]_D^{25}$ -10.5 (*c* 0.5, MeOH); $[\alpha]_D^{25}$ -8.5 (*c* 0.2, Acetone); ^{1}H NMR (300 MHz, CD_3CN): δ ppm 7.64 (s, 2H, H-3, H-5), 5.77 (br s, 1H, NH), 5.67 (br s, 1H, NH′), 5.51 (t, *J* = 8.0, 16.0 Hz, 1H, H-7), 4.97 (m, 1H, H-11), 4.19 (m, 2H, H-10), 3.88 (dd, *J* = 1.0, 9. 0 Hz, 1H, H-8a), 3.65 (m, 2H, H-12), 3.38 (dd, *J* = 1.0, 7.4 Hz, 1H, H-8b); ^{13}C NMR (75 MHz, CD_3CN): δ ppm 160.1 (C-9, C-13), 153.6 (C-1), 139.9 (C-4), 131.7 (C-3, C-5), 119.1 (C-2, C-6), 76.4 (C-7), 75.5 (C-11), 74.0 (C-10), 48.4 (C-8), 42.6 (C-12); HRESIMS *m/z* 458.9014 $[M+Na]^+$ (calc. for $C_{13}H_{11}{}^{79}Br^{81}BrN_2O_5Na$, 458.8990).

Subereaphenol (**19**): colourless amorphous solid (3 mg); ^{1}H NMR (500 MHz, MeOD): δ ppm 6.98 (s, 1H), 3.79 (s, 2H); ^{13}C NMR (125 MHz, MeOD): δ ppm 175.1, 151.3, 144.8, 124.4, 118.7, 116.9, 110.4, 37.2; HRESIMS *m/z* 324.8551 $[M+Na]^+$ (calc. for $C_8H_5{}^{79}Br^{81}BrO_4Na$, 324.8534).

Pseudoceralidinone A (**20**) : yellow amorphous solid (5 mg); $[\alpha]_D^{25}$ +3.0 (*c* 0.5, MeOH); UV (MeOH) λ_{max} (ϵ) 208 (25000), 276 (1800) nm; IR ν_{max} 3380, 1740, 1640, 1460, 1225 cm^{-1}; ^{1}H and ^{13}C NMR data, **Table 5**; (^{1}H NMR (CD_3OD, 500 MHz, 298 K): δ 4.00 (dd, 7.1; 6.6, H-2) 3.44 (dd, 8.6; 6.6, H-2), 5.63 (dd, 8.6; 7.1, H-3), 7.68 (s, H-5, H-5′), 4.17 (t, 5.5, H-8), 2.32 (m, H-9), 3.53 (t, 7.7, H-10), 2.98 (s, H-11, H-12). ^{13}C NMR (CD_3OD, 125 MHz, 298 K): δ 161.5 (C-1), 43.7 (C-2), 77.1 (C-3), 138.7 (C-4), 131.5 (C-5, C-5′), 119.5 (C-6), 154.0 (C-7), 71.3 (C-8), 26.4 (C-9), 57.0 (C-10), 43.7 (C-11), 43.7 (C-12); HRESIMS *m/z* 422.9749 $[M+H]^+$ (calc. for $C_{14}H_{19}{}^{79}Br^{81}BrN_2O_3$, 422.9742).

Figure S1: ^{1}H NMR spectrum of 17*S*-*epi*-fistularin-3 (**1**) in acetone-d_6 (500 MHz)

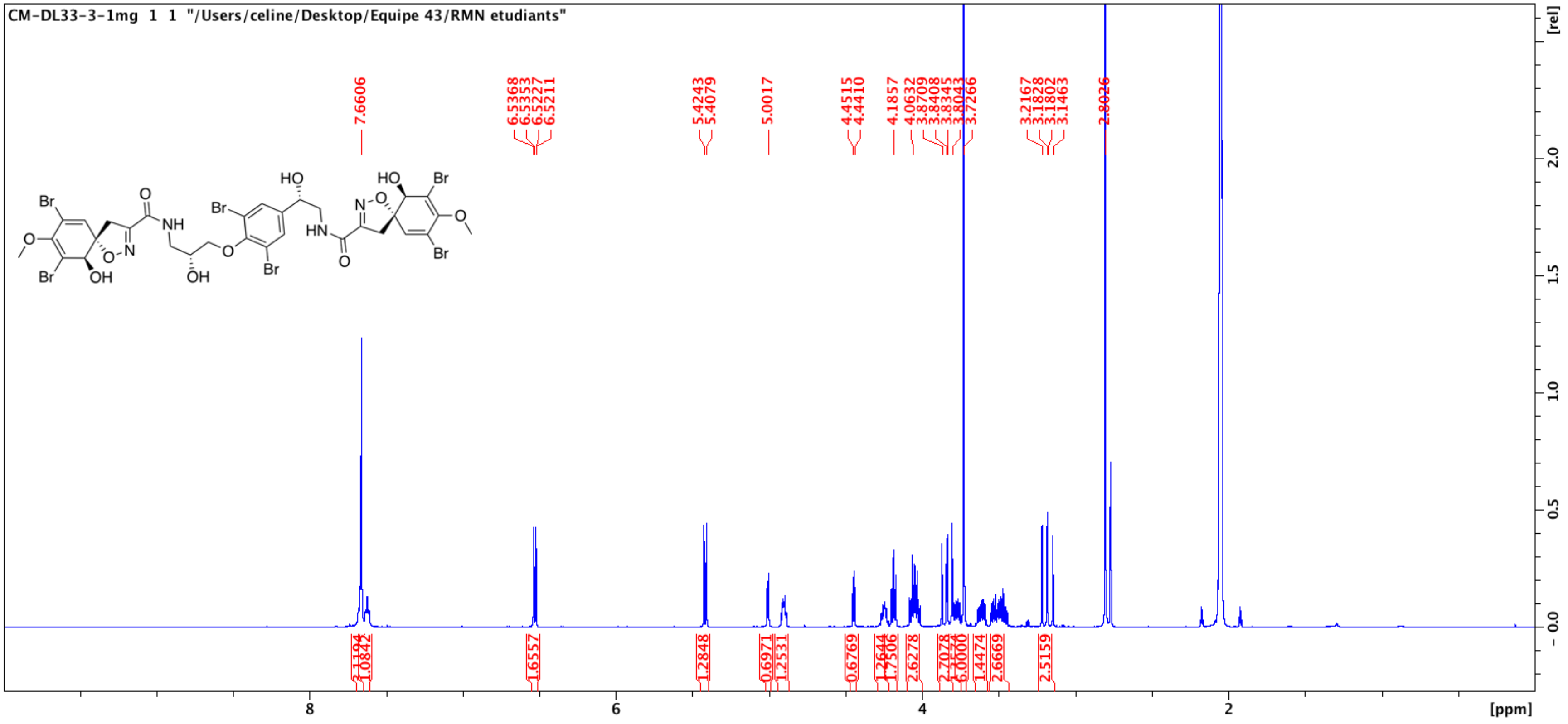

Figure S2: ^{13}C NMR spectrum of 17*S*-*epi*-fistularin-3 (**1**) in acetone-d_6 (500 MHz)

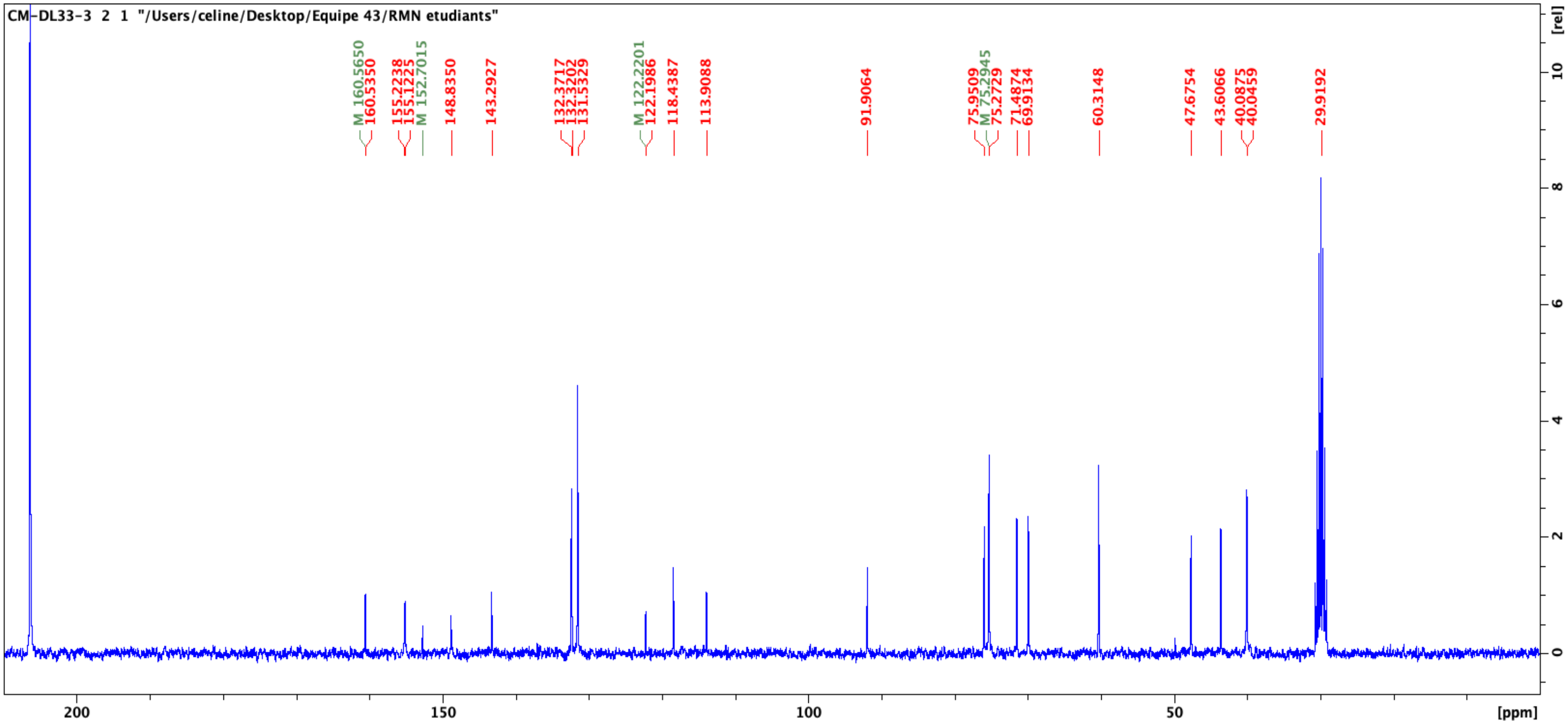

Figure S3: ^{13}C NMR spectrum of 17*S*-*epi*-fistularin-3 (**1**) in acetone-*d*$_6$ (500 MHz), Zoom on C-11 and C-17.

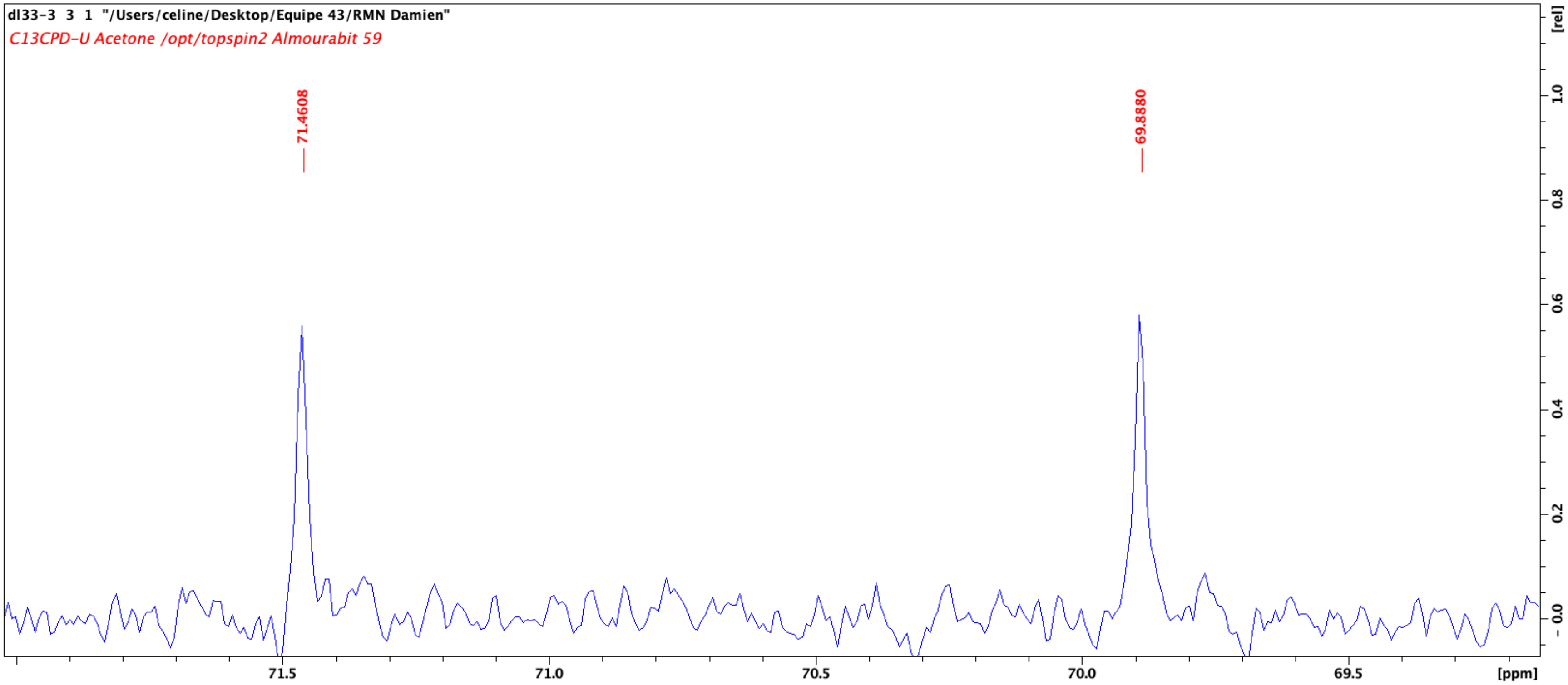

Figure S4: ECD spectrum of 17*S*-*epi*-fistularin-3 (**1**) in MeOH (c 0.15 mM).

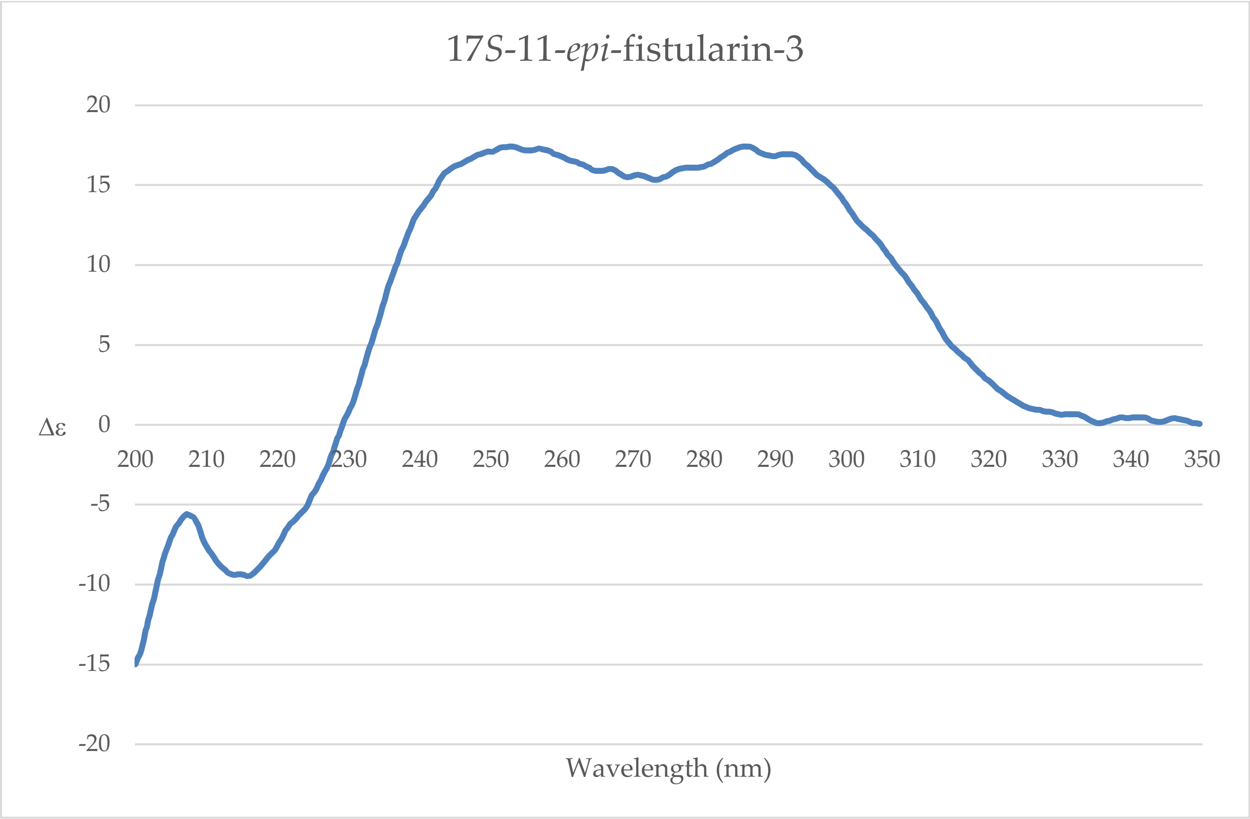

Figure S5: ^{1}H NMR spectrum of the (*S*)-MPTA ester of **1** in acetone-d_6 (500 MHz).

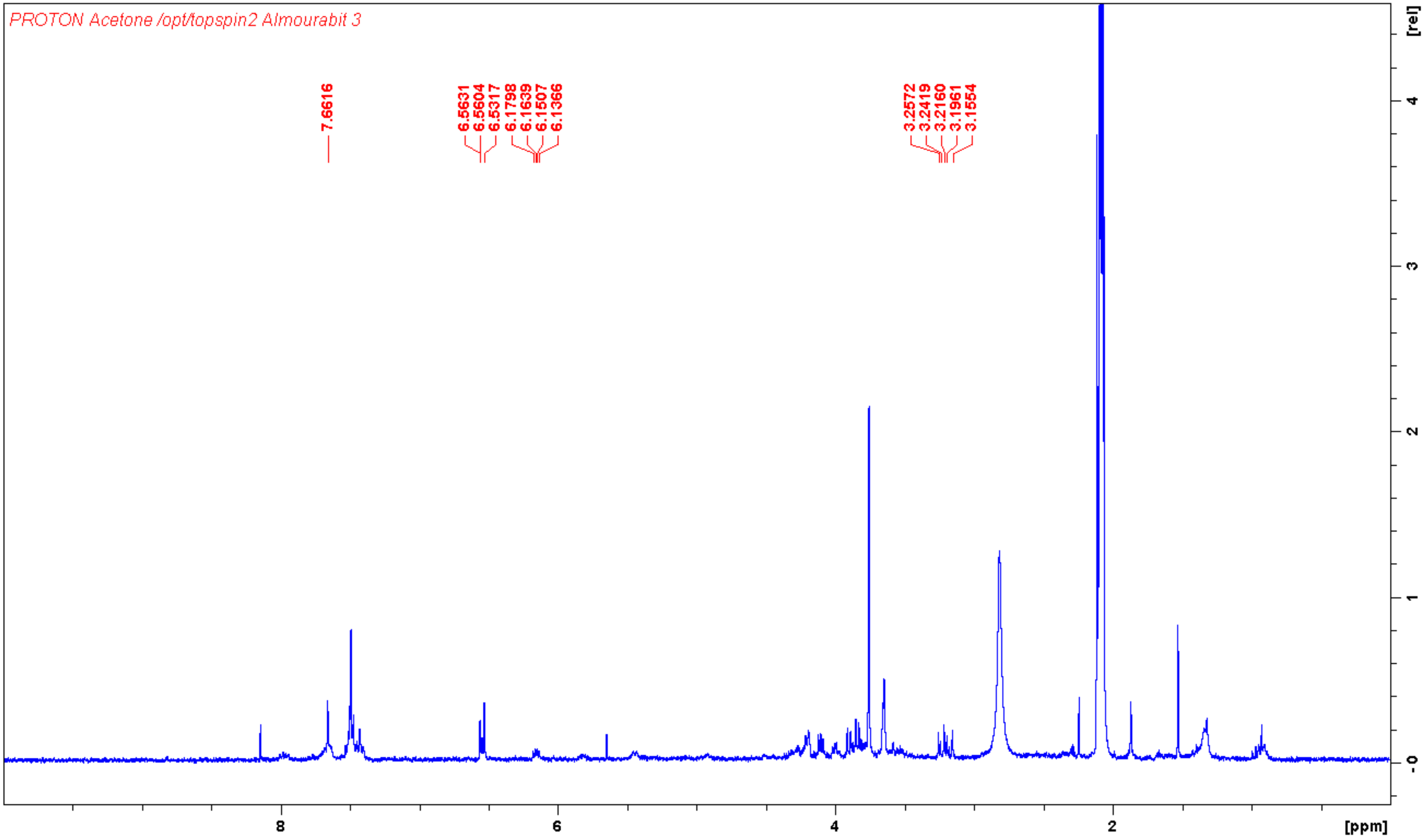

Figure S6: ^{1}H NMR spectrum of the (*R*)-MPTA ester of **1** in acetone-d_6 (500 MHz).

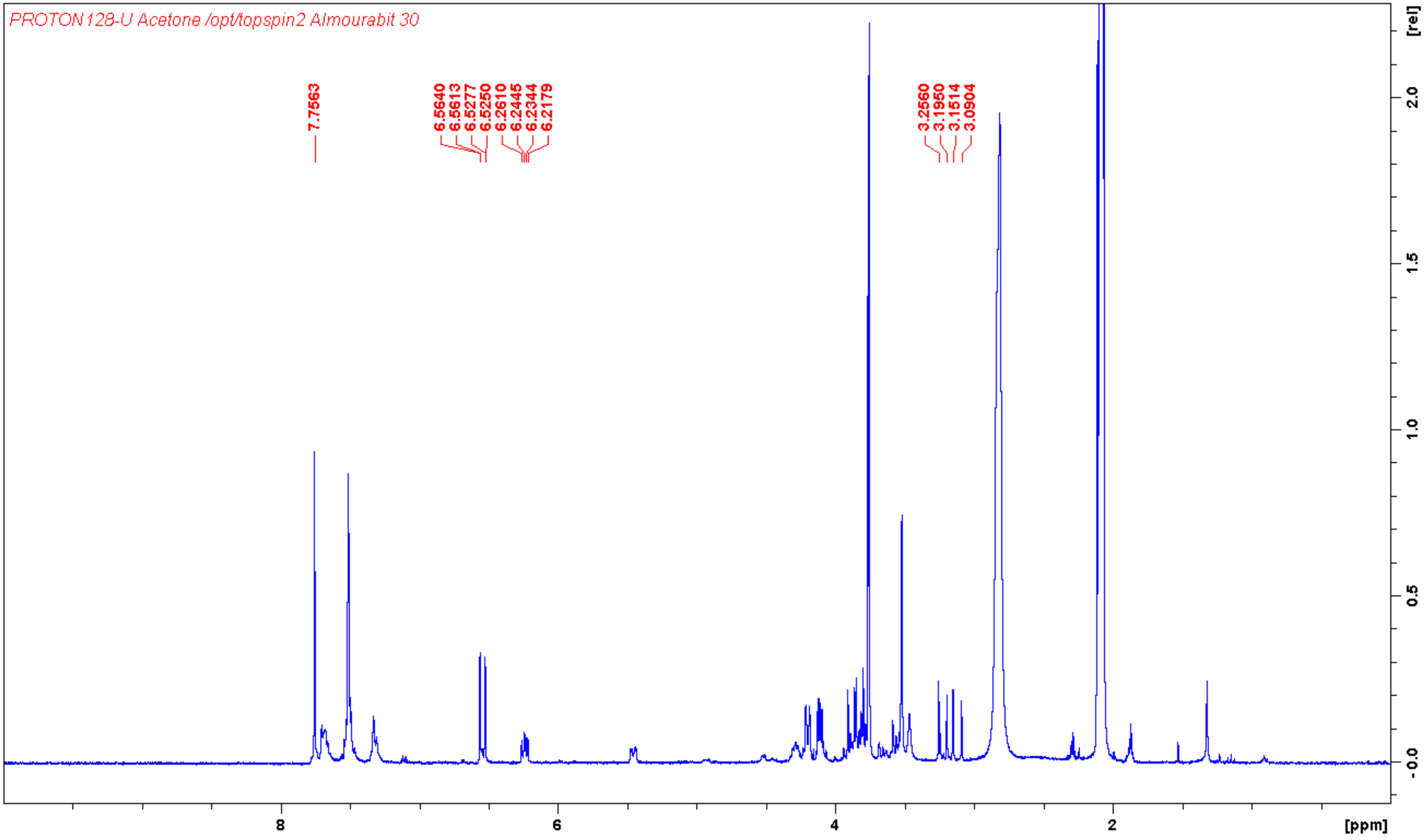

Figure S7: HPLC Chromatogram (UV) of Conversion of 11-epi-fistularin-3 (**1**) to compounds **2 – 7** and agelorins A/B

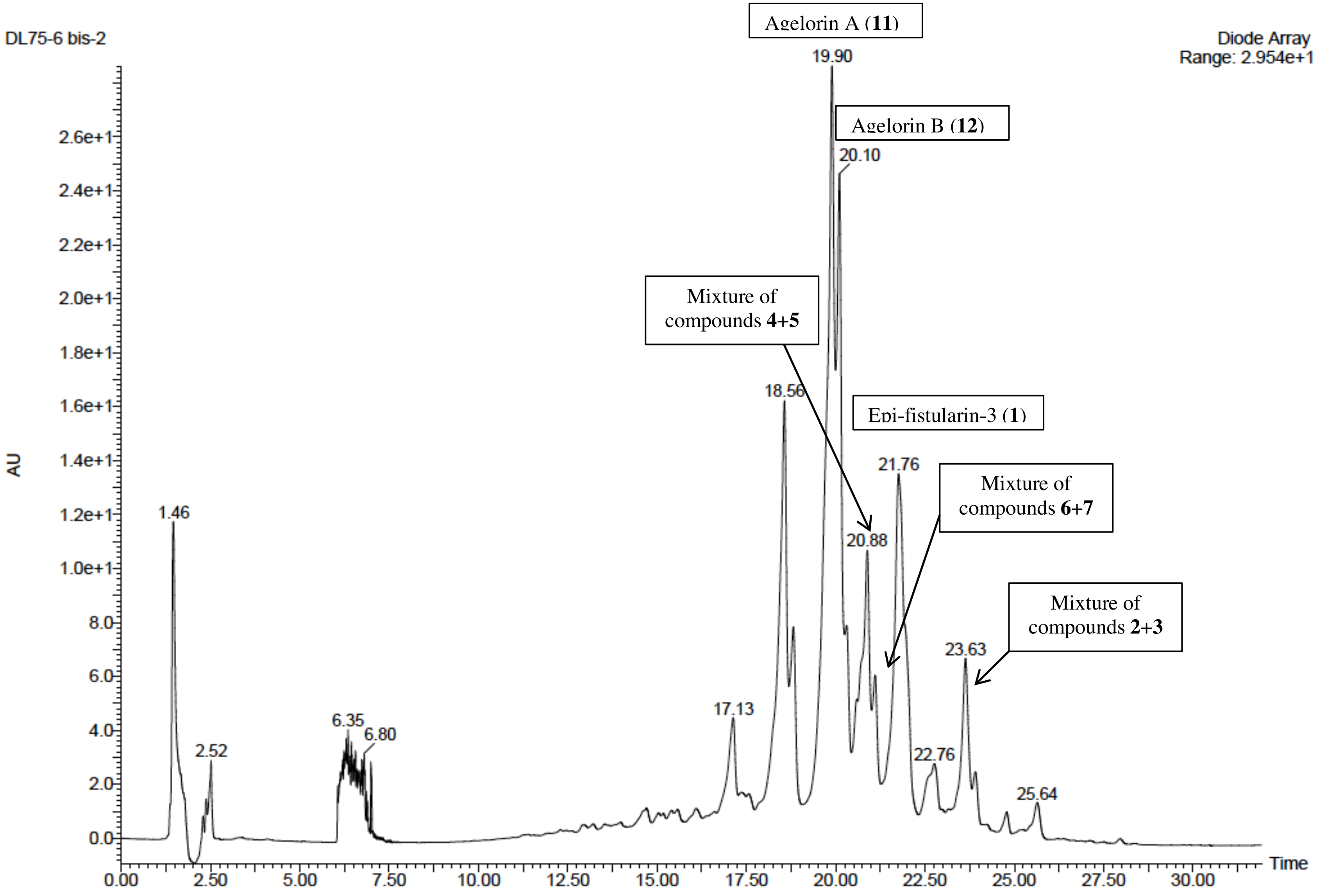

Figure S8: ^{1}H NMR spectrum of suberein-1 (**2**) in acetone-d_6 (500 MHz).

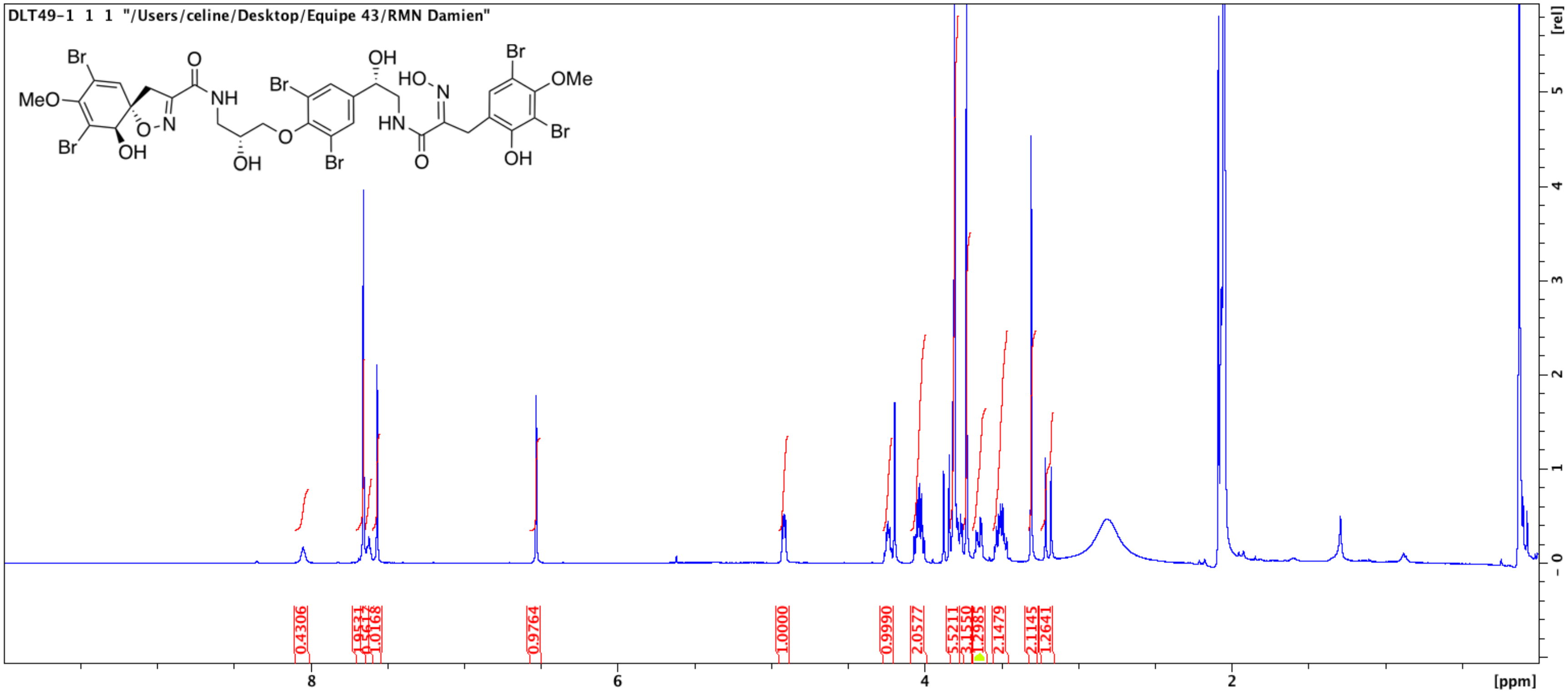

Figure S9: ^{13}C NMR spectrum of suberein-1 (**2**) in acetone-d_6 (125 MHz).

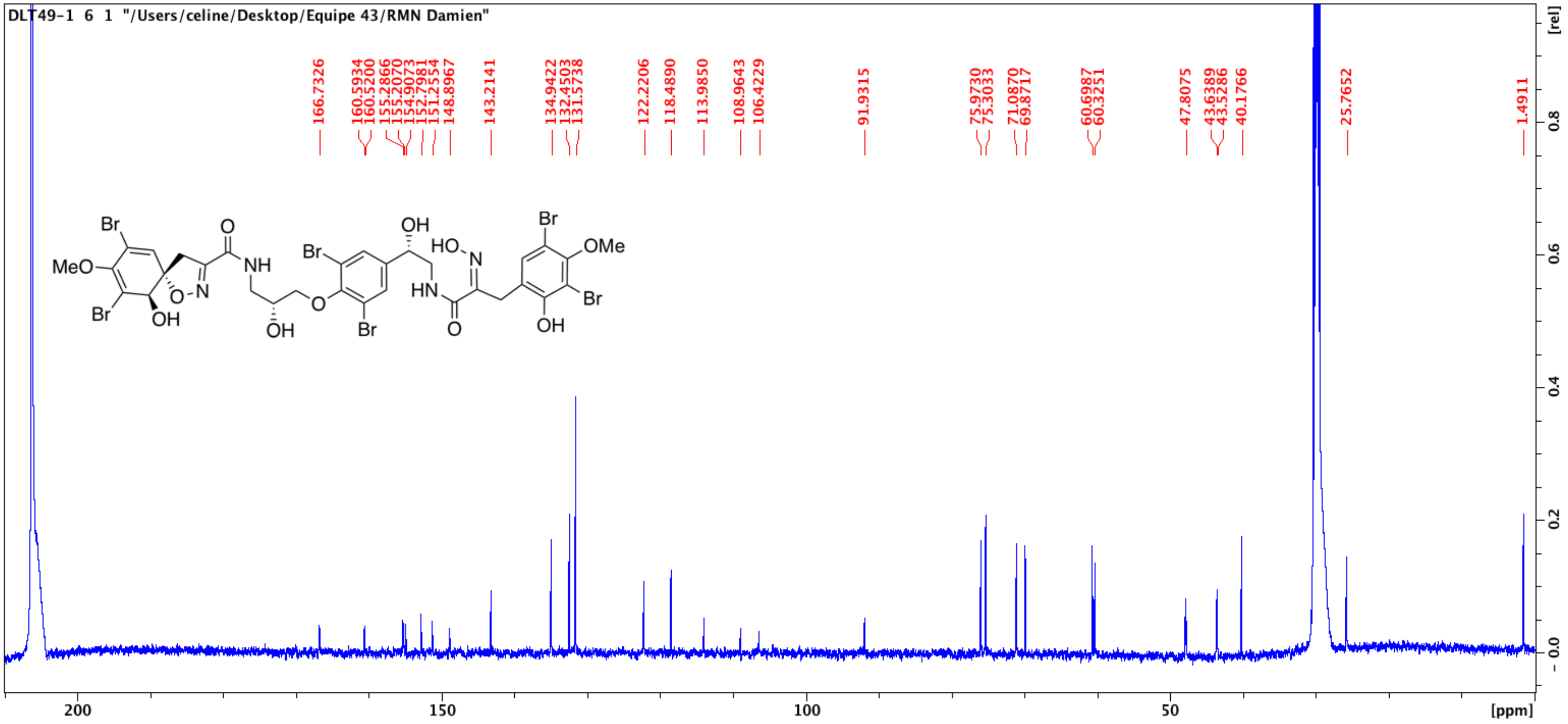

Figure S10: ^{1}H-^{1}H COSY NMR spectrum of suberein-1 (**2**) in acetone-*d*₆ (500 MHz).

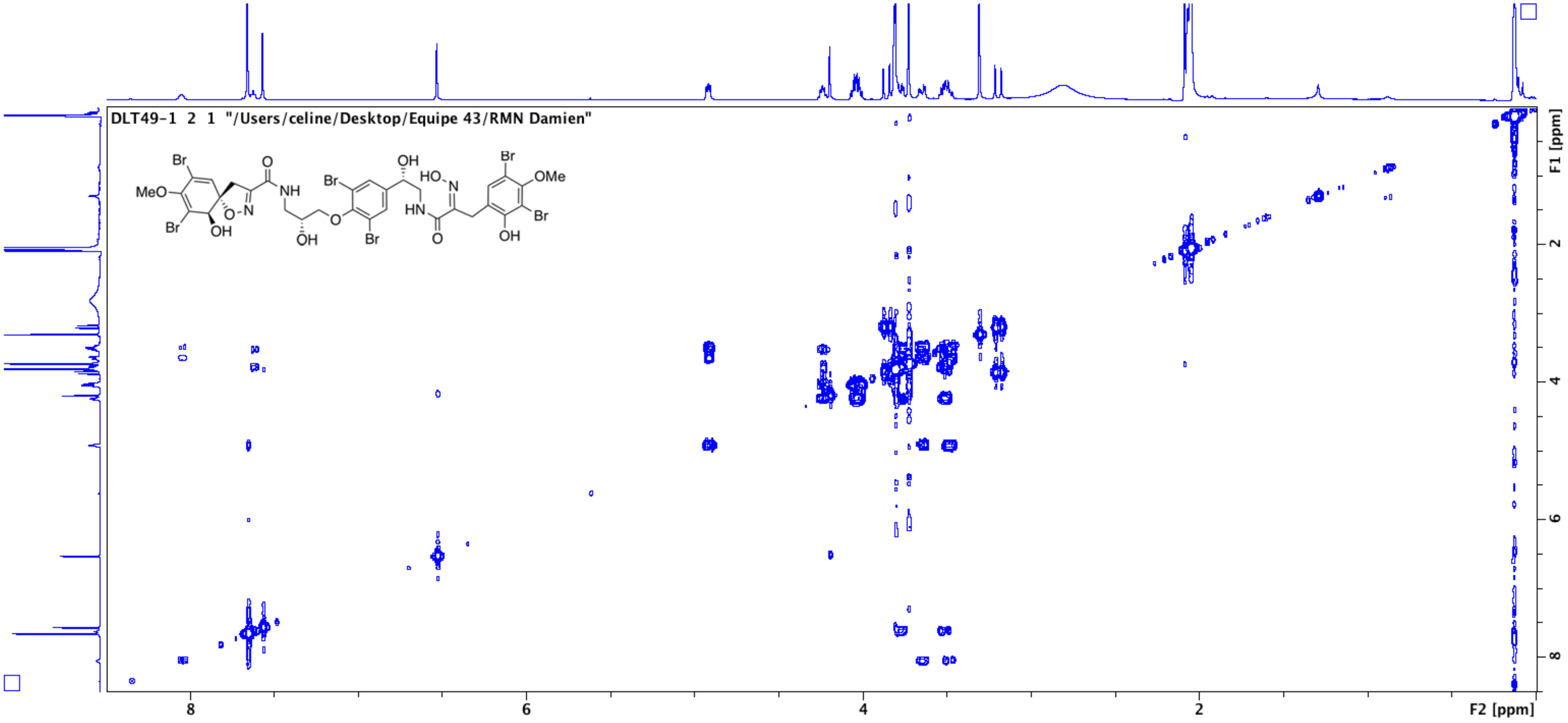

Figure S11: ^1H-^{13}C HSQC NMR spectrum of suberein-1 (**2**) in acetone-d_6 (500 MHz).

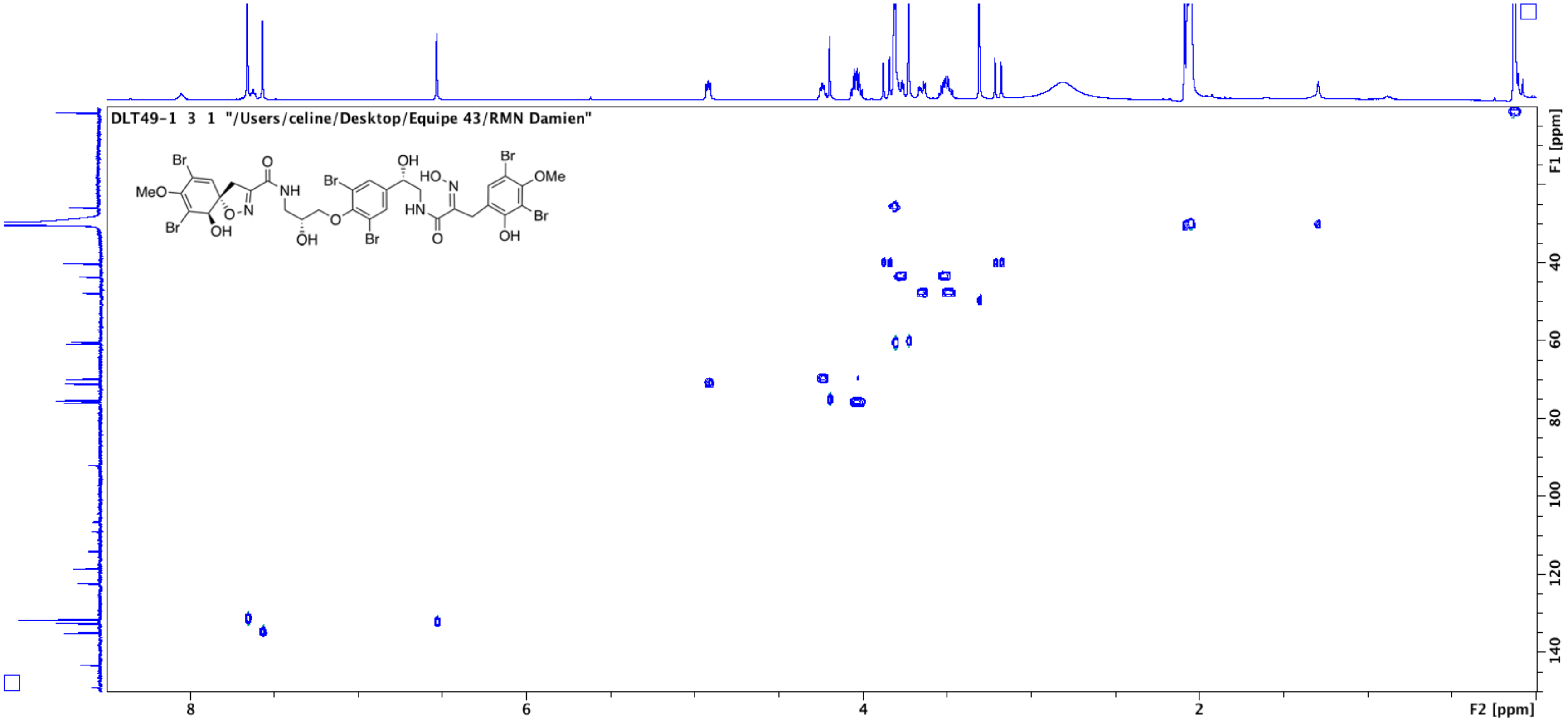

Figure S12: ^{1}H-^{13}C HMBC NMR spectrum of suberein-1 (**2**) in acetone-d_6 (500 MHz).

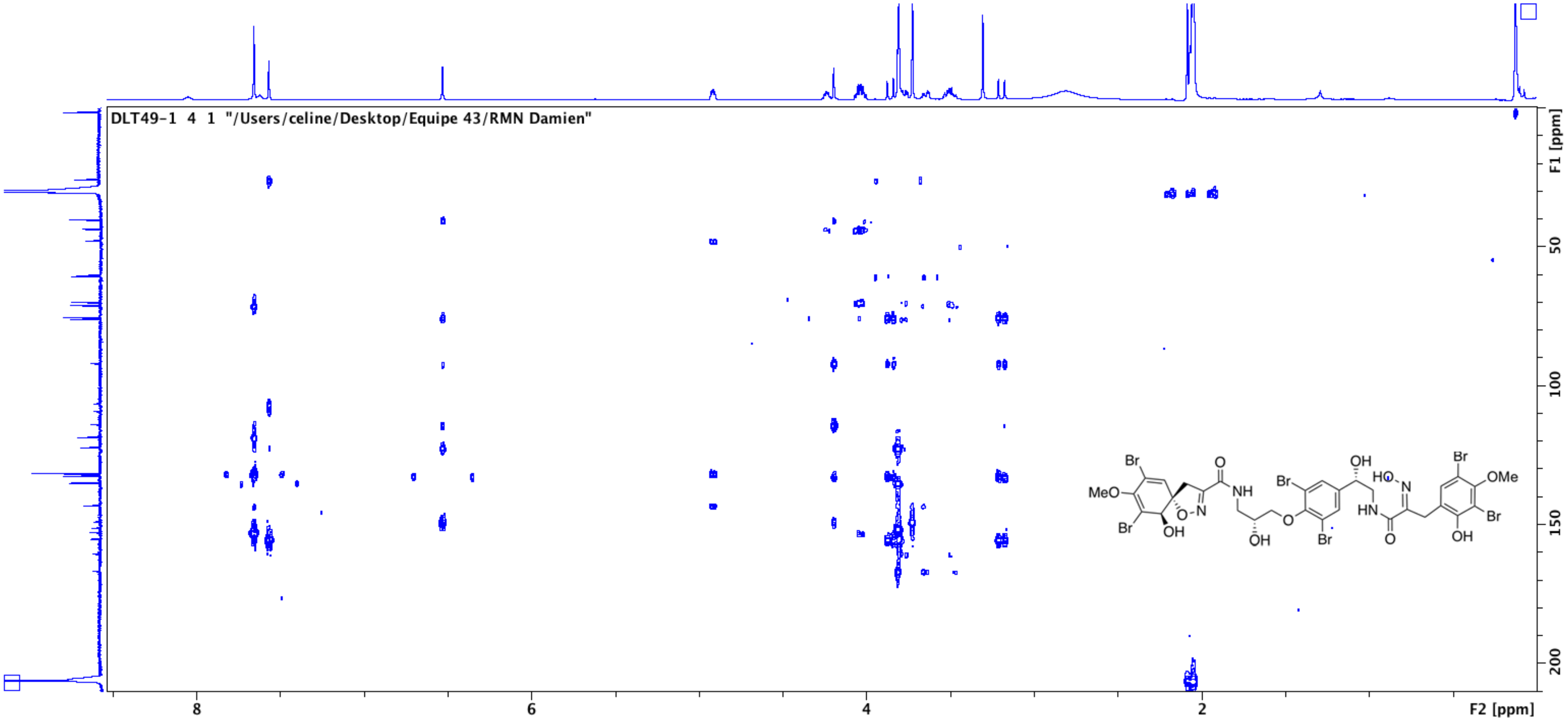

Figure S13: HR-ESI mass spectrum of suberein-1 (**2**).

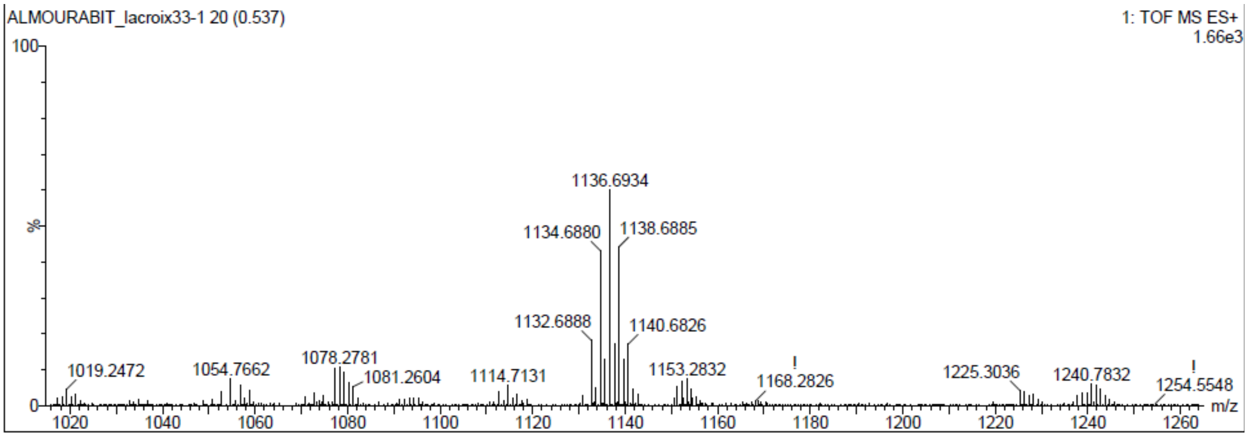

Figure S14: ECD spectrum of suberein-1 (**2**) in MeOH (*c* 0.15 mM).

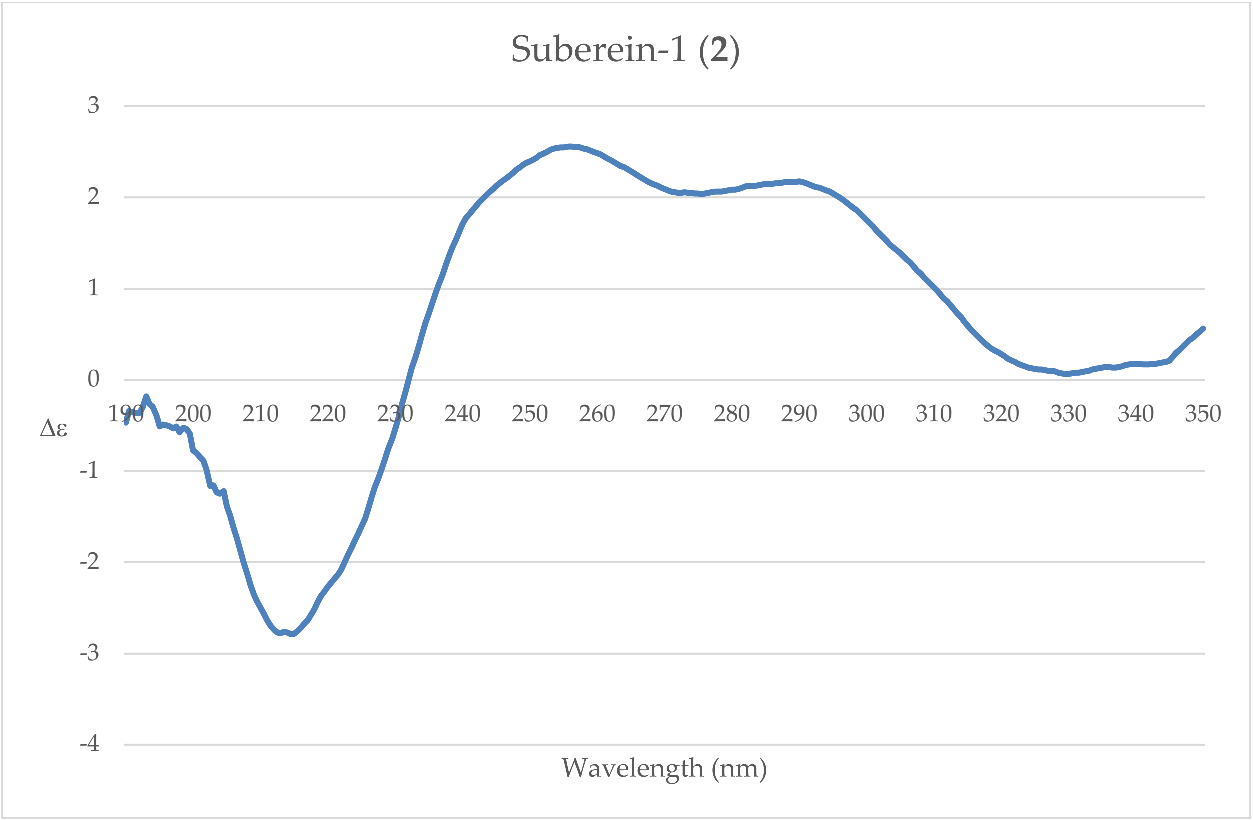

Figure S15: ^{1}H NMR spectrum of suberein-2 (**3**) in acetone-d_6 (600 MHz).

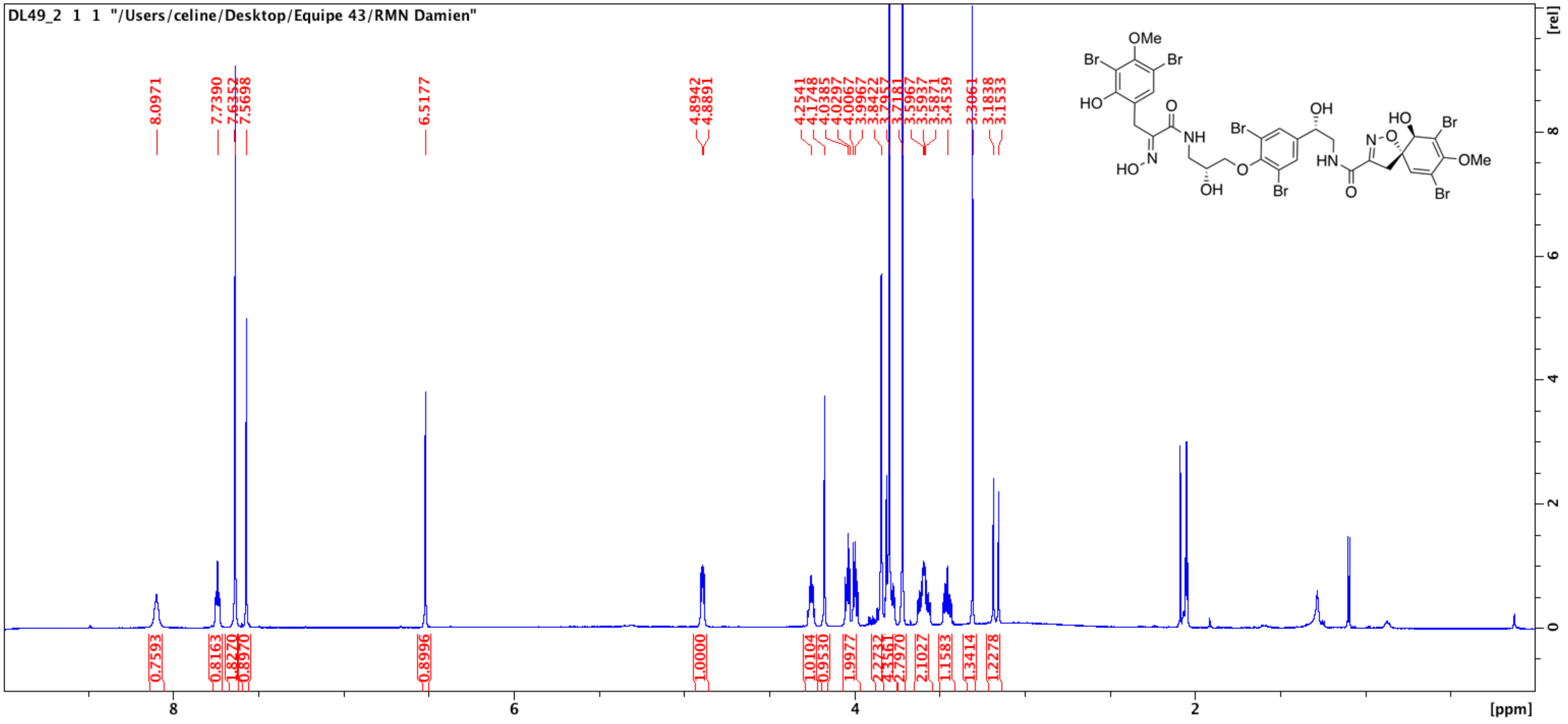

Figure S16: ^{13}C NMR spectrum of suberein-2 (**3**) in acetone-*d*$_6$ (150 MHz).

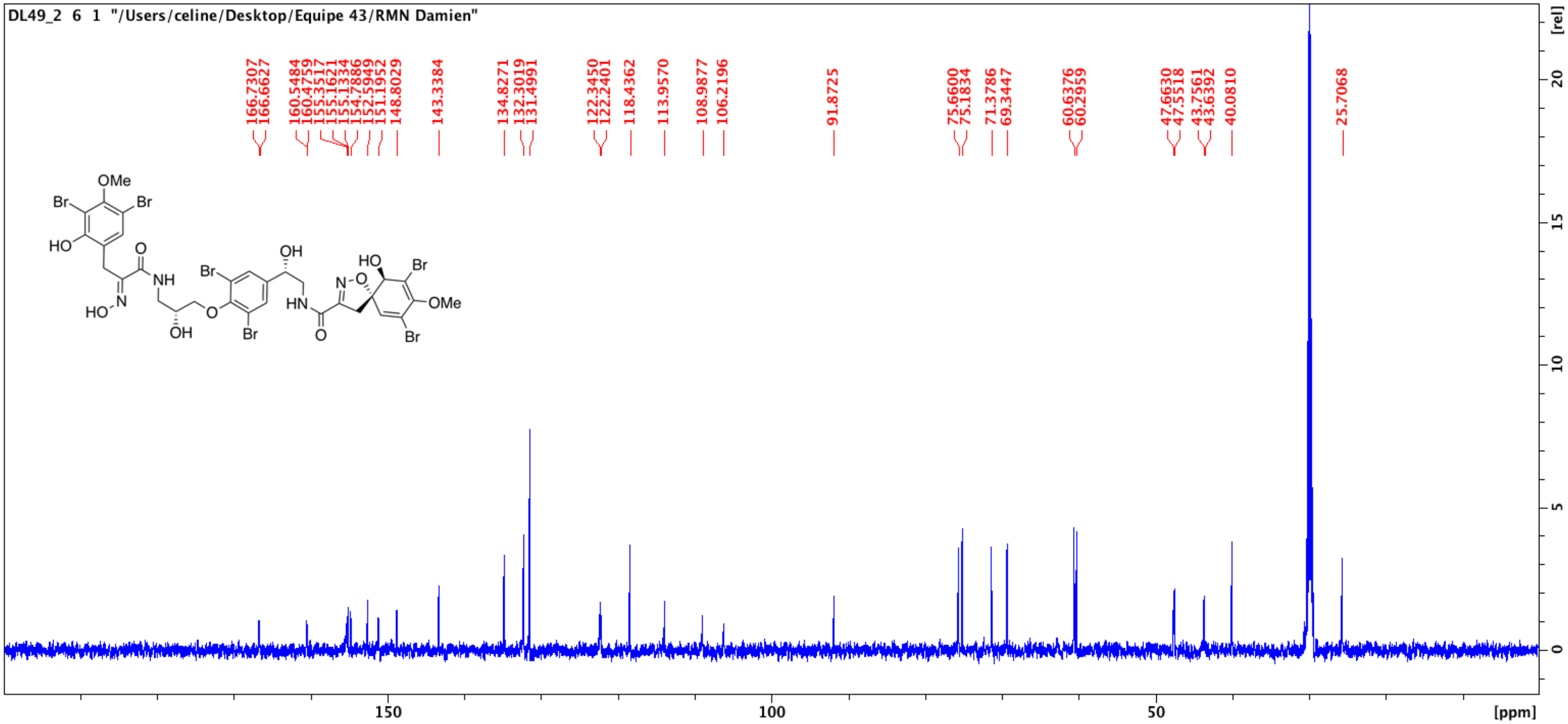

Figure S17: ^{1}H-^{1}H COSY NMR spectrum of suberein-2 (**3**) in acetone-d_6 (600 MHz).

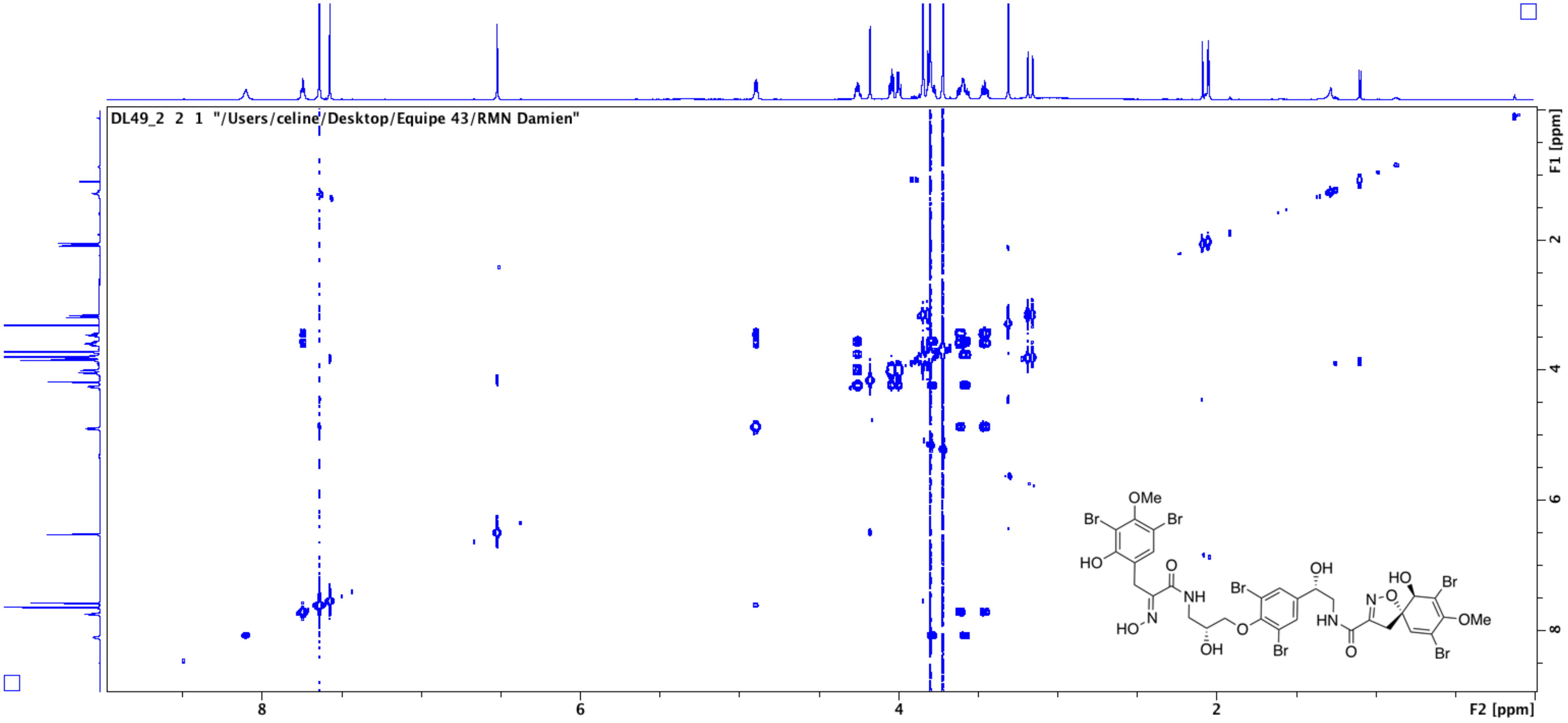

Figure S18: ^{1}H-^{13}C HSQC NMR spectrum of suberein-2 (**3**) in acetone-d_6 (600 MHz).

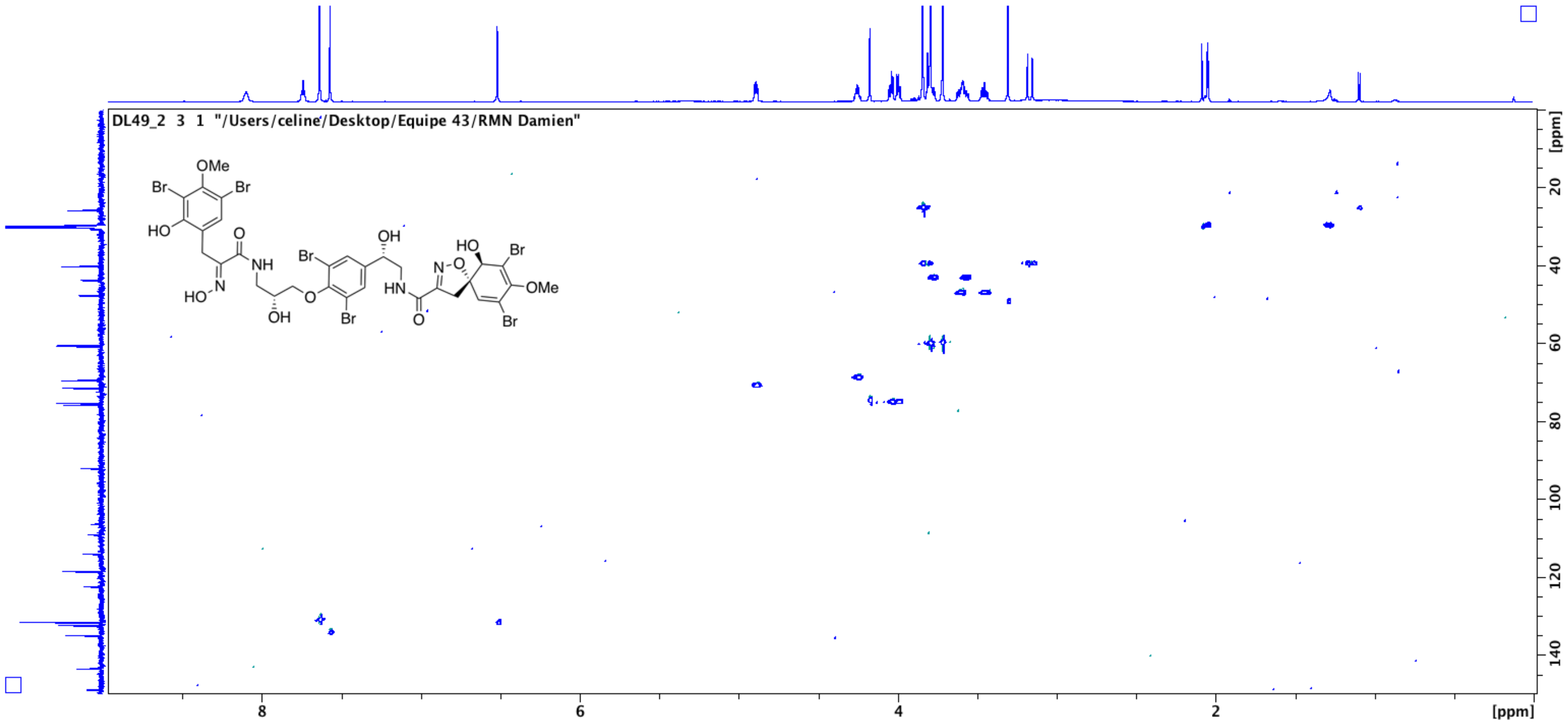

Figure S19: ^1H-^{13}C HMBC NMR spectrum of suberein-2 (**3**) in acetone-d_6 (600 MHz).

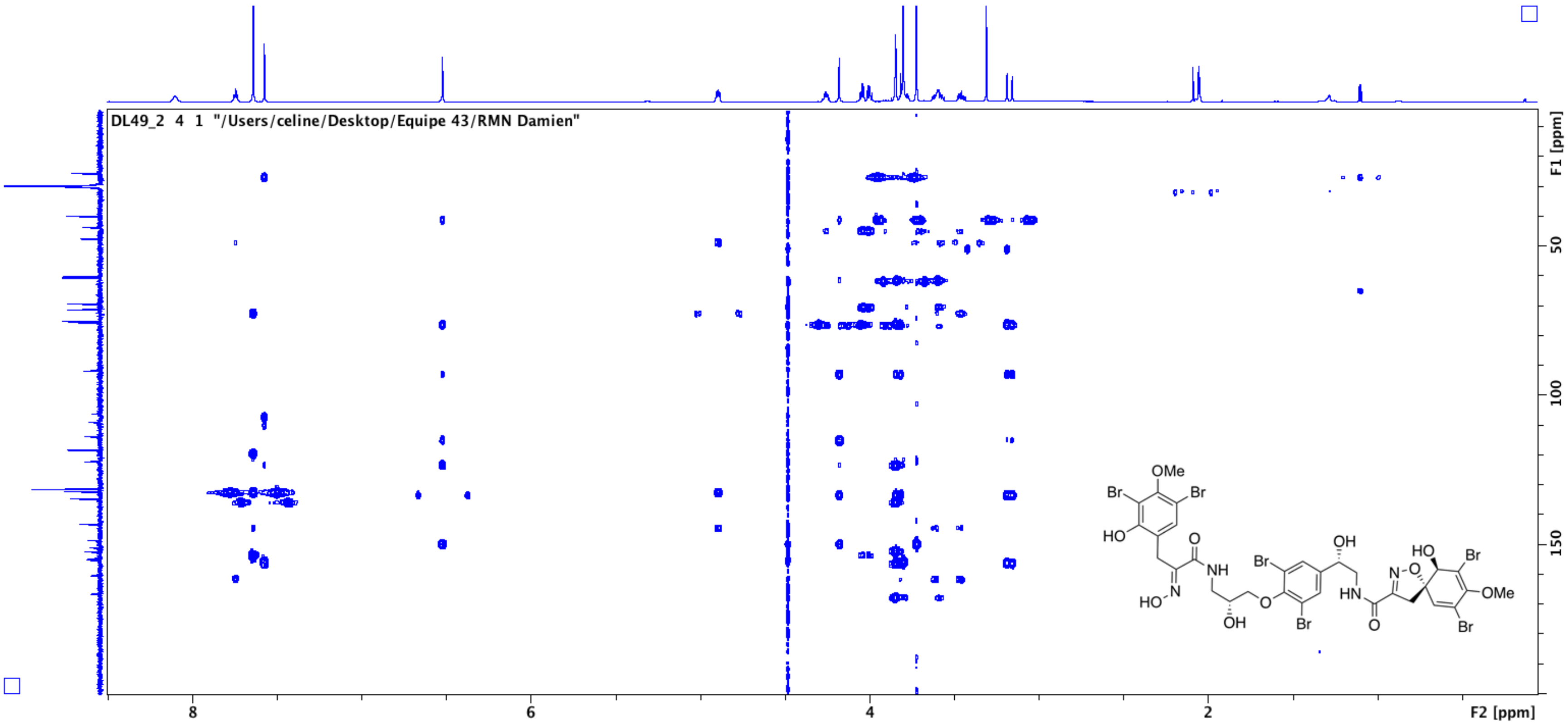

Figure S20: HRESMS-MS fragmentation spectra of suberein-2 (**3**) ([M+Na]+ and [M+H]+ respectively)

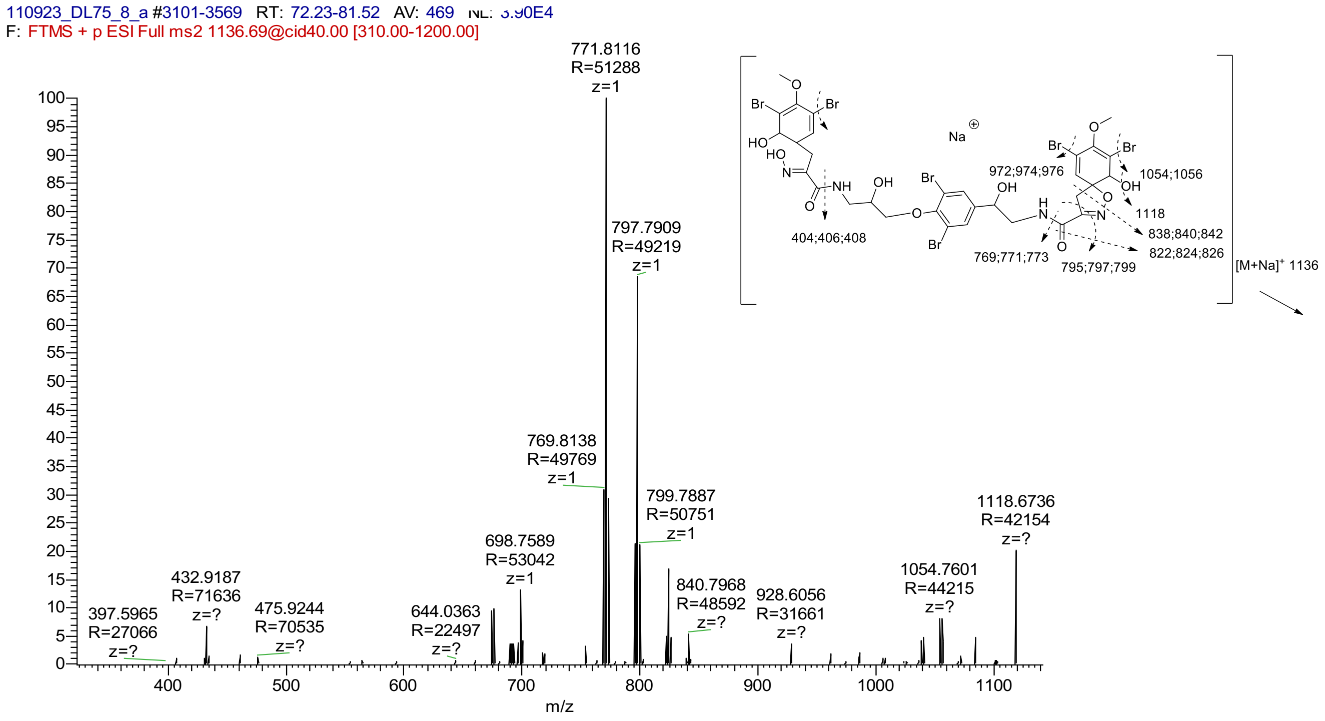

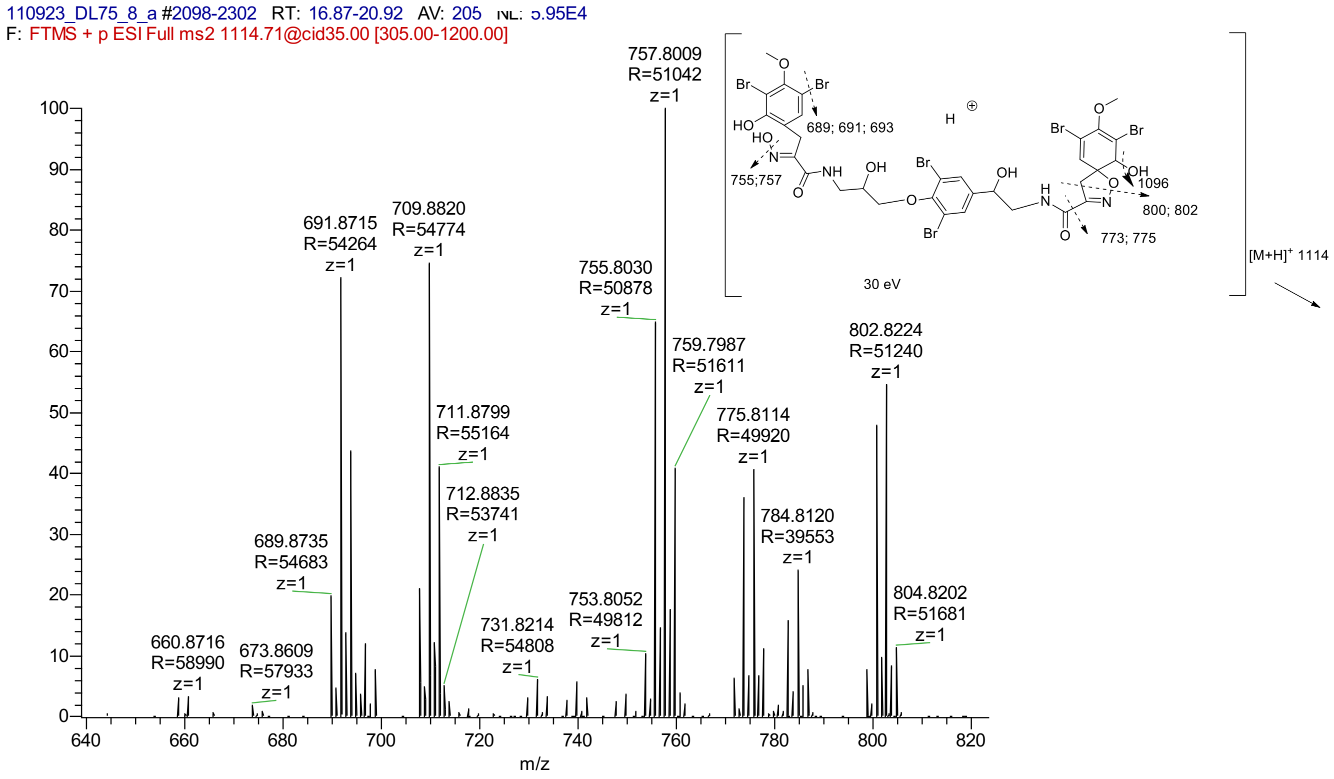

Figure S21: ECD spectrum of suberein-2 (**3**) in MeOH (c 0.15 mM).

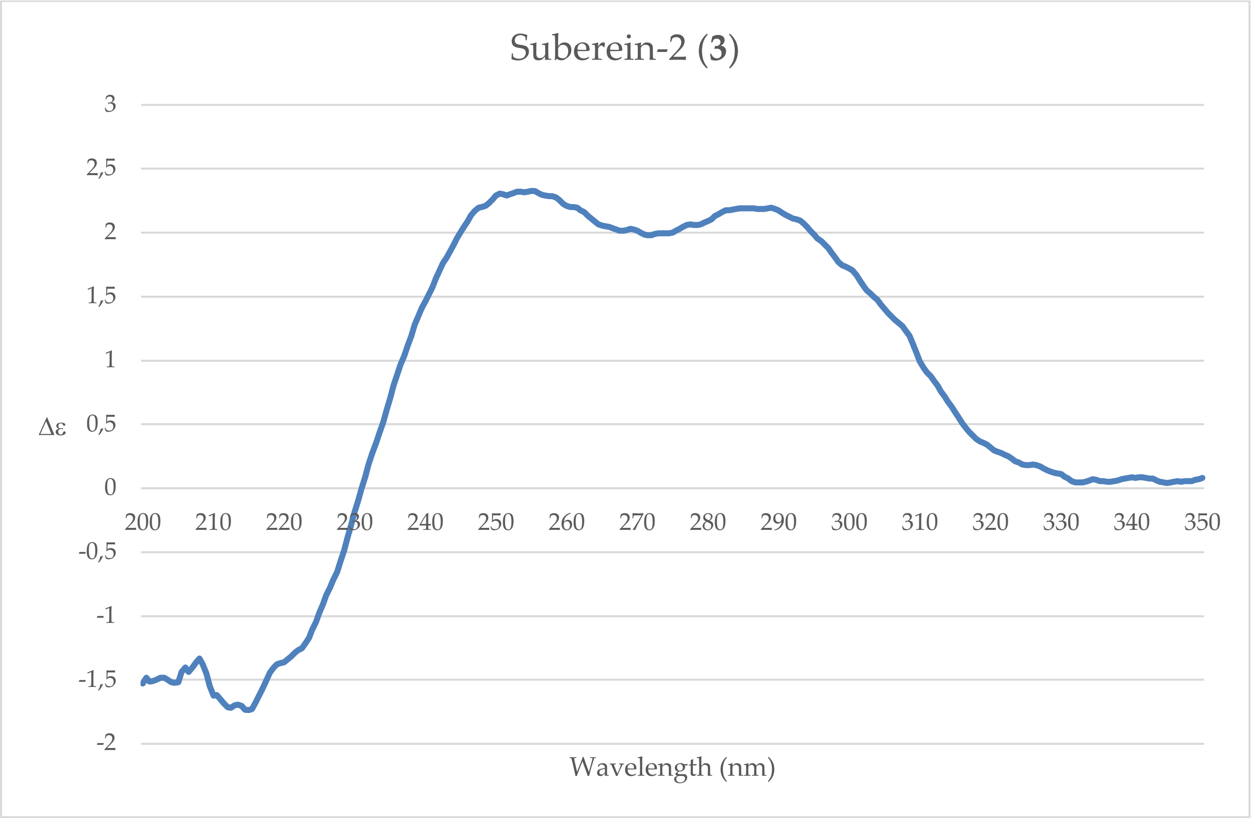

Figure S22: ^{1}H NMR spectrum of the mixture of suberein-3 (**4**) and suberein-4 (**5**) in acetone-d_6 (500 MHz).

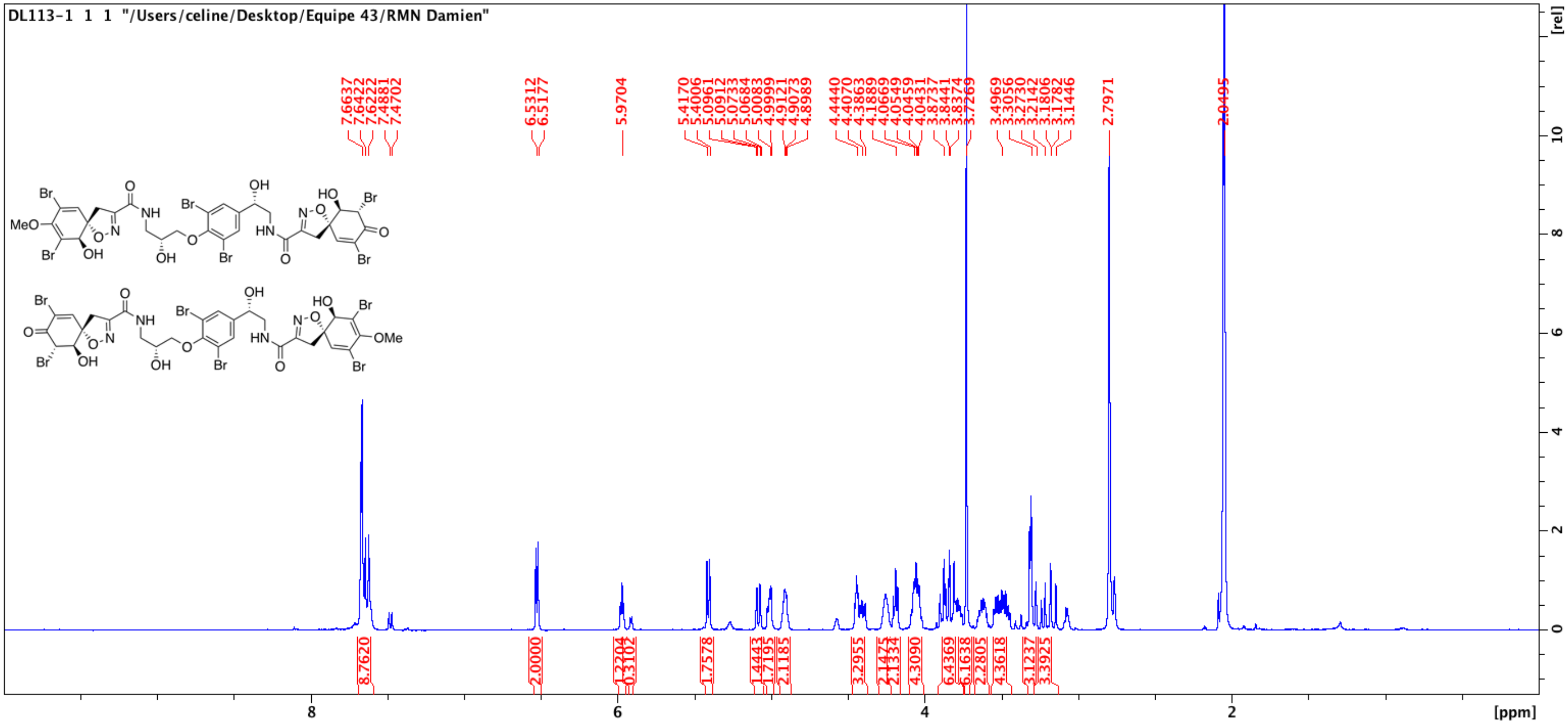

Figure S23: ^{13}C NMR spectrum of the mixture of suberein-3 (**4**) and suberein-4 (**5**) in acetone-d_6 (125 MHz).

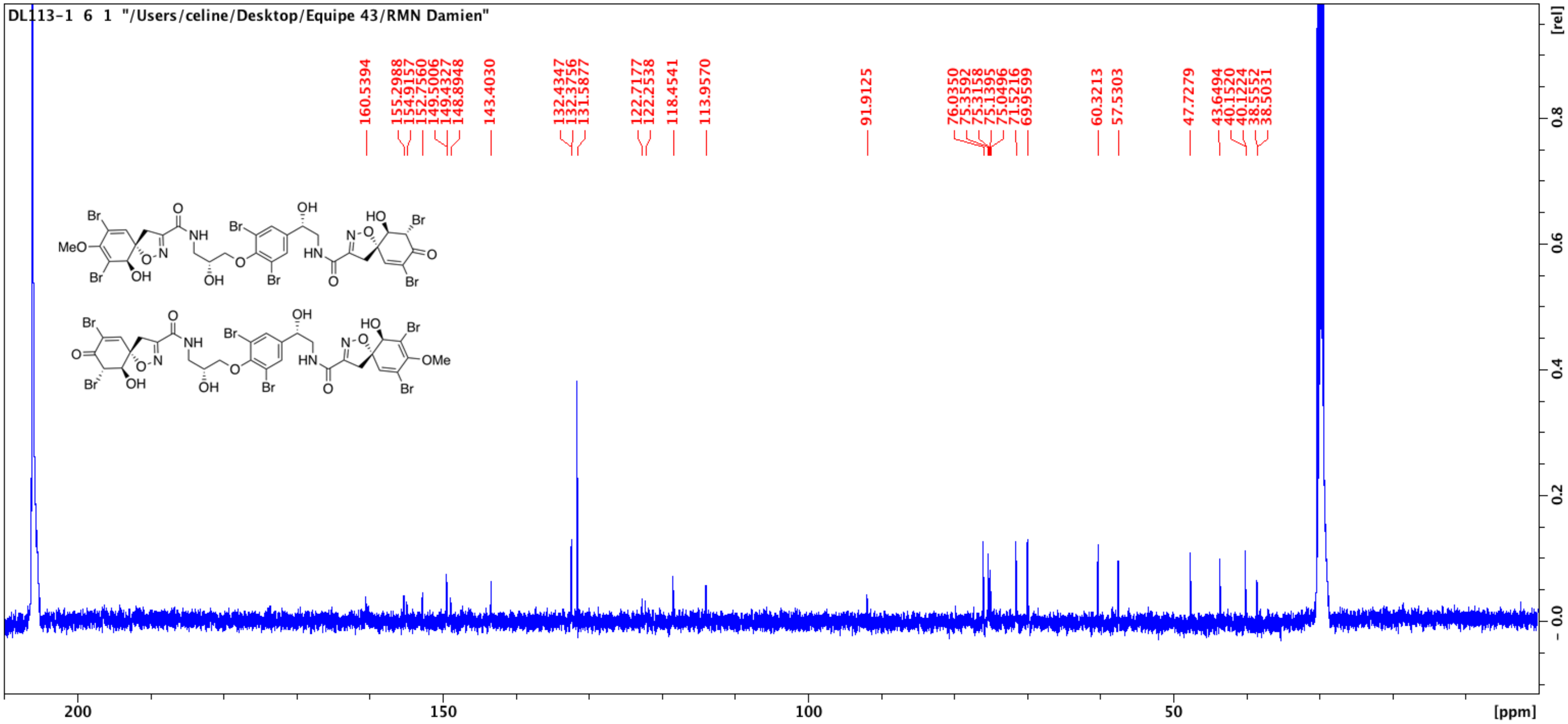

Figure S24: ^{1}H-^{1}H COSY NMR spectrum of the mixture of suberein-3 (**4**) and suberein-4 (**5**) in acetone-d_6 (500 MHz).

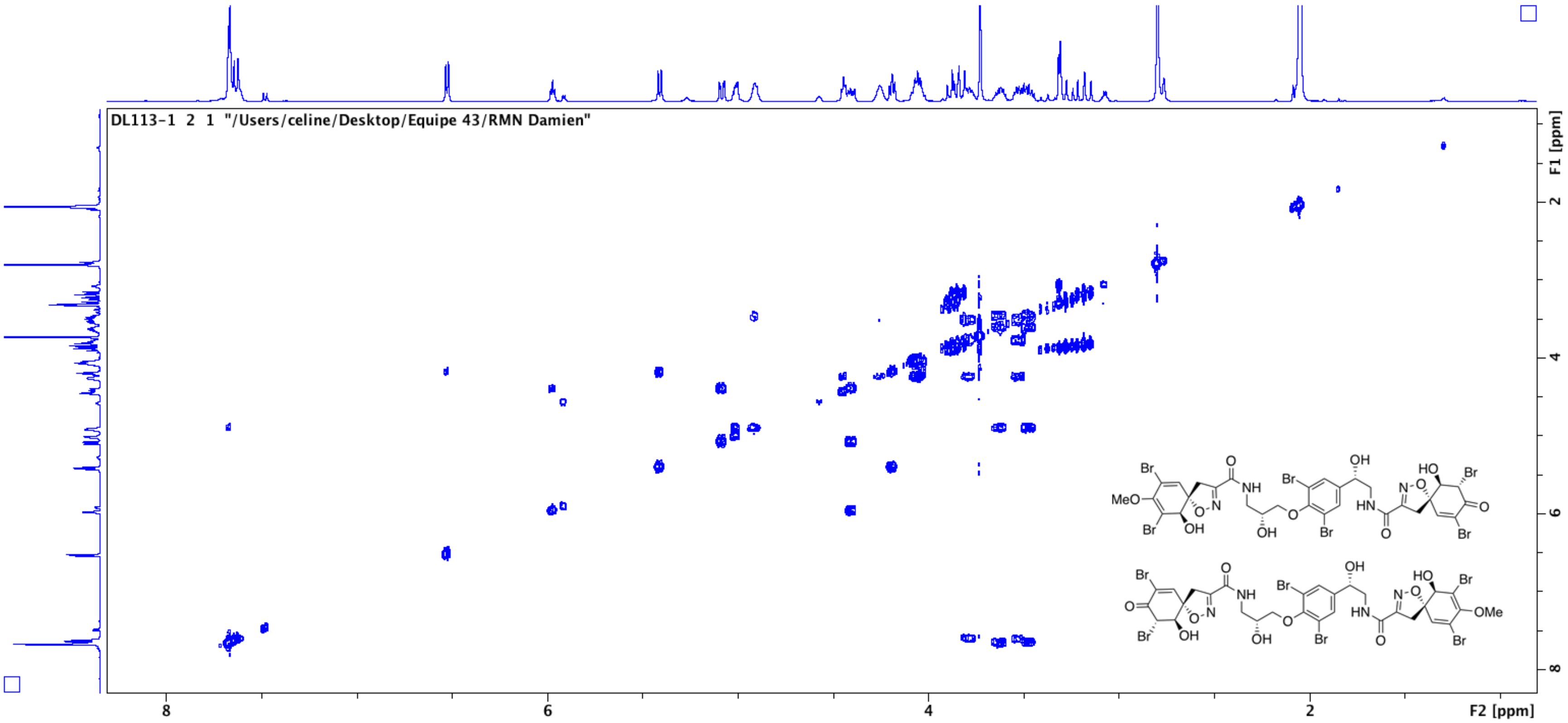

Figure S25: ^{1}H-^{13}C HSQC NMR spectrum of the mixture of suberein-3 (**4**) and suberein-4 (**5**) in acetone-d_6 (500 MHz).

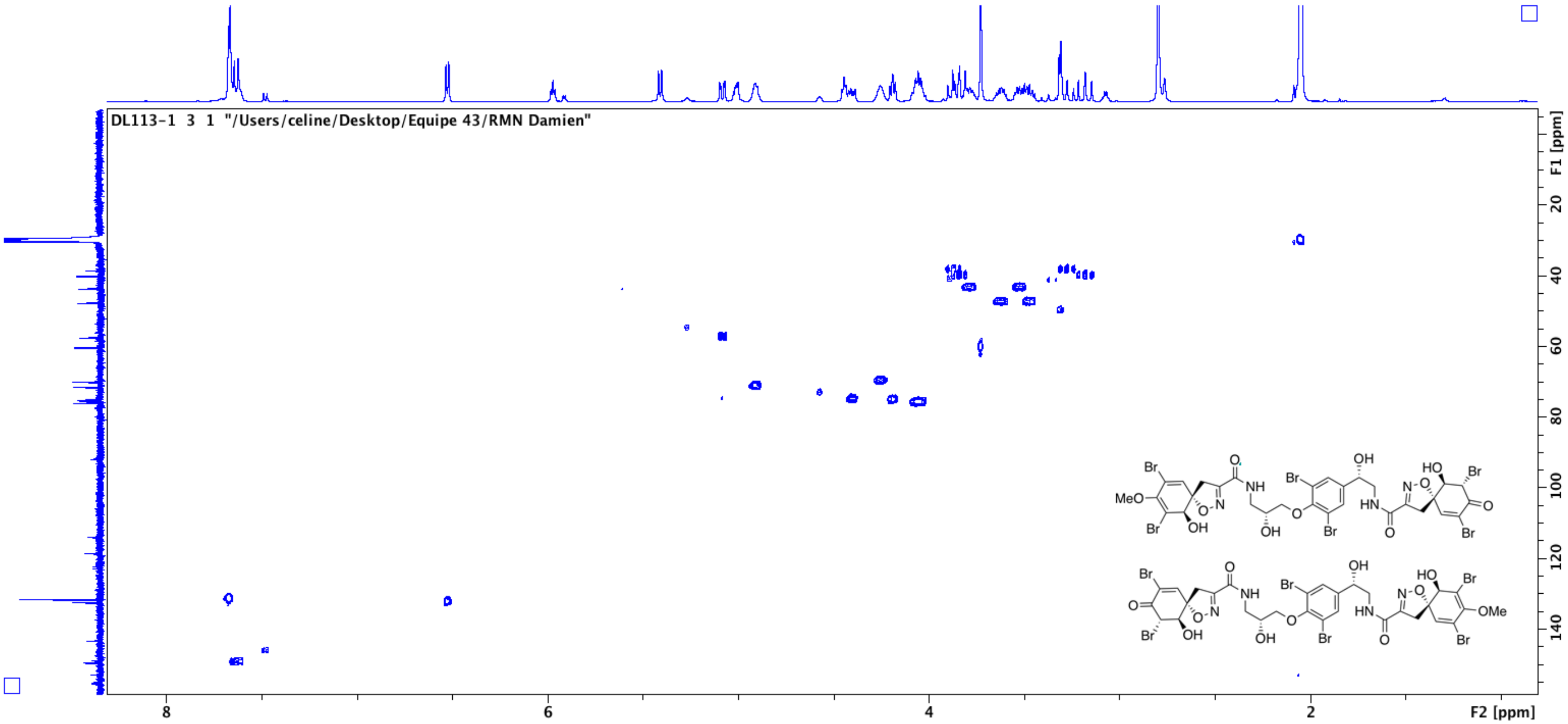

Figure S26: ^{1}H-^{13}C HMBC NMR spectrum of the mixture of suberein-3 (**4**) and suberein-4 (**5**) in acetone-d_6 (500 MHz).

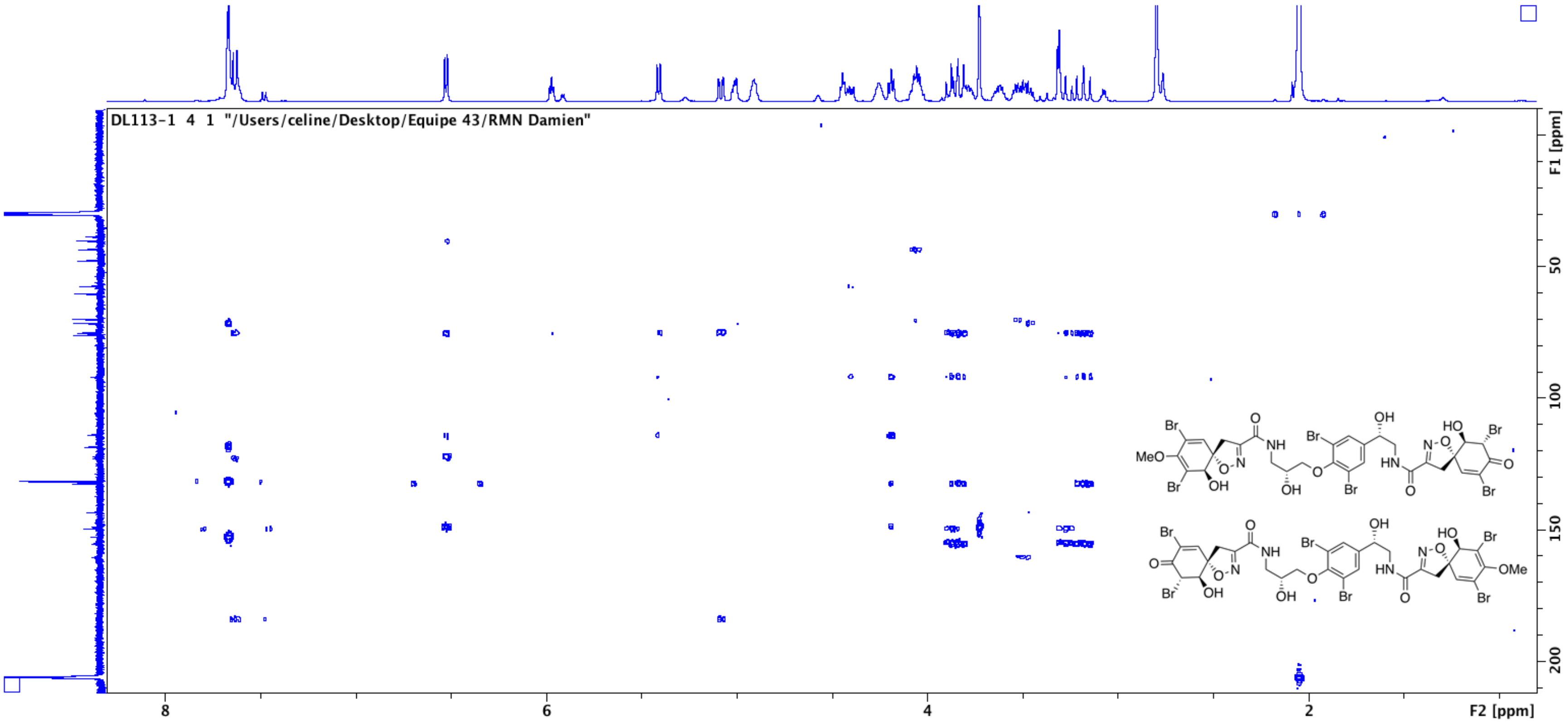

Figure S27: ^1H-^1H NOESY NMR spectrum of the mixture of suberein-3 (**4**) and suberein-4 (**5**) in acetone-d_6 (500 MHz).

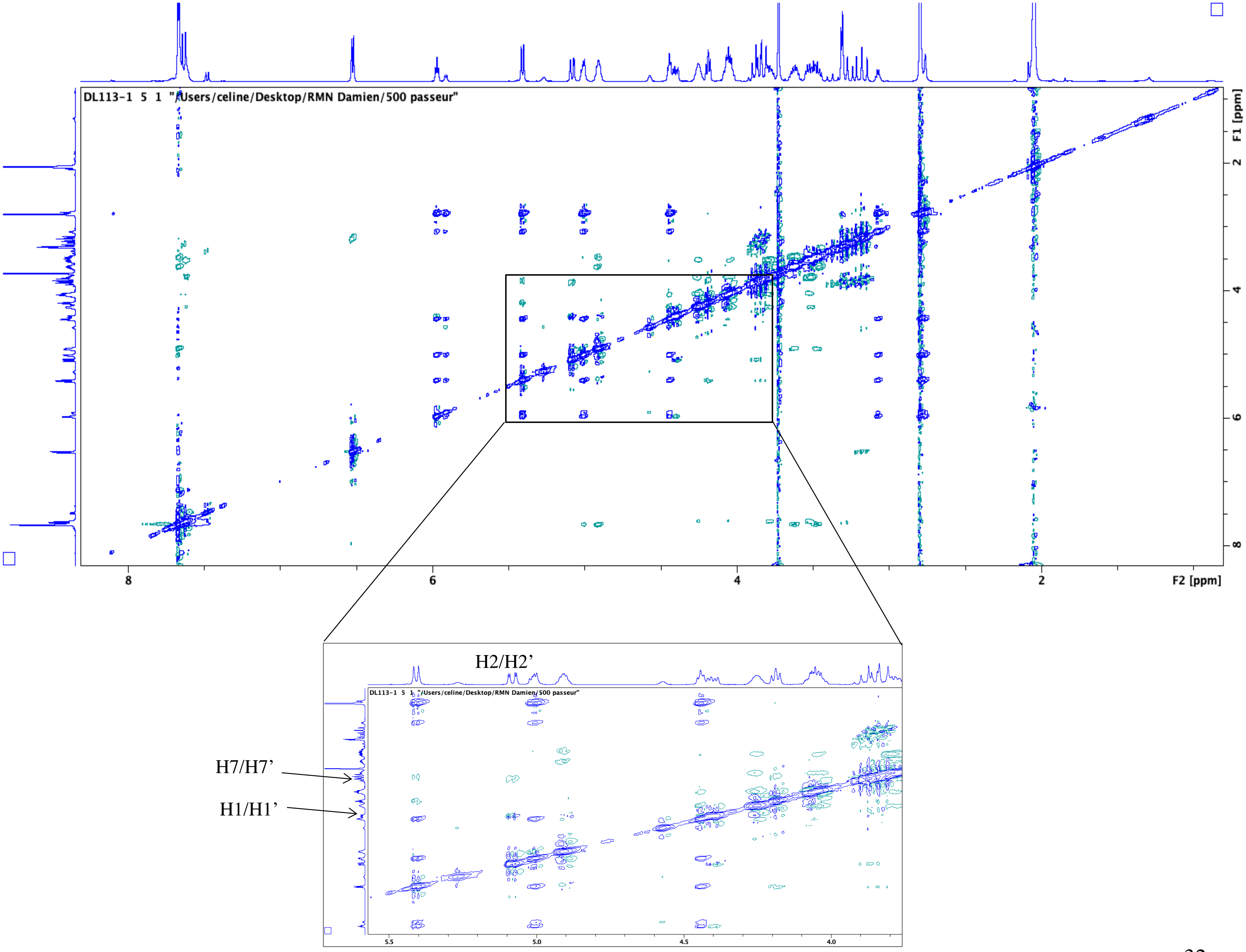

Figure S28: HR-ESI mass spectrum of the mixture of suberein-3 (**4**) and suberein-4 (**5**).

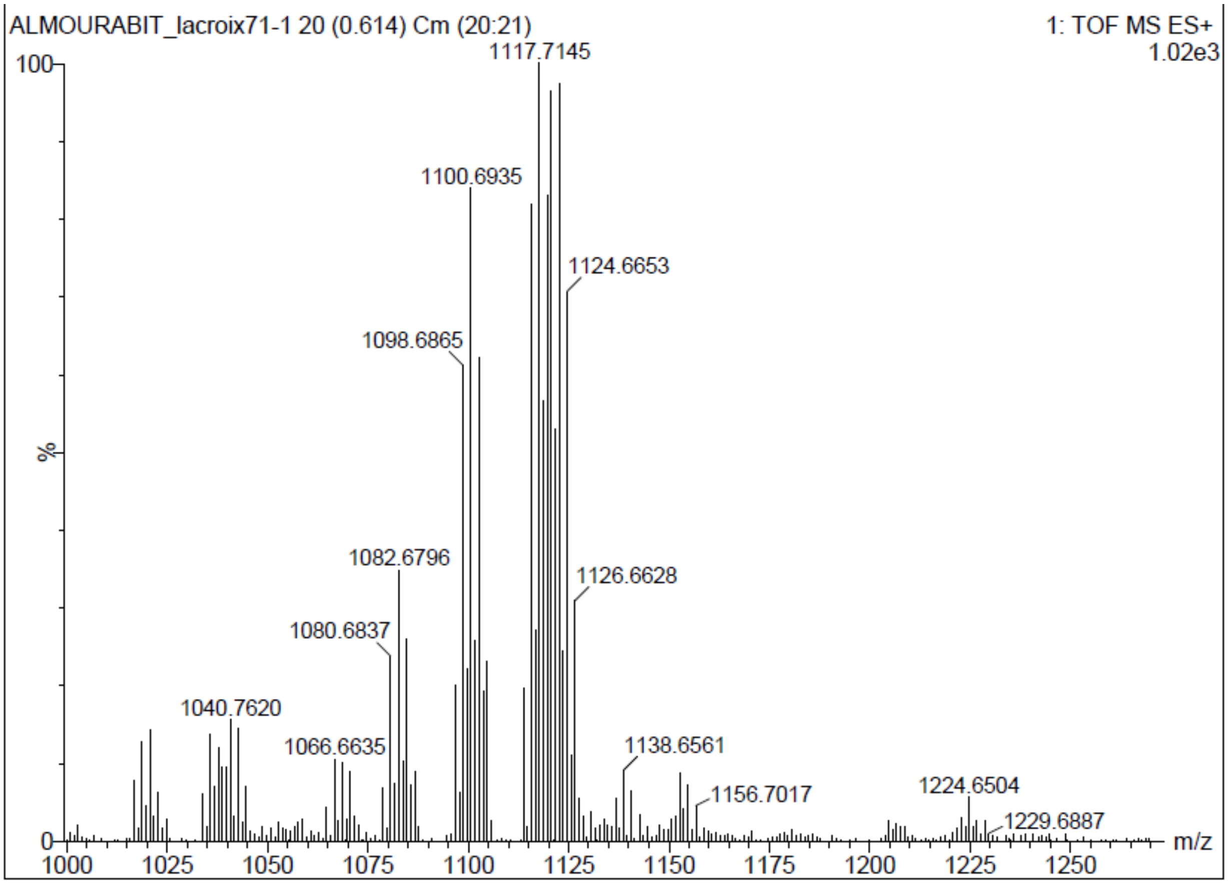

Figure S29: ^{1}H NMR spectrum of the mixture of suberein-5 (**6**) and suberein-6 (**7**) in acetone-d_6 (500 MHz).

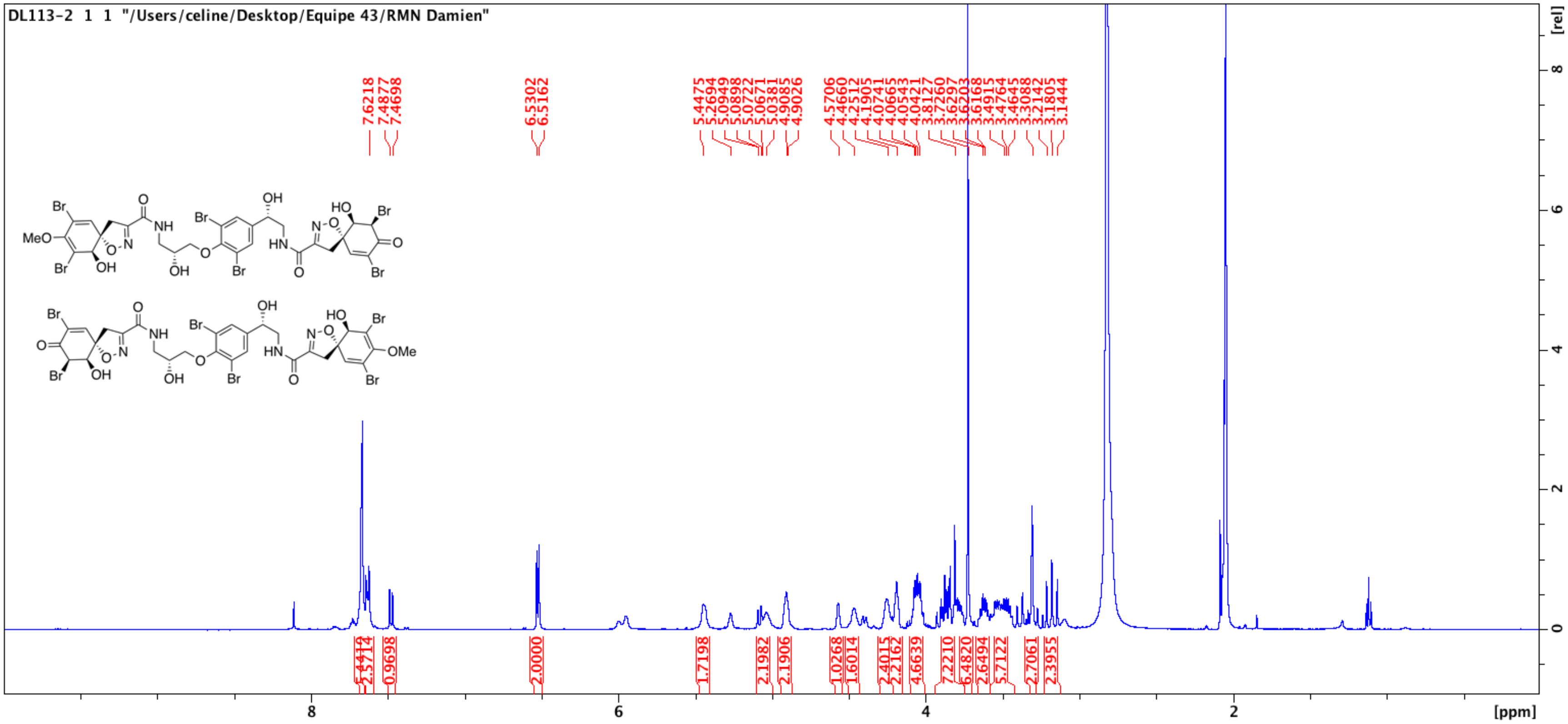

Figure S30: ^{13}C NMR spectrum of the mixture of suberein-5 (**6**) and suberein-6 (**7**) in acetone-d_6 (125 MHz).

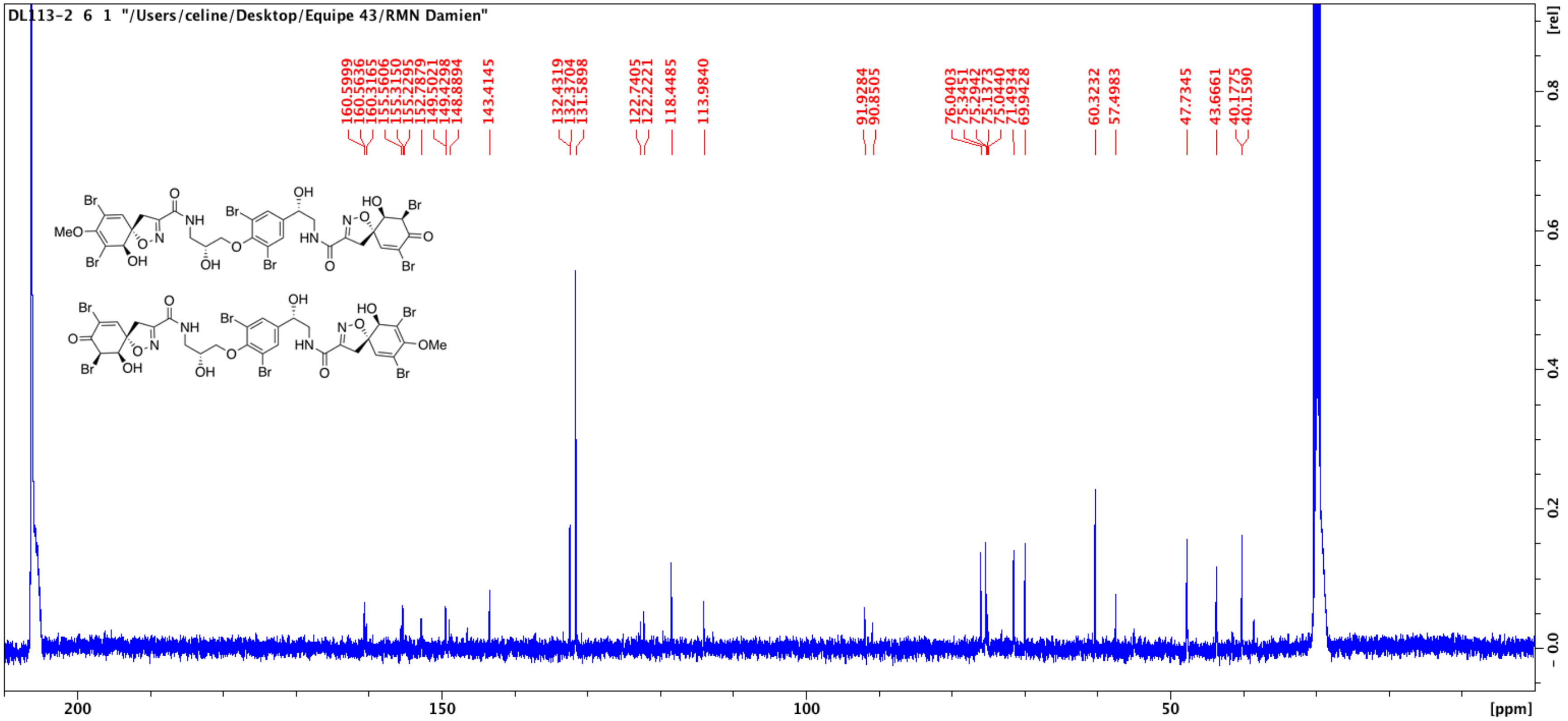

Figure S31: ^{1}H-^{1}H COSY NMR spectrum of the mixture of suberein-5 (**6**) and suberein-6 (**7**) in acetone-d_6 (500 MHz).

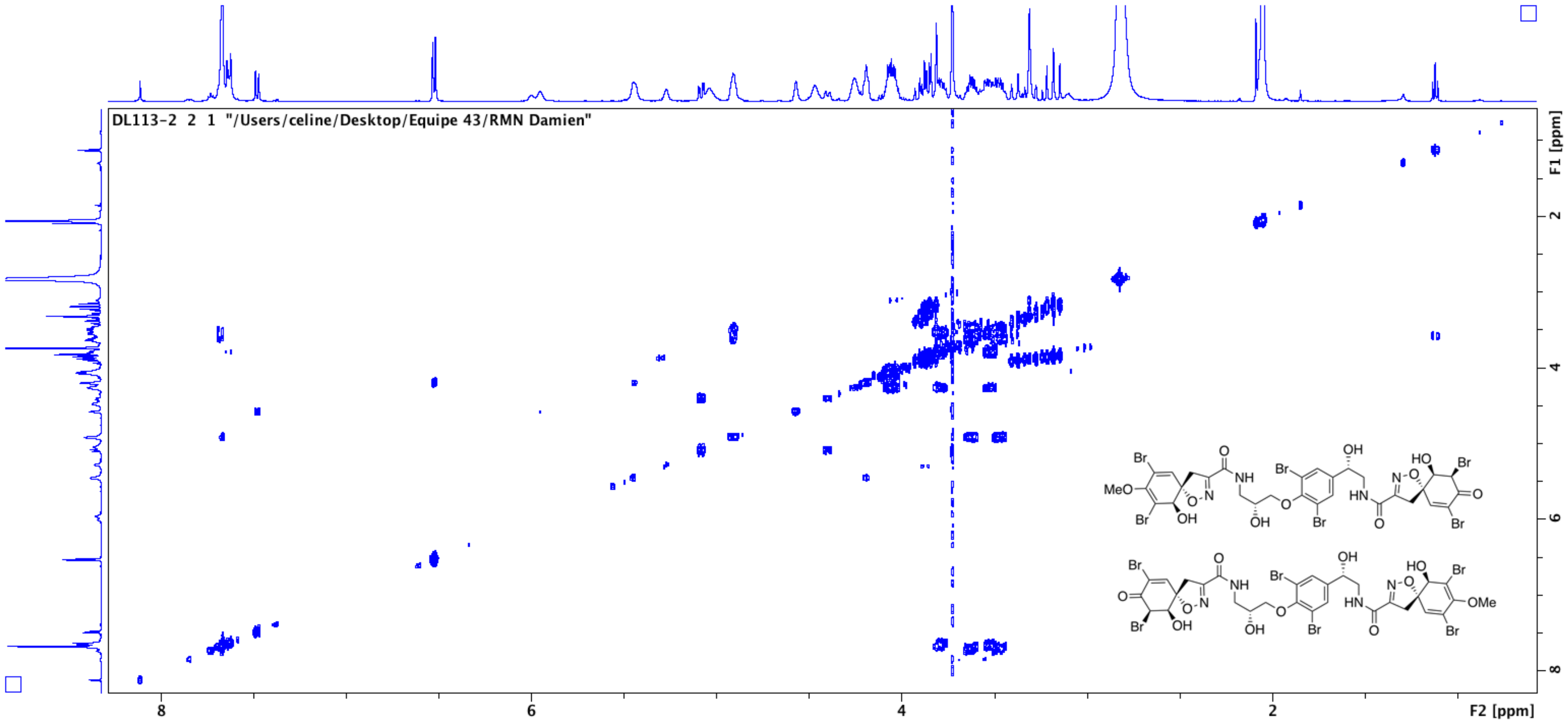

Figure S32: ^{1}H-^{13}C HSQC NMR spectrum of the mixture of suberein-5 (**6**) and suberein-6 (**7**) in acetone-d_6 (500 MHz).

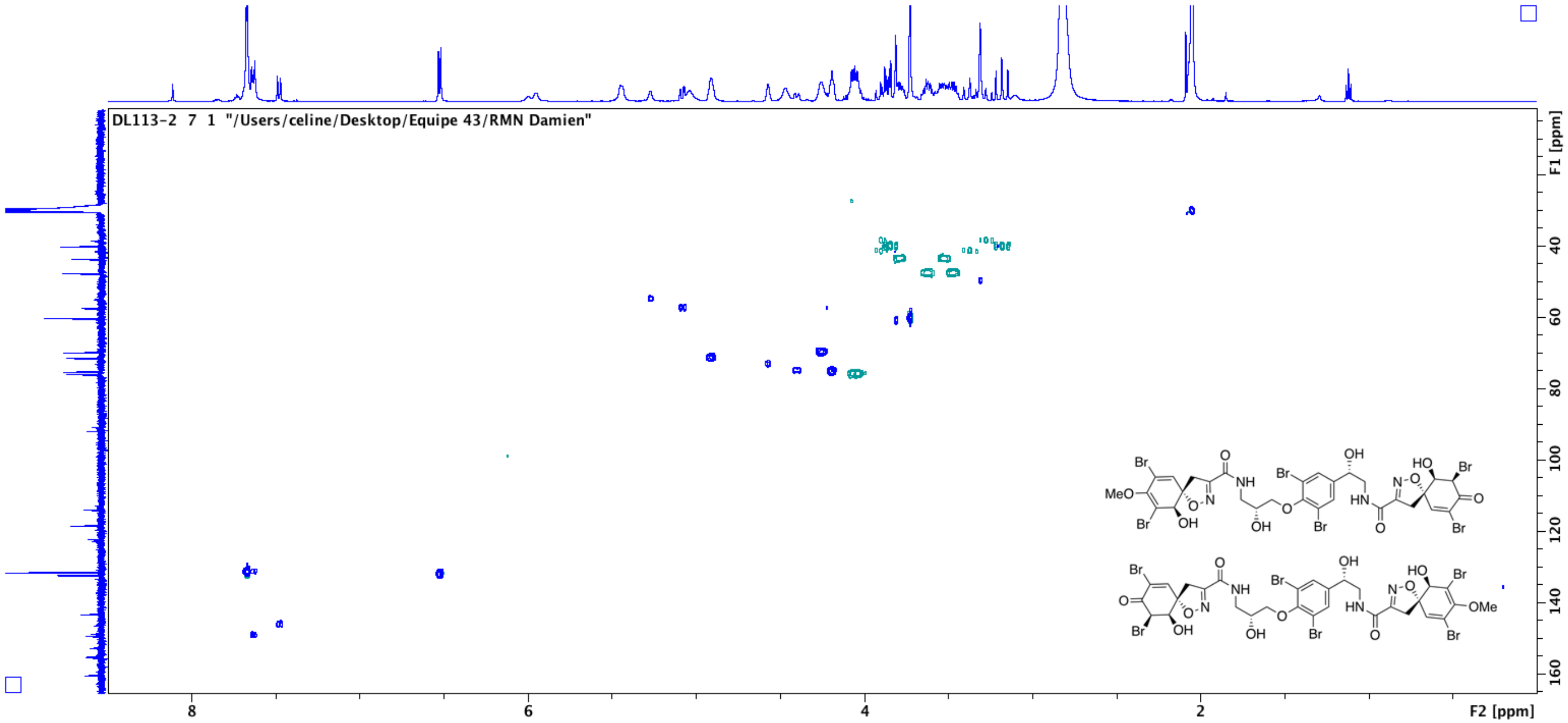

Figure S33: ^{1}H-^{13}C HMBC NMR spectrum of the mixture of suberein-5 (**6**) and suberein-6 (**7**) in acetone-d_6 (500 MHz).

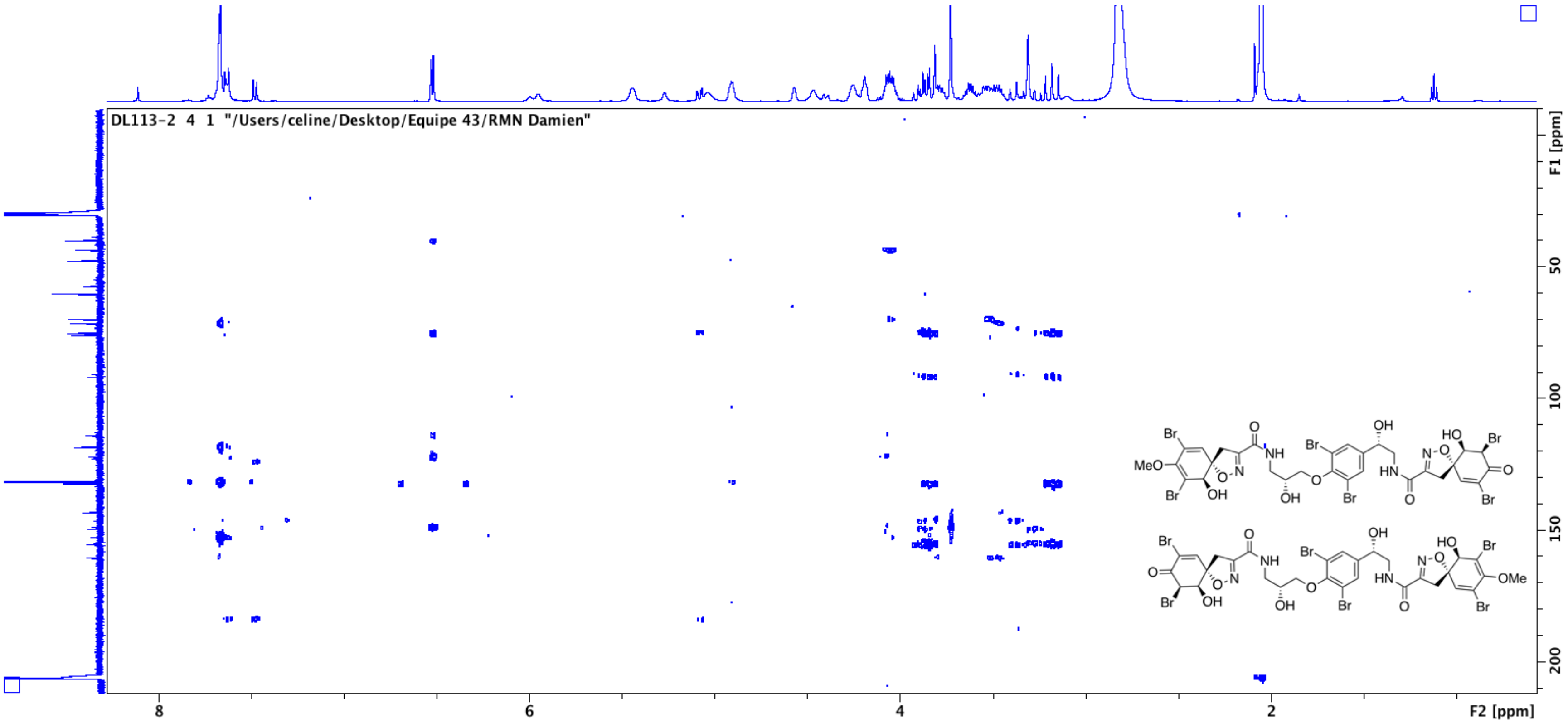

Figure S34: ^{1}H-^{1}H NOESY NMR spectrum of the mixture of suberein-5 (**6**) and suberein-6 (**7**) in acetone-*d*$_6$ (500 MHz).

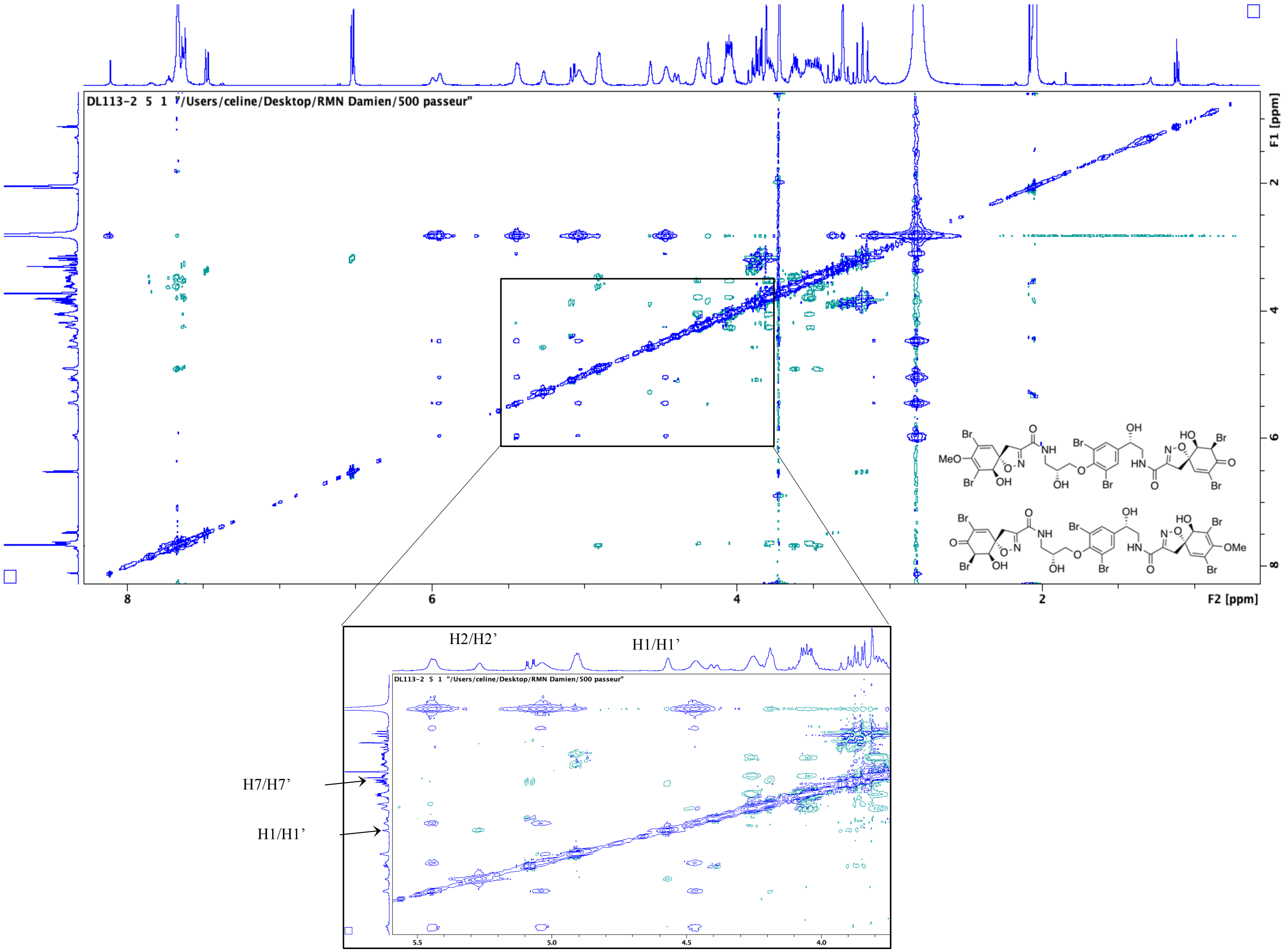

Figure S35: HR-ESI mass spectrum of the mixture of suberein-5 (**6**) and suberein-6 (**7**).

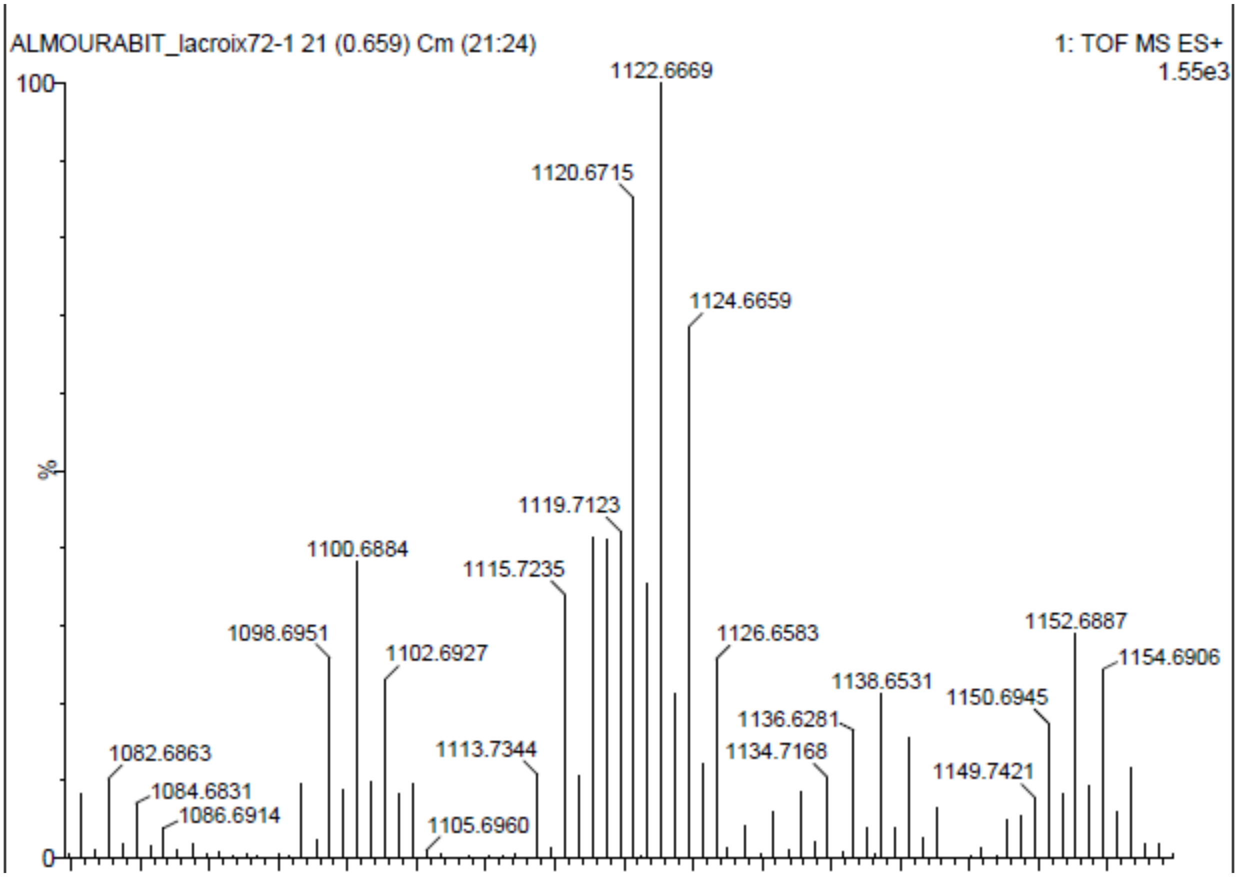

Figure S36: ^{1}H NMR spectrum of suberein-7 (**8**) in acetone-d_6 (500 MHz).

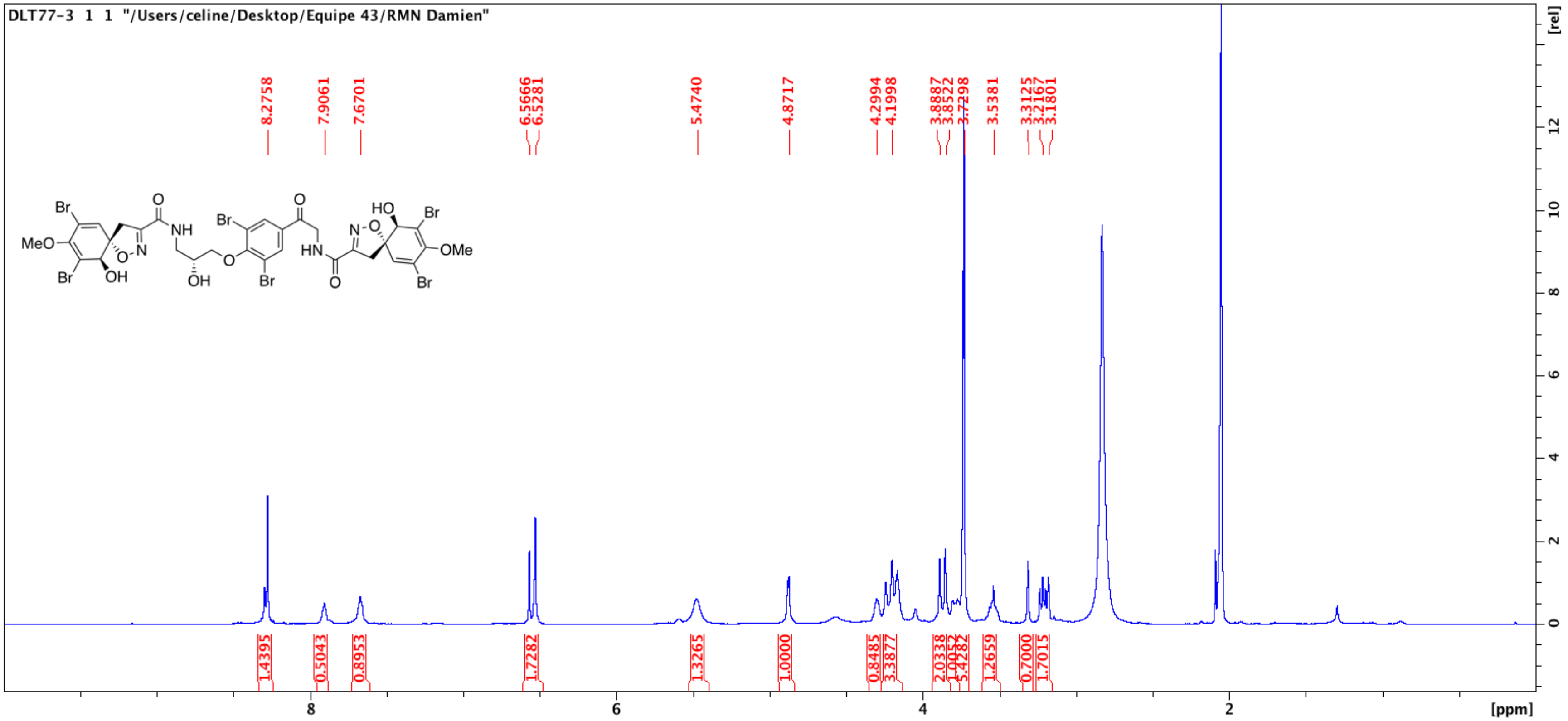

Figure S37: ^{13}C NMR spectrum of suberein-7 (**8**) in acetone-d_6 (125 MHz).

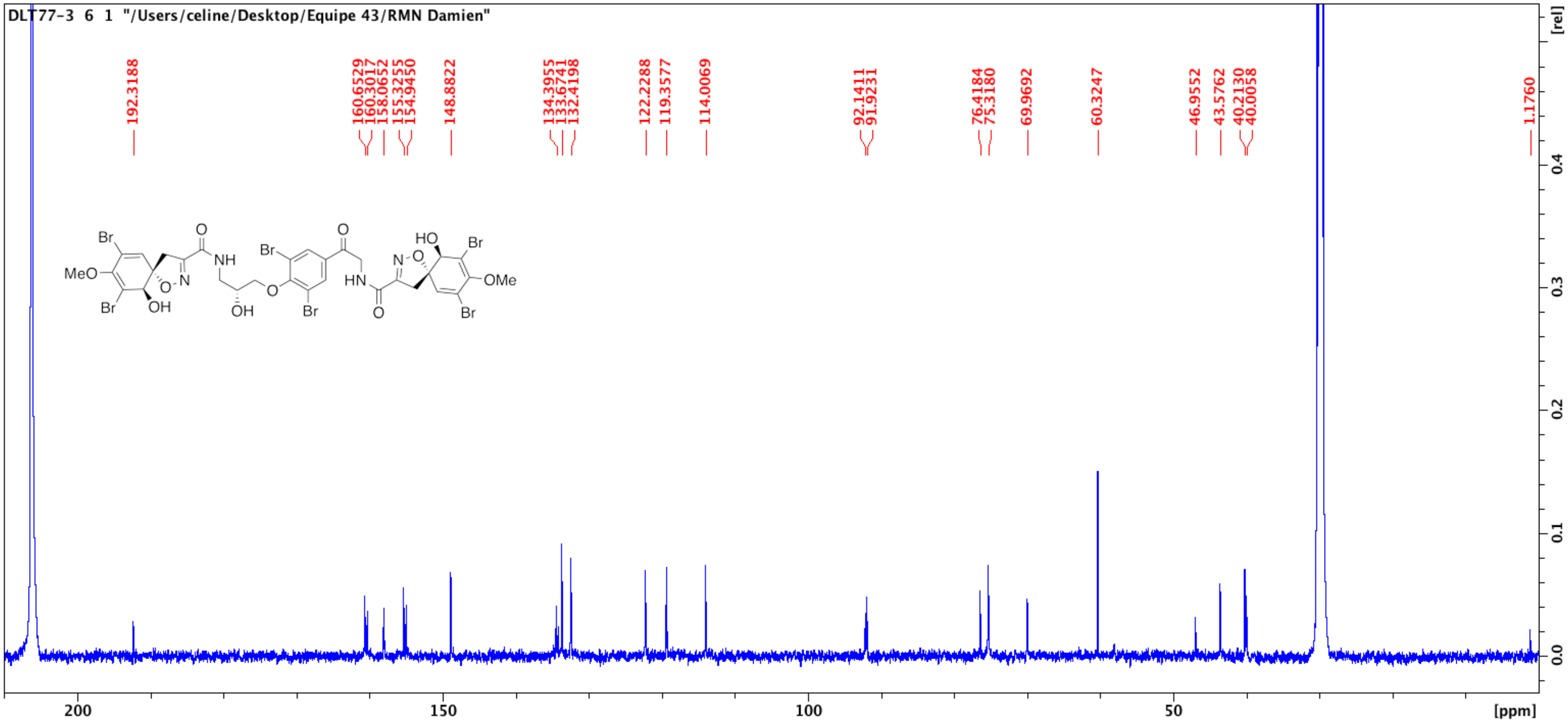

Figure S38: ^1H-^1H COSY NMR spectrum of suberein-7 (**8**) in acetone-d_6 (500 MHz).

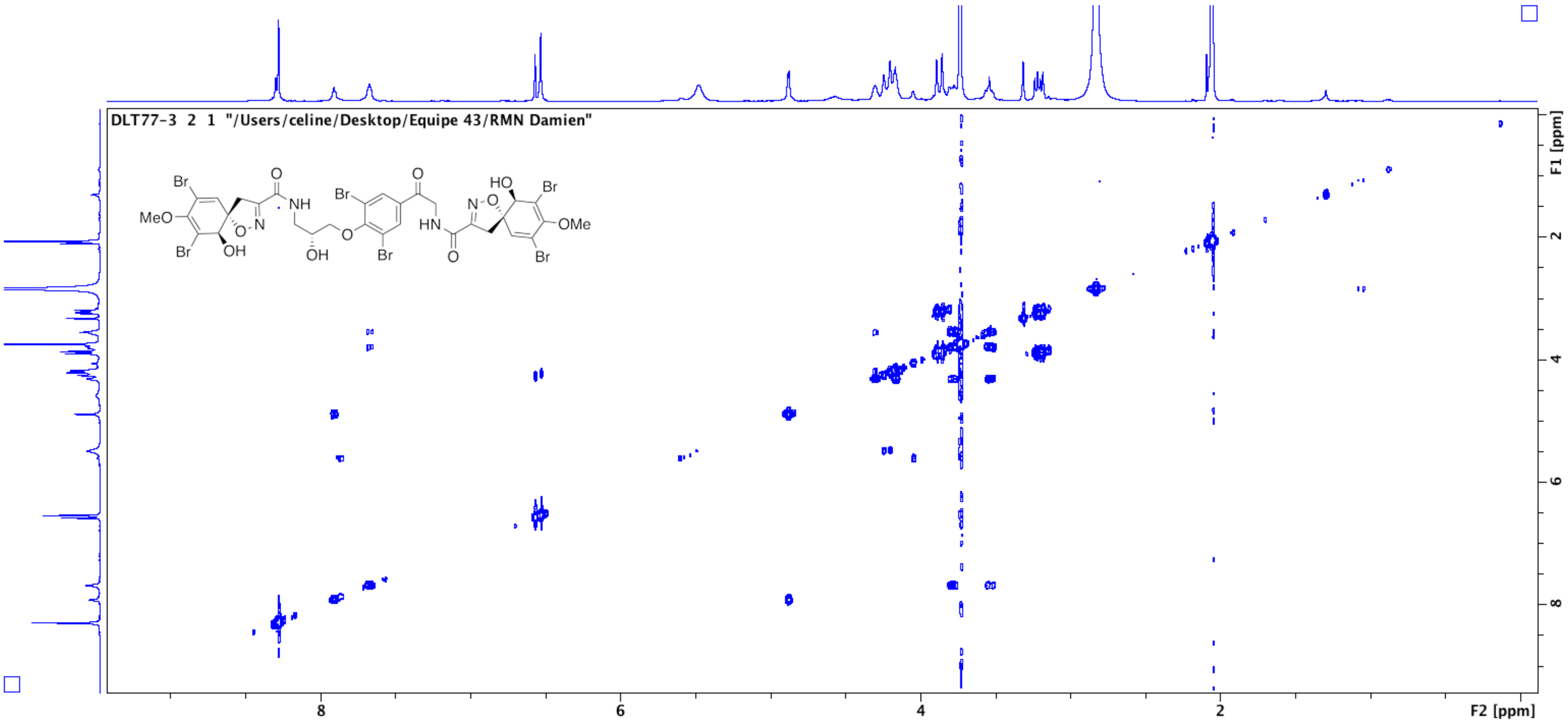

Figure S39: ^{1}H-^{13}C HSQC NMR spectrum of suberein-7 (**8**) in acetone-d_6 (500 MHz).

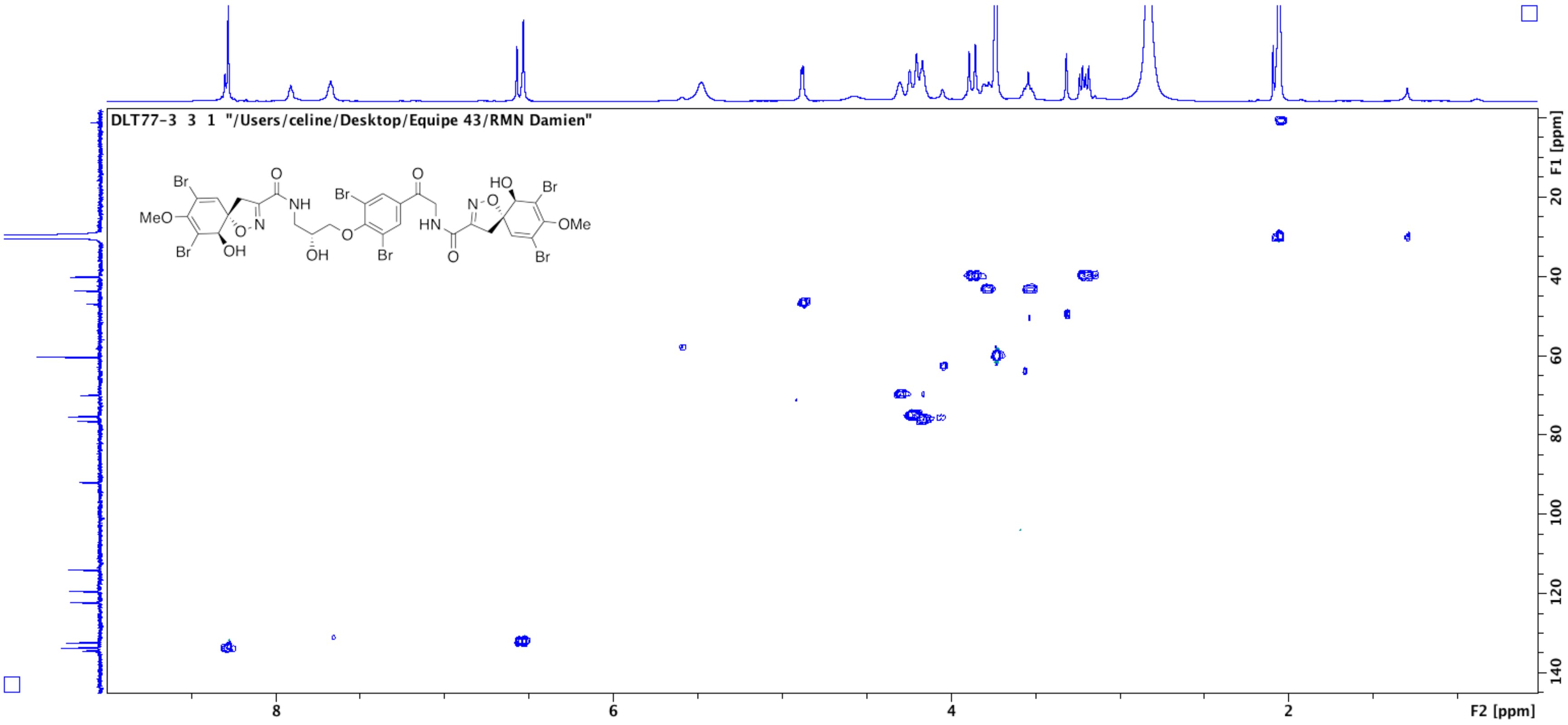

Figure S40: ^{1}H-^{13}C HMBC NMR spectrum of suberein-7 (**8**) in acetone-d_6 (500 MHz).

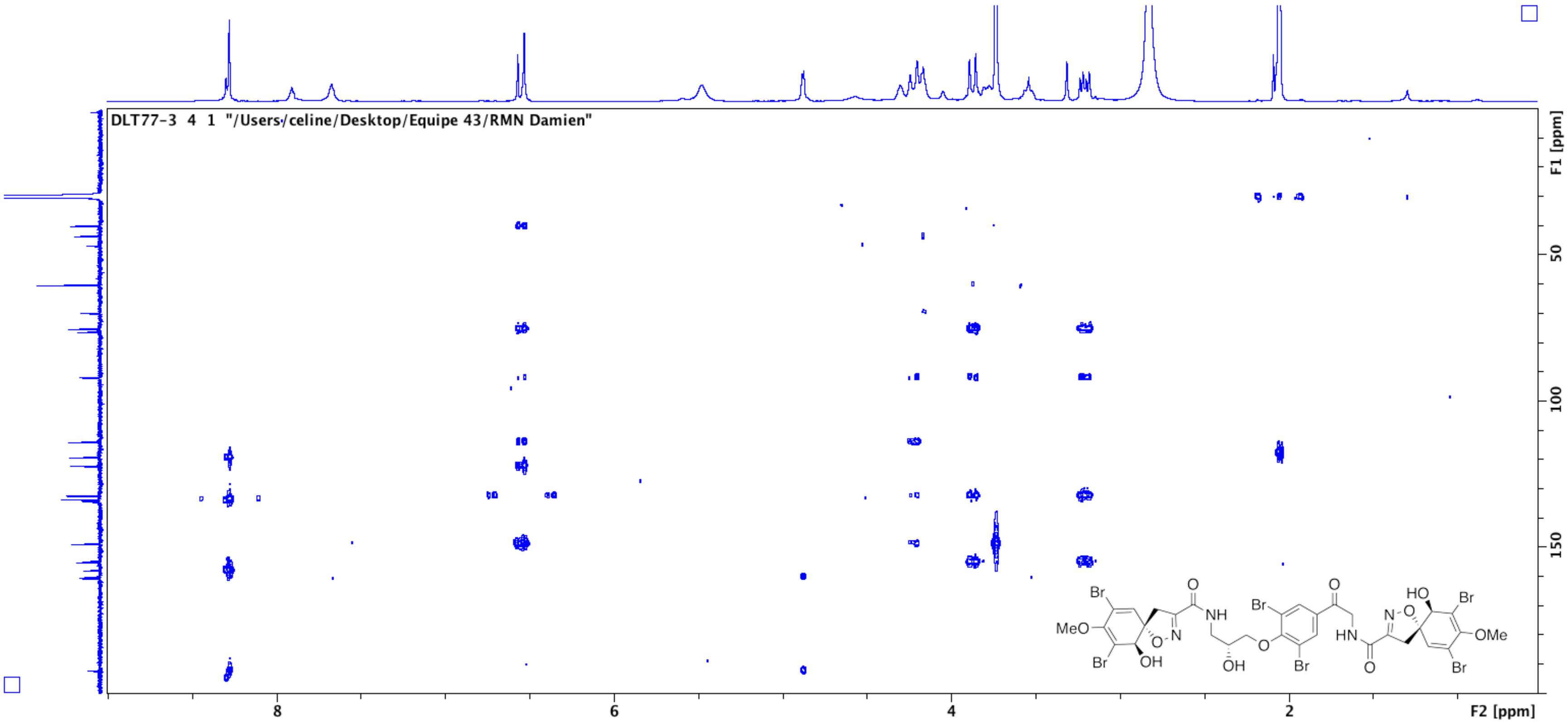

Figure S41: HR-ESI mass spectrum of suberein-7 (**8**).

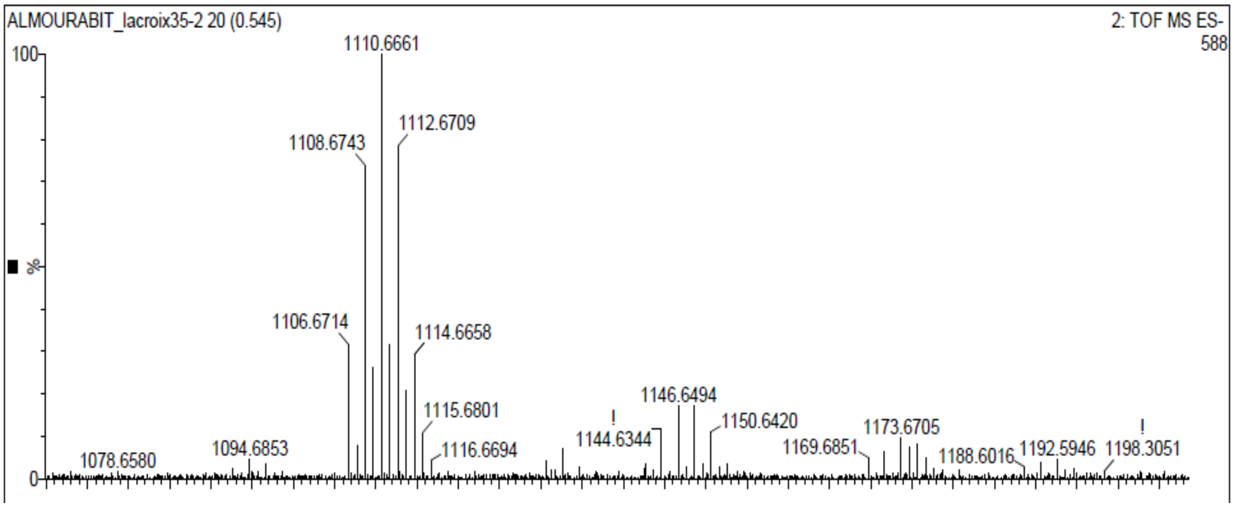

Figure S42: ECD spectrum of suberein-7 (**8**) in MeOH (c 0.15 mM).

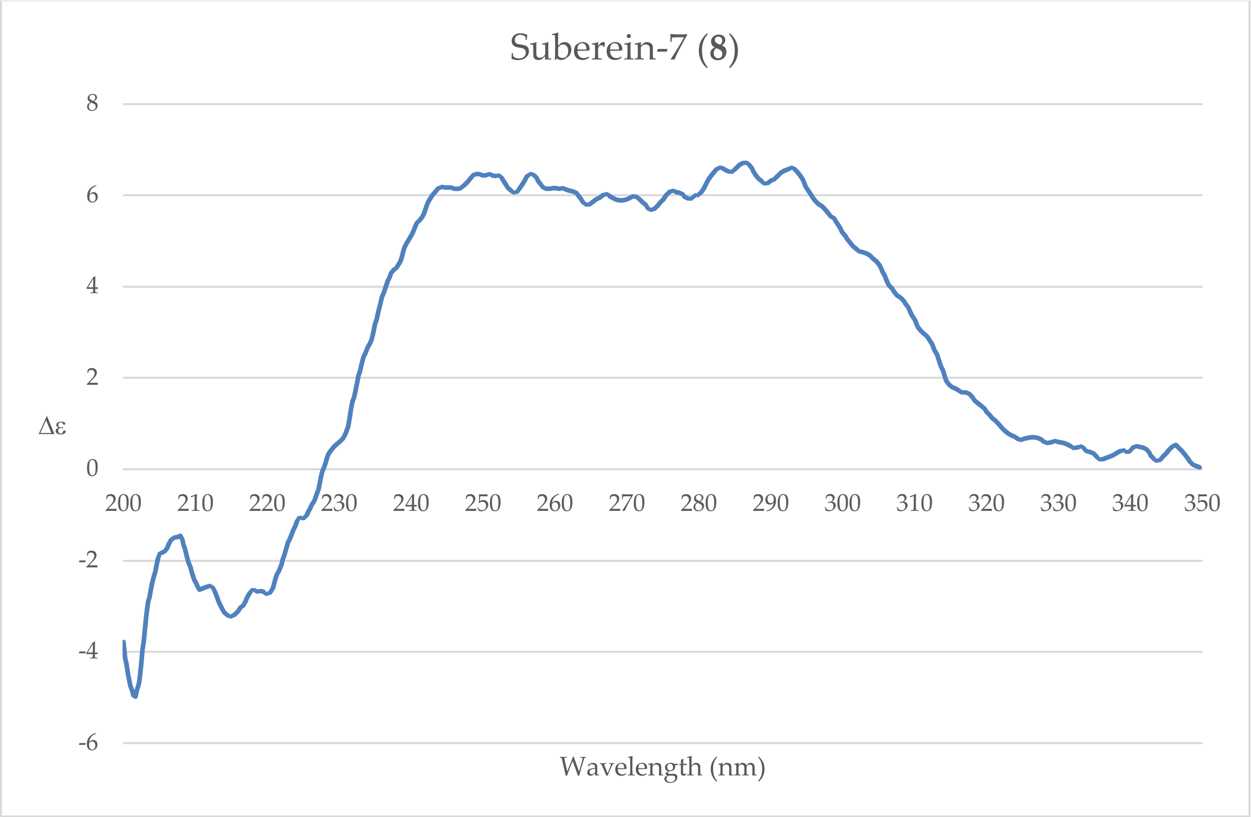

Figure S43: ^{1}H NMR spectrum of suberein-8 (**9**) in acetone-d_6 (500 MHz).

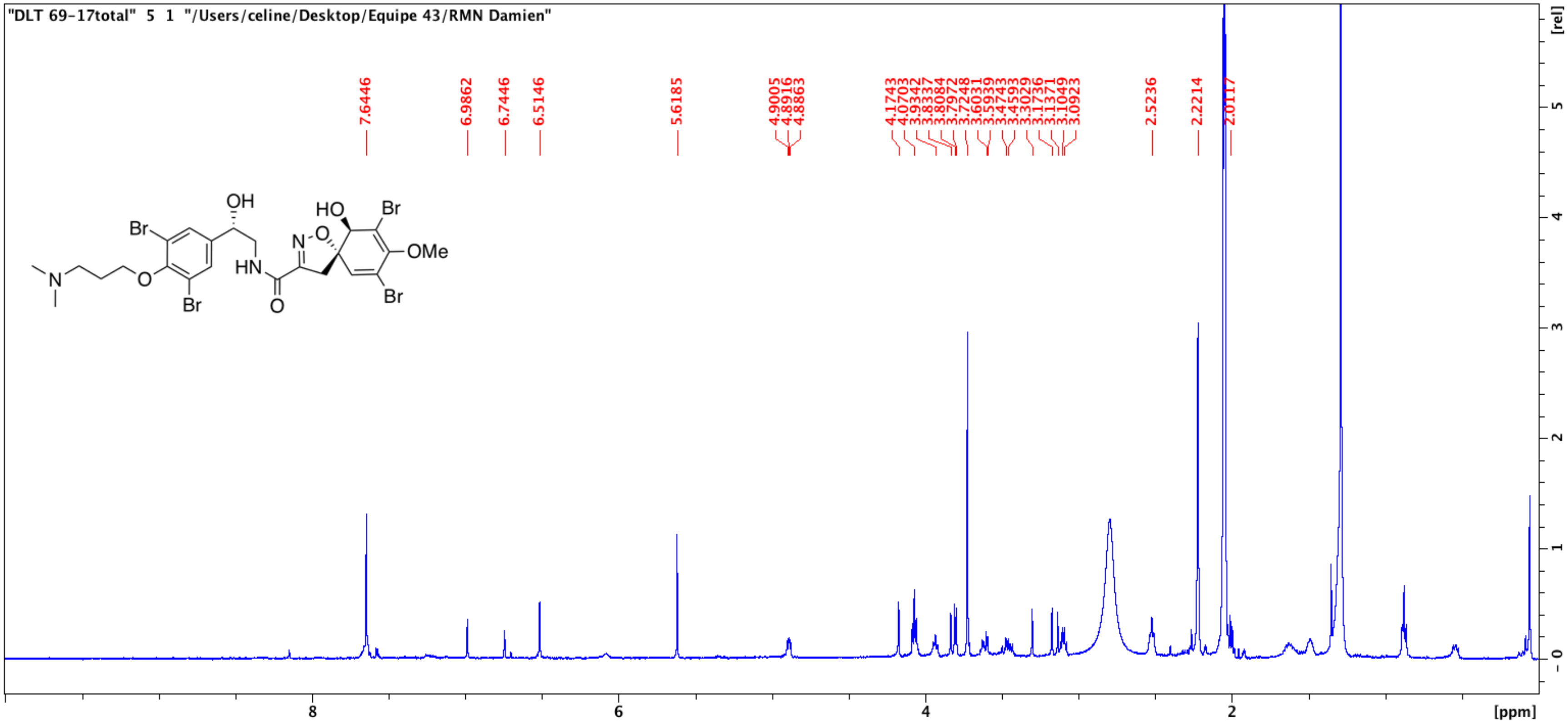

Figure S44: ^{13}C NMR spectrum of suberein-8 (**9**) in acetone-d_6 (125 MHz).

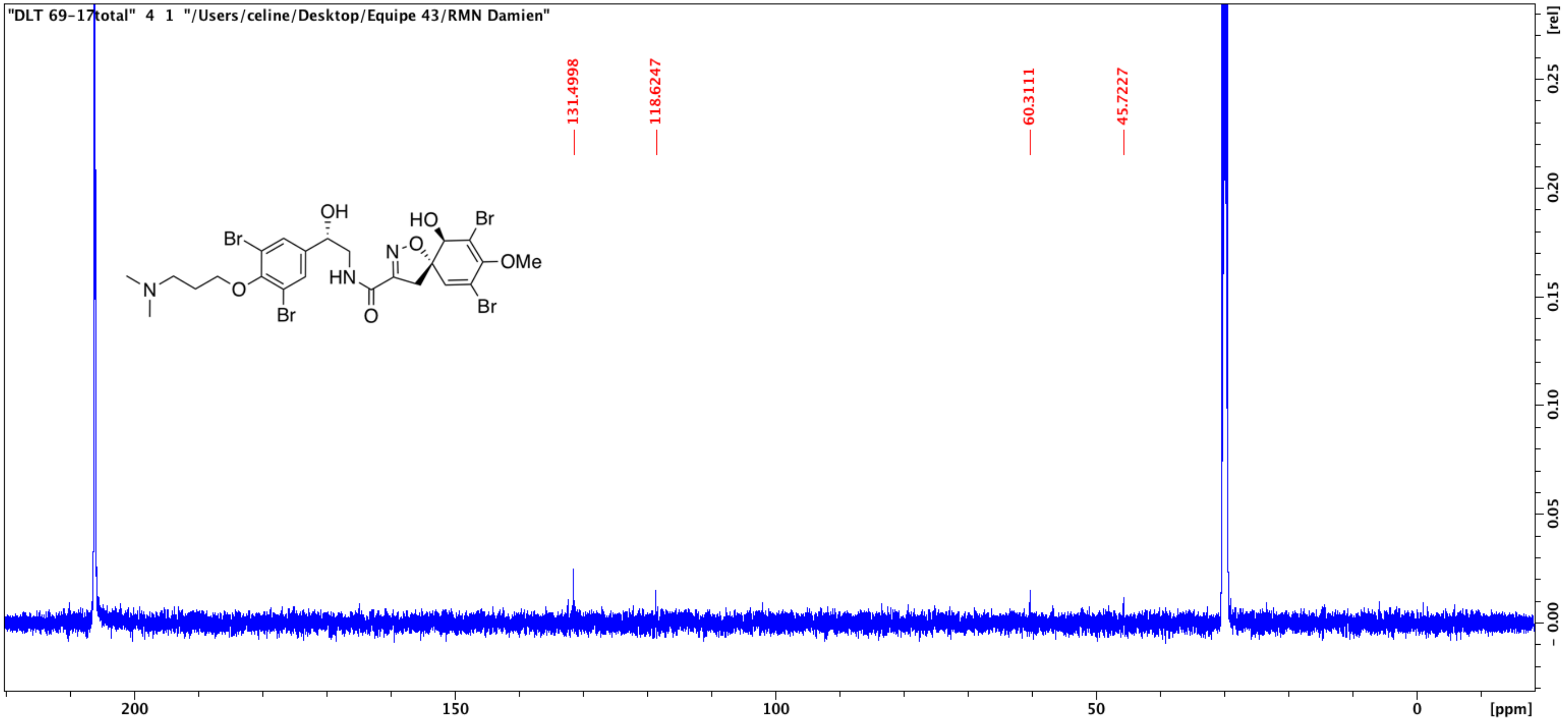

Figure S45: ^{1}H-^{1}H COSY NMR spectrum of suberein-8 (**9**) in acetone-d_6 (500 MHz).

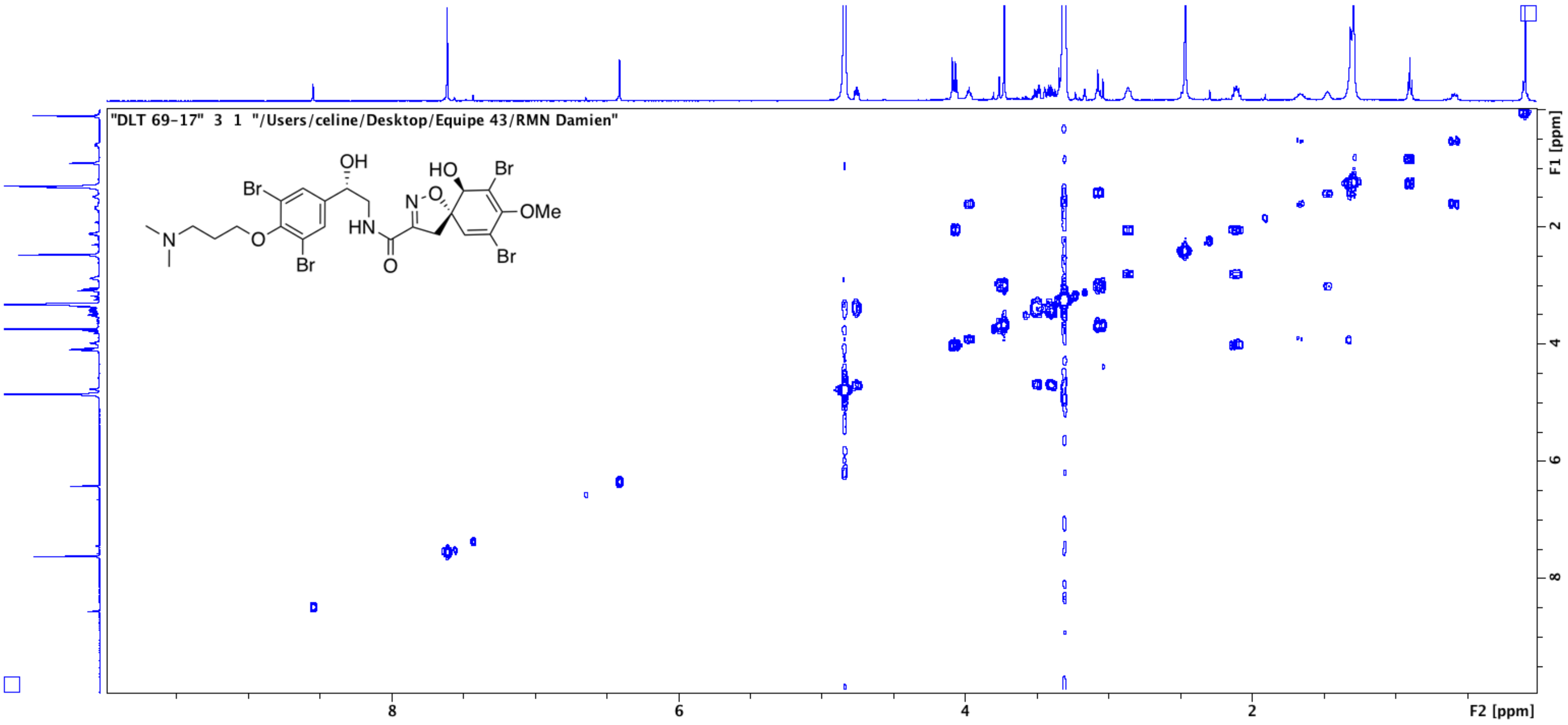

Figure S46: ^{1}H-^{13}C HSQC NMR spectrum of suberein-8 (**9**) in acetone-d_6 (500 MHz).

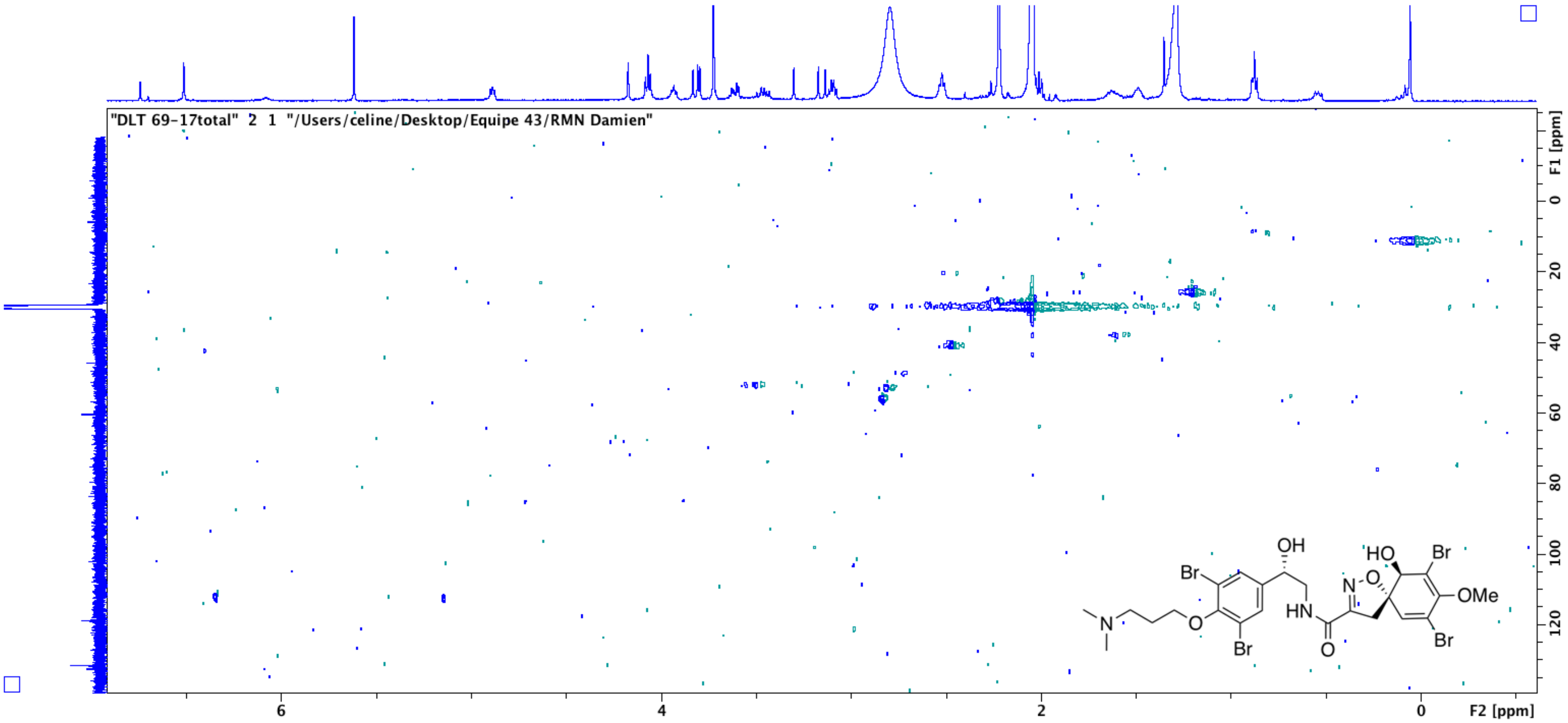

Figure S47: ^{1}H-^{13}C HMBC NMR spectrum of suberein-8 (**9**) in acetone-d_6 (500 MHz).

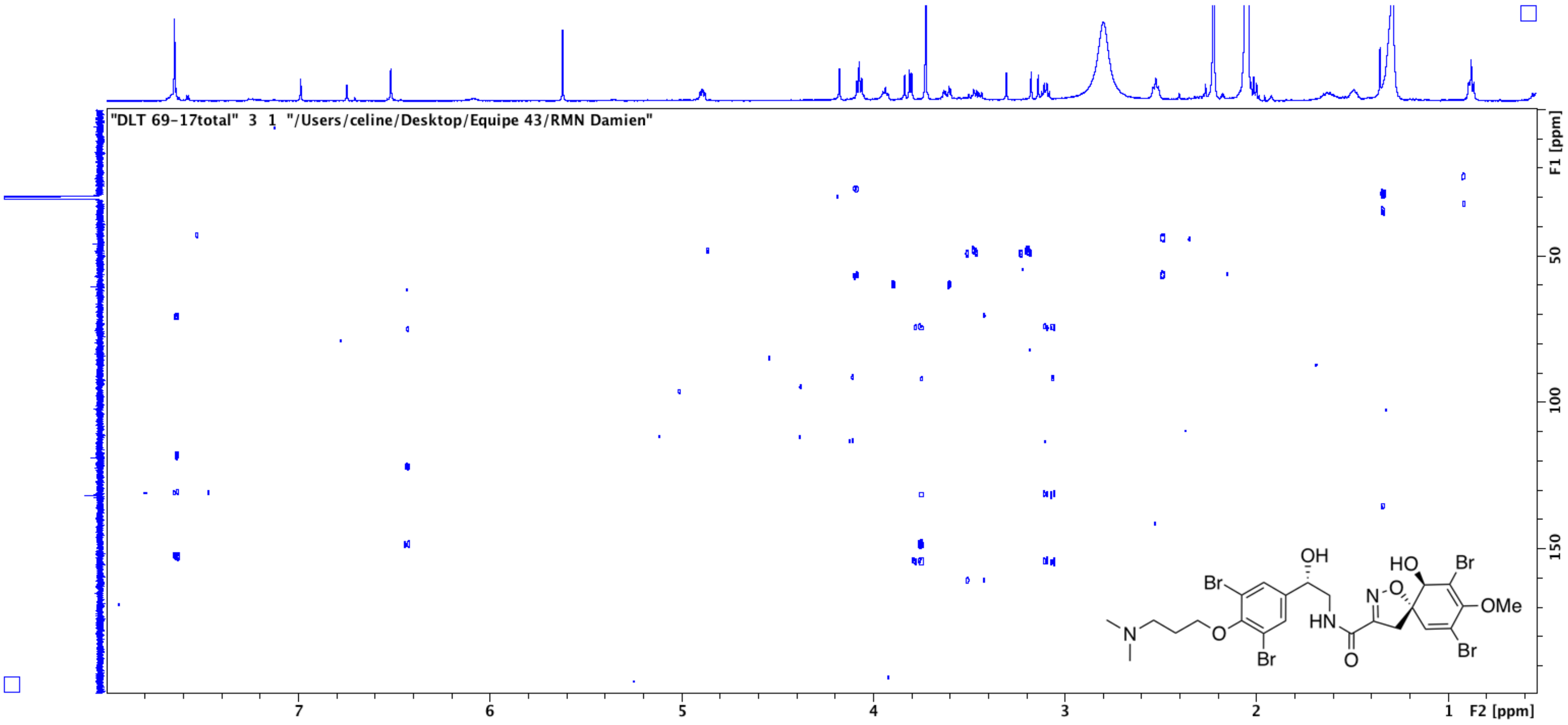

Figure S48: HR-ESI mass spectrum of suberein-8 (**9**).

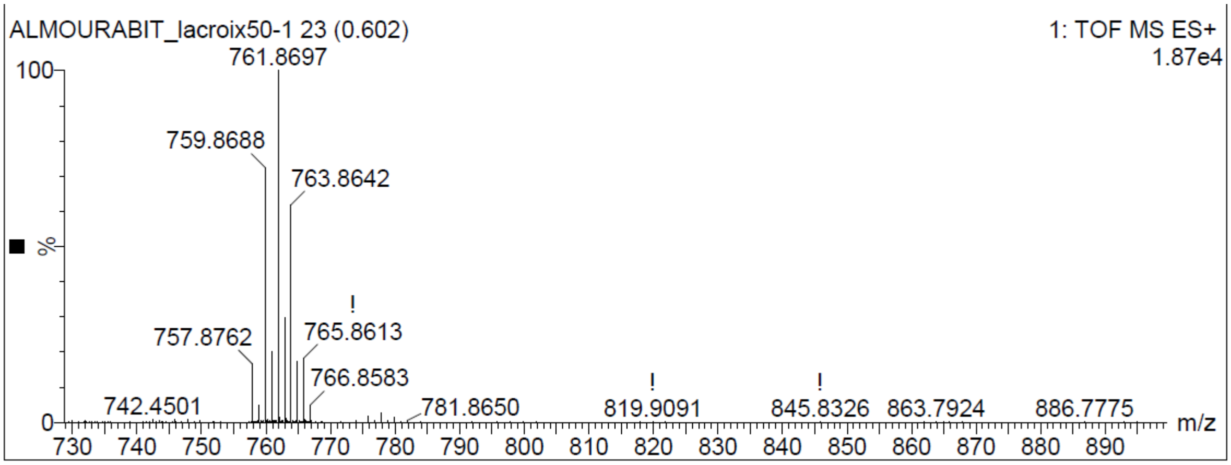

Figure S49: ^{1}H NMR spectrum of 17-deoxy-*epi*-fistularin-3 (**10**) in acetone-d_6 (500 MHz).

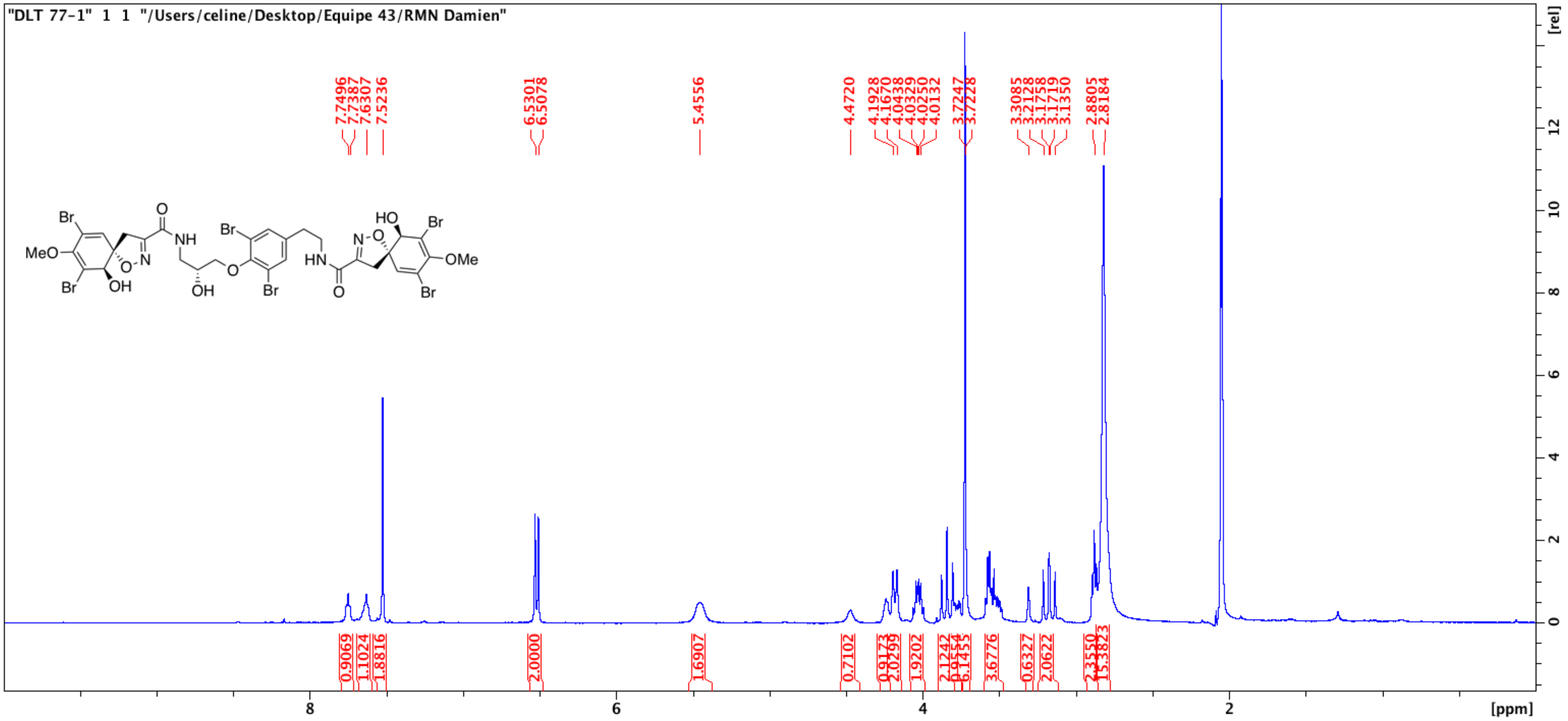

Figure S50: HR-ESI mass spectrum of 17-deoxy-*epi*-fistularin-3 (**10**).

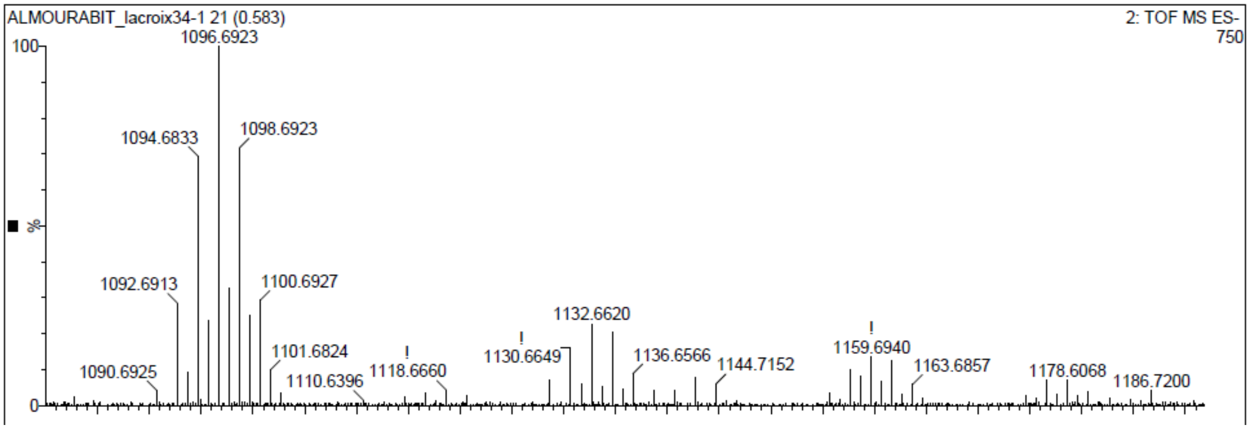

Figure S51: ECD spectrum of 17-deoxy-*epi*-fistularin-3 (**10**) in MeOH (c 0.15 mM).

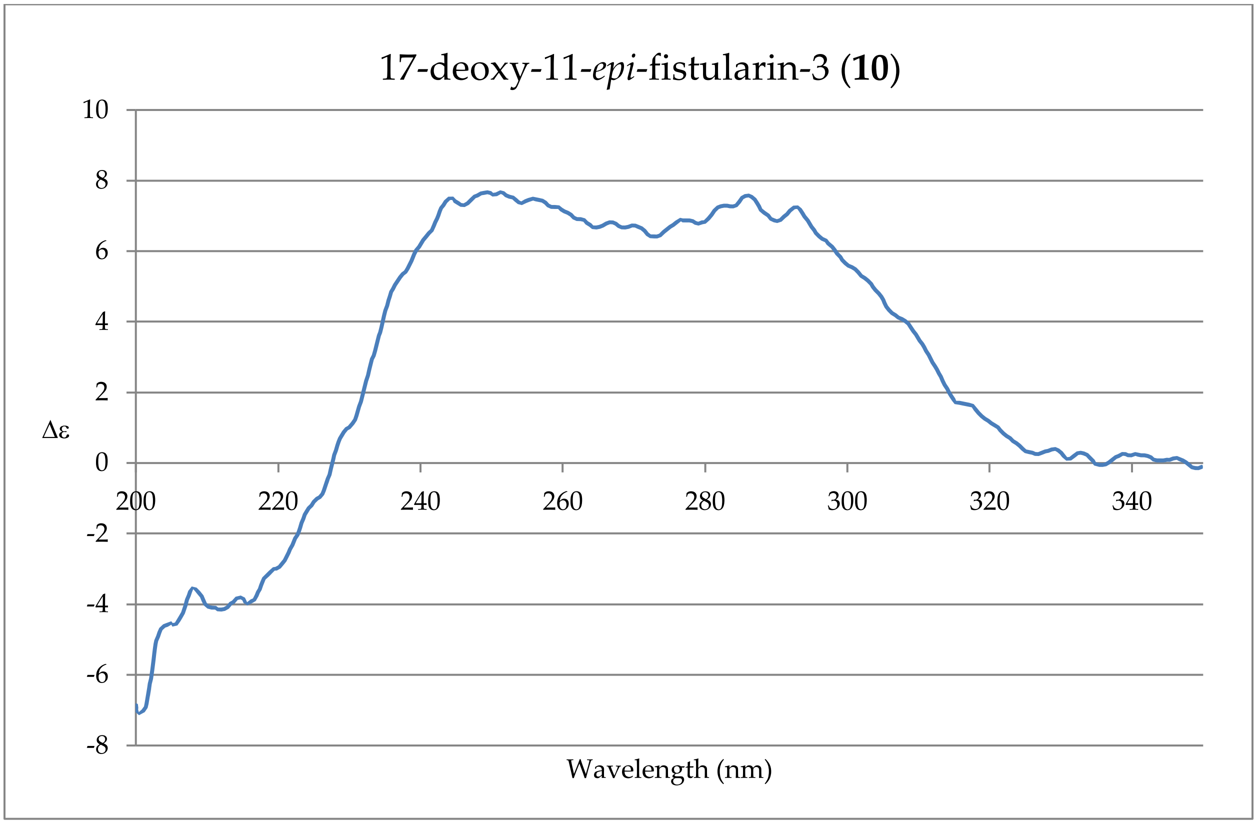

Supplement: Supplementary file 1 [file marinedrugs-19-00143-s001.pdf]
